# Supplementary material for: Developmental beta-cell death orchestrates the islet’s inflammatory milieu by regulating immune system crosstalk
Source: EMBO J. 2025 Jan 6;44(4):1131–53. doi: 10.1038/s44318-024-00332-w (PMC11833124; doi:10.1038/s44318-024-00332-w)
Supplement: Supplementary file 1 — Appendix [file 44318_2024_332_MOESM1_ESM.pdf]

Appendix

This file includes:

Appendix figures and figure legends 1-11 (pages 2-24)

Appendix Supplementary Materials (pages 25-31)

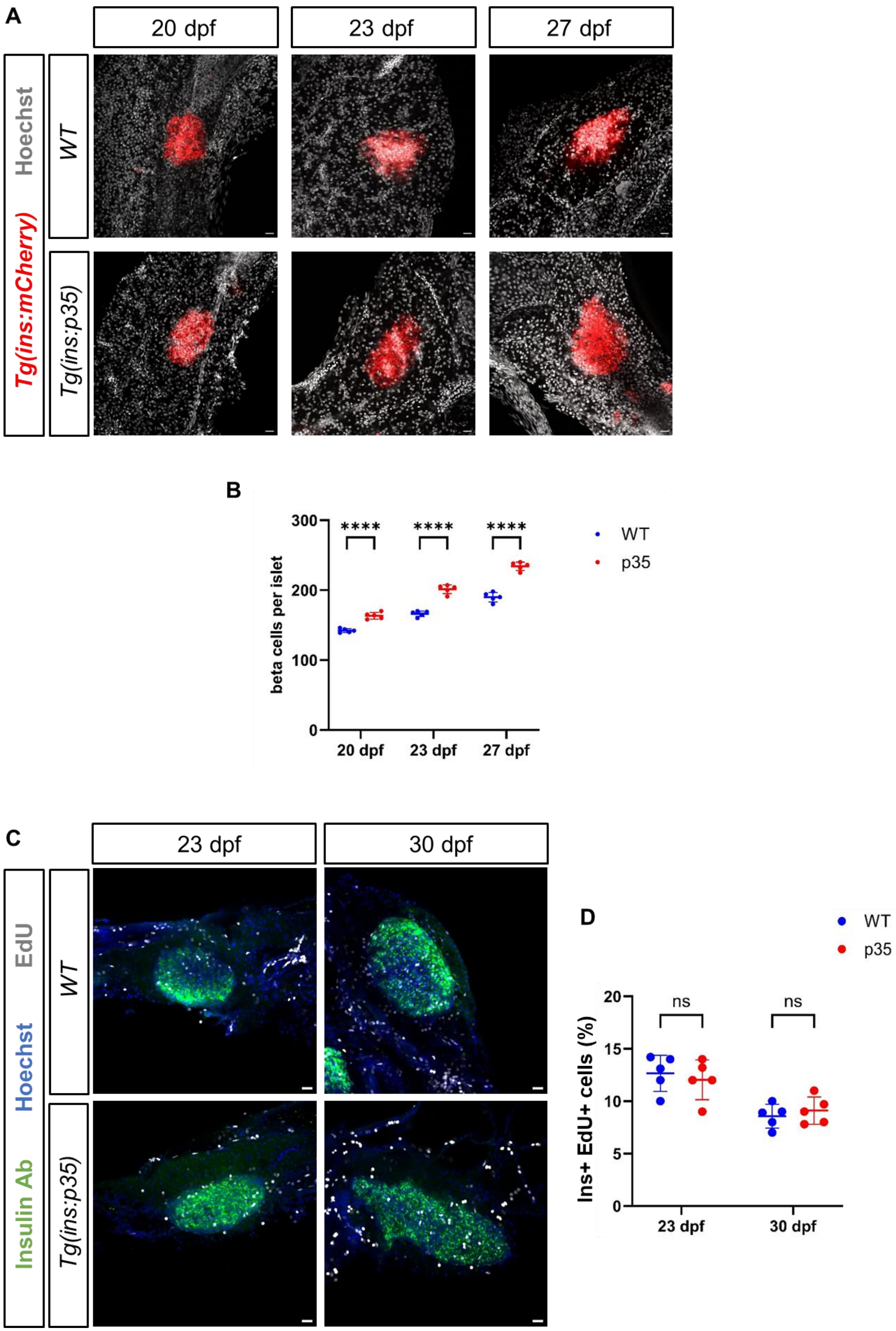
A
20 dpf
23 dpf
27 dpf
Tg(ins:mCherry) Hoechst
WT
Tg(ins:p35)
B
beta cells per islet
300
200
100
0
****
20 dpf
23 dpf
27 dpf
WT
p35
C
23 dpf
30 dpf
Insulin Ab Hoechst EdU
WT
Tg(ins:p35)
D
WT
p35
Ins+ EdU+ cells (%)
20
15
10
5
0
ns
23 dpf
30 dpf

Appendix Figure S1: (A) Representative confocal images (maximum intensity projection) of primary islets from 20, 23 and 27 dpf WT and *Tg(ins:p35)* animals in the transgenic background of *Tg(ins:mCherry)* reporter (in red). **(B)** Quantification showing number of mCherry+ beta cells per islet in WT and p35 transgenic at 20, 23 and 27 dpf. Horizontal bars represent mean values. Scale bar, 20µm. Error bars are mean ± SD from n=at least 5 independent samples per group at each developmental time point. Unpaired two tailed *t*-test with Welch's correction, ****P=0.0000748 **(C)** 23 and 30 dpf WT and *Tg(ins:p35)* animals were incubated in 2 mM EdU for 24 hours. EdU incorporation assay was performed to mark the proliferating beta cells. Representative confocal images (maximum intensity projection) of primary islet from WT and *Tg(ins:p35)* animal. Beta cells are labelled using an insulin antibody (in green), the EdU positive cells (in grey) while the nuclei are stained with Hoechst dye (in blue). **(D)** Quantification showing the percentage of EdU+ insulin+ cells per islet in WT and p35 transgenic at 23 and 30 dpf. Horizontal bars represents mean values. Error bars are mean ± SD from n=at 5 independent samples per group at each developmental time point. Unpaired two tailed *t*-test with Welch's correction, ns-not significant.

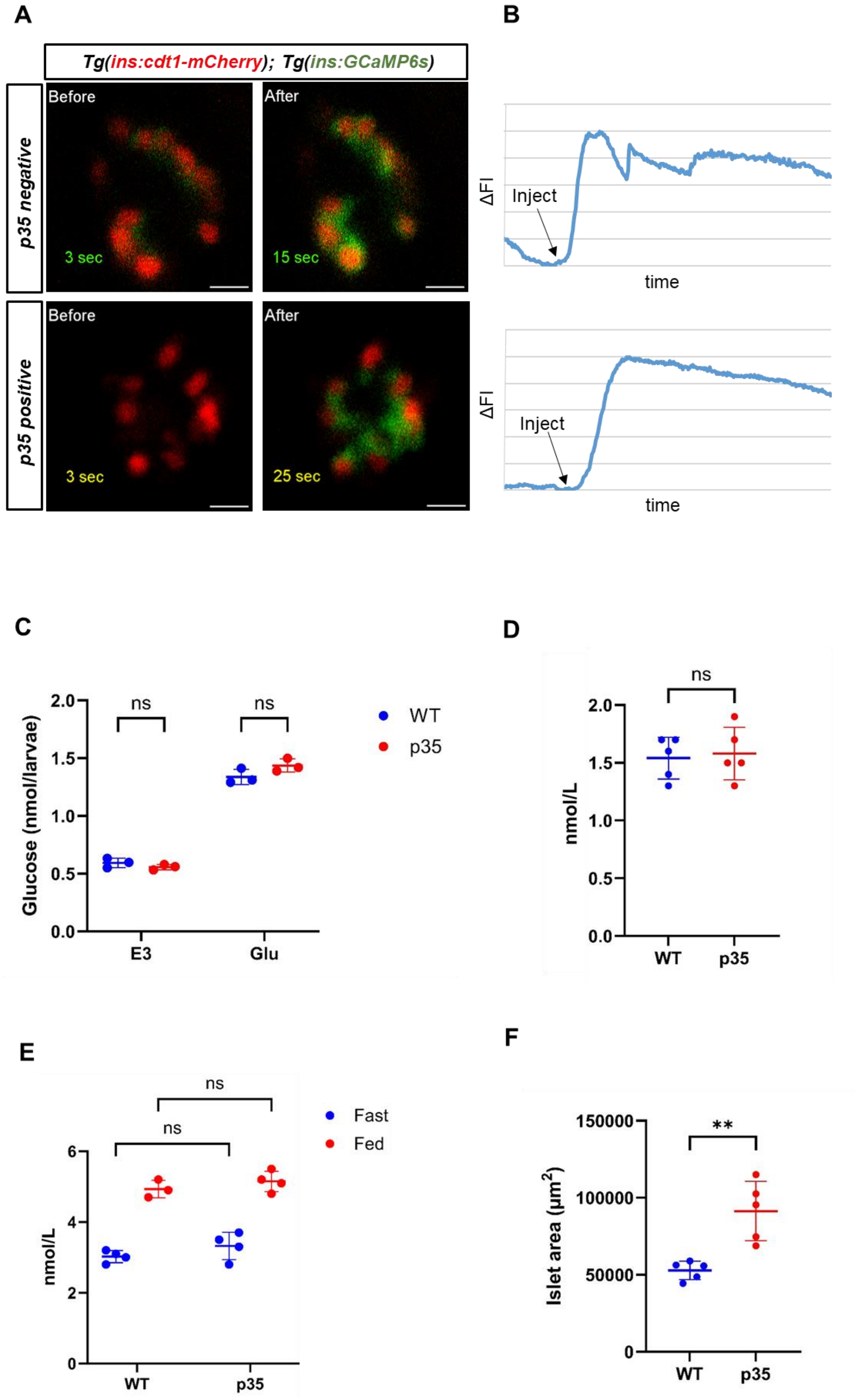
A
Tg(ins:cdt1-mCherry); Tg(ins:GCaMP6s)
p35 negative
Before
After
3 sec
15 sec
p35 positive
Before
After
3 sec
25 sec
B
ΔFI
Inject
time
ΔFI
Inject
time
C
Glucose (nmol/larvae)
2.0
1.5
1.0
0.5
0.0
ns
ns
E3
Glu
WT
p35
D
ns
nmol/L
2.0
1.5
1.0
0.5
0.0
WT
p35
E
ns
ns
nmol/L
6
4
2
0
WT
p35
Fast
Fed
F
Islet area (µm2)
150000
100000
50000
0
**
WT
p35

Appendix Figure S2: Genetic overexpression of p35 does not alter beta cell functionality.

(A) Snapshots from live imaging of larvae expressing GCaMP6s and nuclear mCherry in the beta cells. The images show the GCaMP6s fluorescence before and after stimulation with 12.5mM glucose solution. **(B)** Traces of GCaMP6s fluorescence intensity over time for the islets shown in A. The islets of WT and *Tg(ins:p35)* larvae show a synchronized response to glucose stimulation. n = 5 independent samples per group. **(C)** Plot showing whole larvae glucose levels in WT and *Tg(ins:p35)* larvae in fish water (E3) and after 48 hour incubation with 130mM glucose. There was no significant difference in the glucose levels of WT and *Tg(ins:p35)* larvae. Error bars are mean ± SD from n=3 independent samples per group. Each data point represents a pool of 10 larvae. Unpaired two tailed *t*-test with Welch's correction, ns-not significant. **(D)** Plot showing blood glucose levels of one month old WT and *Tg(ins:p35)* animals. Error bars are mean ± SD from n=5 independent samples per group. Unpaired two tailed *t*-test with Welch's correction, ns-not significant **(E)** Plot showing blood glucose levels in 6 months old WT and *Tg(ins:p35)* animals after overnight fasting (red) and after 1 hour of feeding (blue). Error bars are mean ± SD from n= at least 8 independent samples per group. Unpaired two tailed *t*-test with Welch's correction, ns-not significant. **(F)** Quantification showing the islet area of 6 months old WT and Tg(ins:p35) animals. Error bars are mean ± SD from n=5 independent samples per group. Unpaired two tailed *t*-test with Welch's correction, ns-not significant, **P=0.0089.

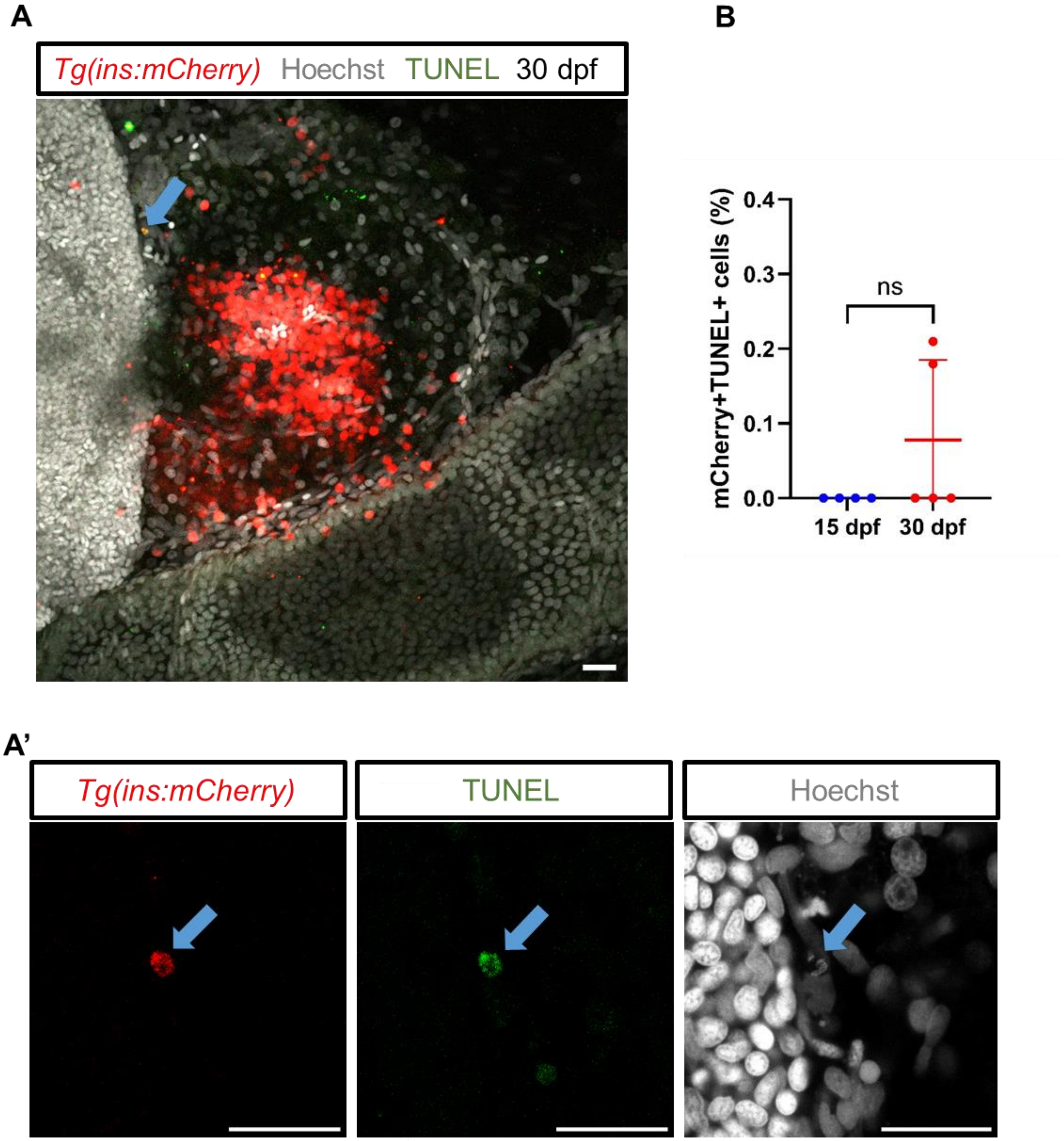

A
Tg(ins:mCherry) Hoechst TUNEL 30 dpf
B
mCherry+TUNEL+ cells (%)
0.4
0.3
0.2
0.1
0.0
ns
15 dpf
30 dpf
A'
Tg(ins:mCherry)
TUNEL
Hoechst

Appendix Figure S3: Histological assessment of beta cell death during islet development. **(A)** Confocal projection of a 30 dpf *Tg(ins:mCherry)* primary islet after TUNEL staining. Scale bar 20 µm. **(A')** Single channel image showing high magnification of the beta cell corresponding to the arrow in figure A. The beta cell is marked in red, Hoechst staining labels the nuclei (grey) while TUNEL positive cells are marked in green. Scale bar 20 µm. (**B**) Quantification showing the percentage of TUNEL+ and mCherry+ cells in the islet of 15 dpf and 30 dpf animals. Error bars are mean ± SD from n=4 independent samples at 15 dpf and 6 independent samples at 30 dpf. Unpaired two tailed *t*-test with Welch's correction. ns – not significant.

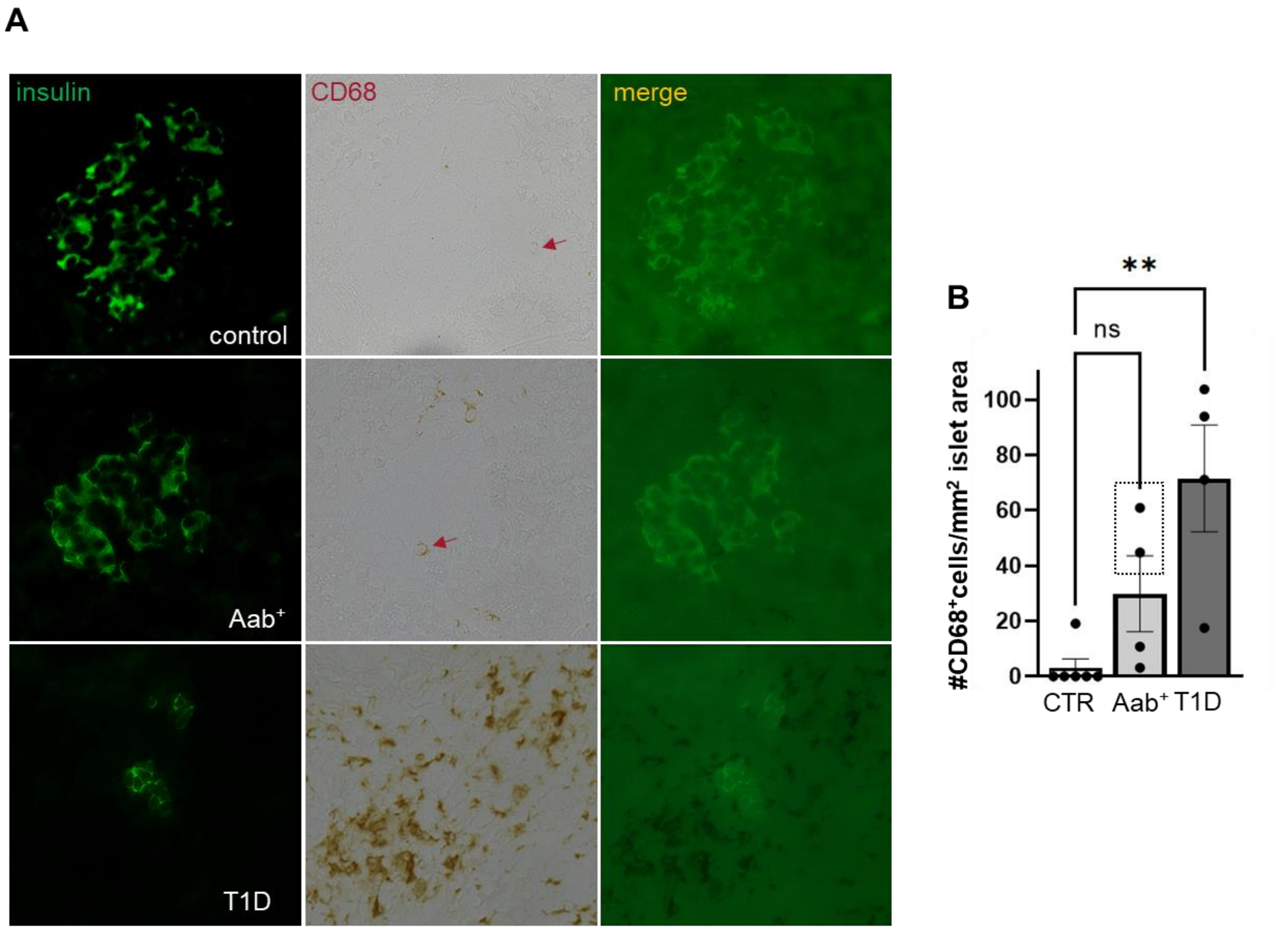
A
insulin
CD68
merge
control
Aab+
T1D
B
**
ns
#CD68+cells/mm2 islet area
100
80
60
40
20
0
CTR
Aab+
T1D

Appendix Figure S4: Islets infiltrating macrophages are increased in organ donors with T1D associated AAbs and in T1D. (A) Representative images of CD68 in brightfield (brown) and insulin (green) in insulin containing islets in FFPE pancreases from autopsy from control donors without diabetes (n=5; donor ID6339), with T1D associated autoantibodies (n=4; donor ID6400) and with T1D (n=4; donor ID6362 & 6371). **(B)** Quantification of the mean number of CD68 positive cells within or around the islet area normalized to mm^2 islet area. The dashed boxes in (B) mark two donors of the study cohort with dual AAb+. Red arrows show CD68+ cells. **p = 0.0029, one-way ANOVA with Dunnett's multiple comparisons test. Stainings were performed in two technical replicas. Error bars are mean ± S.E.M. Scale bar depicts 10µm.

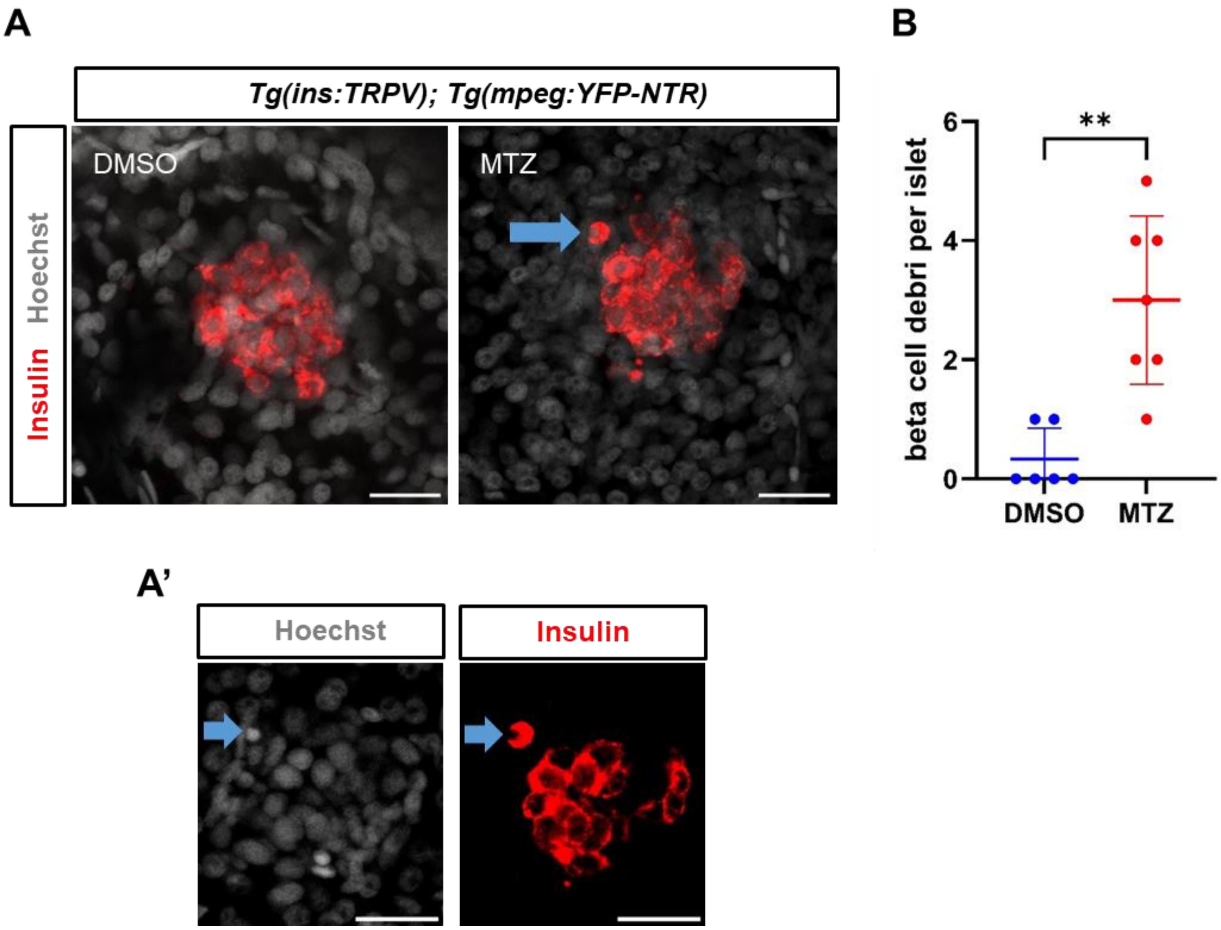
A
Tg(ins:TRPV); Tg(mpeg:YFP-NTR)
Insulin Hoechst
DMSO
MTZ
B
beta cell debri per islet
**
6
4
2
0
DMSO
MTZ
A'
Hoechst
Insulin

Appendix Figure S5: Macrophages play a role in removing apoptotic beta cell corpses from the islet during Ca^{2+} excitotoxicity. **(A)** Representative confocal images (maximum projection) of *Tg(ins:TRPV); Tg(mpeg:YFP-NTR)* larvae after DMSO or MTZ treatment. Both groups were co-treated with csn to activate the TRPV channel. **(A')** Confocal image showing single plane with separate channels from the figure A. Scale bar, 20 µm. **(B)** Quantification showing the number of beta cell debris per islet in the DMSO and MTZ treated larvae. Error bars are mean ± SD from n=at least 6 independent samples per group. Unpaired two tailed *t*-test with Welch's correction, **P=0.0018.

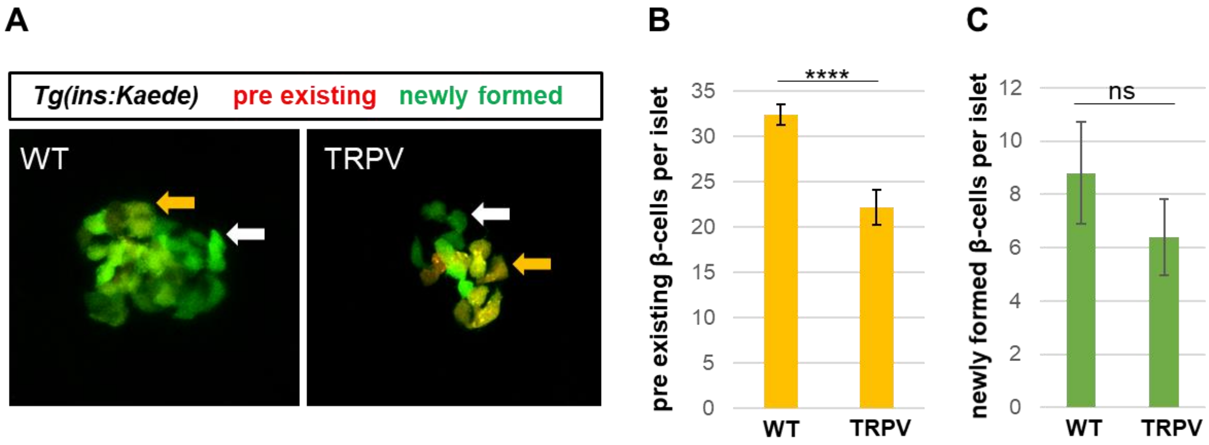
A
Tg(ins:Kaede)
pre existing
newly formed
WT
TRPV
B
pre existing β-cells per islet
35
30
25
20
15
10
5
0
****
WT
TRPV
C
newly formed β-cells per islet
12
10
8
6
4
2
0
ns
WT
TRPV

Appendix Figure S6: Chronic excitotoxicity does not impair differentiation of beta cells.

(A) Representative confocal images (maximum projection) of *Tg(ins:Kaede)* and *Tg(ins:Kaede);Tg(ins:TRPV)* from the experiment in Figure 4H. The yellow arrow points to the pre-existing (RFP+GFP+) beta cells while the white arrow points to the newly formed (GFP+) beta cells. **(B)** Quantification showing the number of pre-existing beta cells per islet in WT and TRPV larvae post csn treatment. Error bars are mean ± SD from n=at least 5 independent samples per group. **(C)** Quantification showing the number of newly formed (GFP+) beta cells per islet in WT and TRPV larvae post csn treatment. Error bars are mean ± SD from n=at least 5 independent samples per group. Unpaired two tailed *t-test* with Welch's correction, ****P= 0.0000000163, ns-not significant.

A

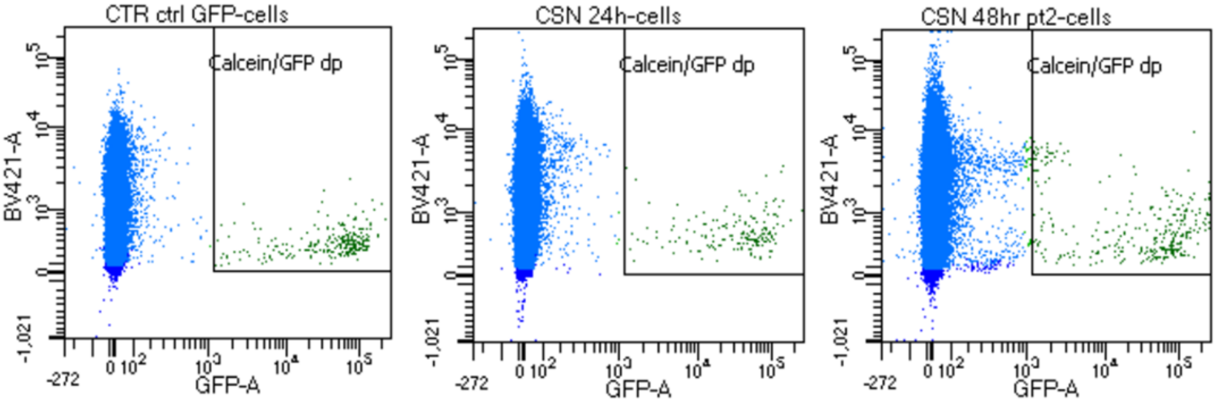

CTR ctrl GFP-cells
CSN 24h-cells
CSN 48hr pt2-cells
Calcein/GFP dp
BV421-A
GFP-A

B

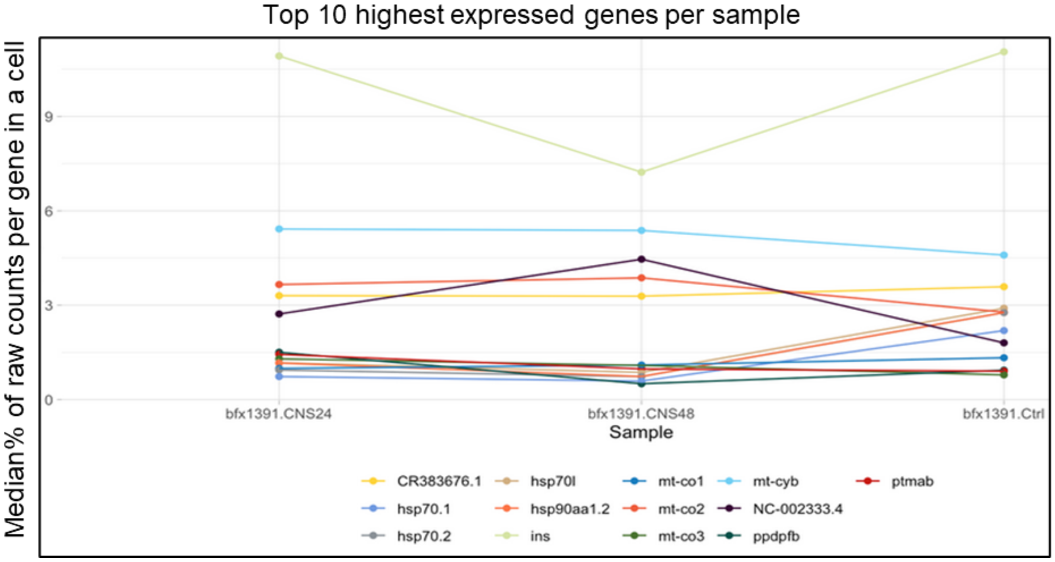

Top 10 highest expressed genes per sample
Median% of raw counts per gene in a cell
Sample
bfx1391.CNS24
bfx1391.CNS48
bfx1391.Ctrl
CR383676.1
hsp70.1
hsp70.2
hsp70l
hsp90aa1.2
ins
mt-co1
mt-co2
mt-co3
mt-cyb
NC-002333.4
ppdpfb
ptmab

Appendix Figure S7: Fluorescence activated cell sorting of viable pancreatic beta cells. **(A)** FACS plot showing GFP positive beta cells isolated from ctr, 24 hr csn and 48 hr csn *Tg(ins:TRPV)* larvae respectively. The plot shows the results from the analysis of n=at least 20 larvae combined per group. Viable beta cells were labelled with calcein. **(B)** Plot showing the top 10 highest expressed genes per condition. In all the three groups, *ins* was the highest expressed gene confirming that the sorted GFP positive cells were beta cells.

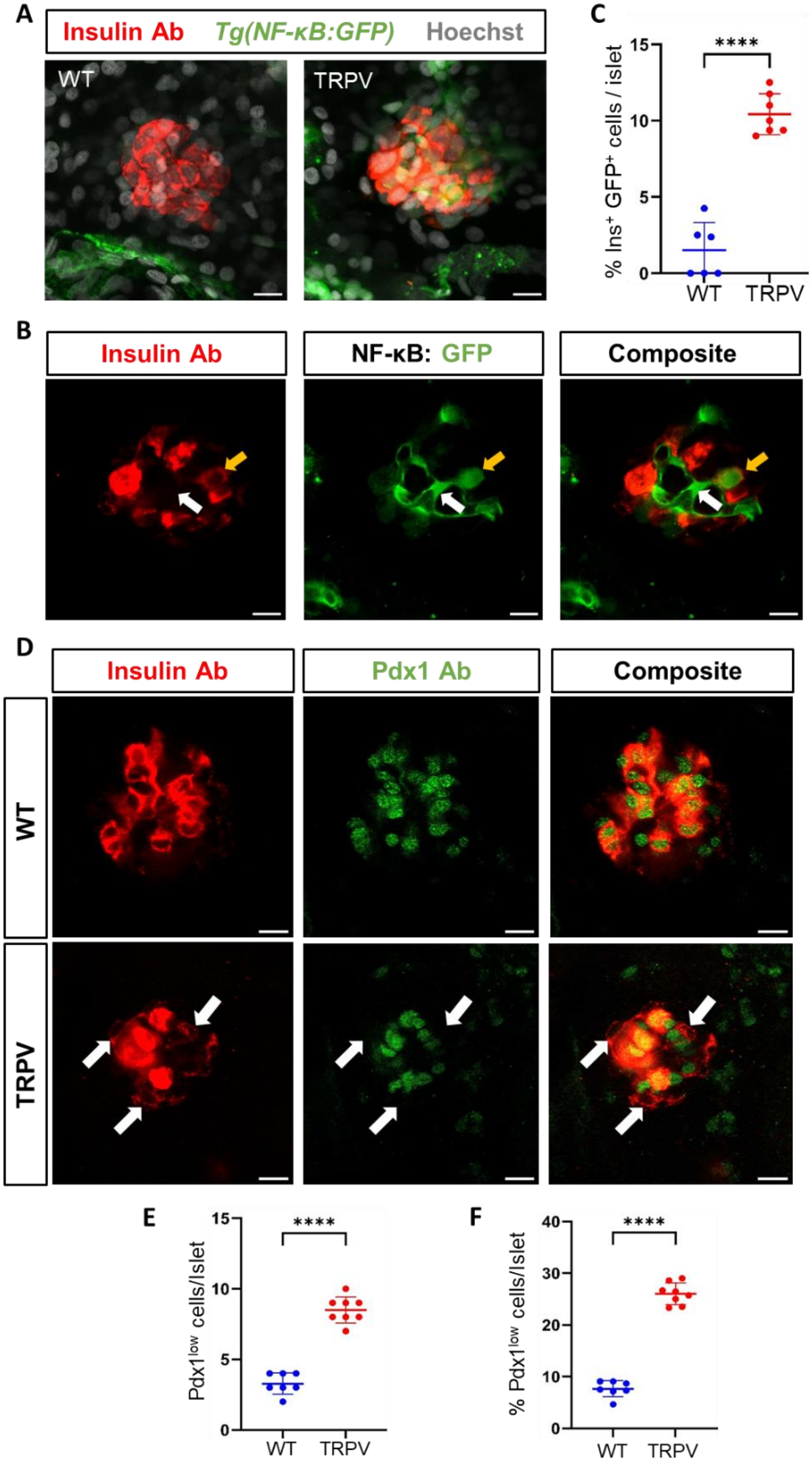
A
Insulin Ab
Tg(NF-κB:GFP)
Hoechst
WT
TRPV
C
****
% Ins⁺ GFP⁺ cells / islet
15
10
5
0
WT
TRPV
B
Insulin Ab
NF-κB: GFP
Composite
D
Insulin Ab
Pdx1 Ab
Composite
WT
TRPV
E
****
Pdx1low cells/Islet
15
10
5
0
WT
TRPV
F
****
% Pdx1low cells/Islet
40
30
20
10
0
WT
TRPV

Appendix Figure S8: Perpetual metabolic stress leads to islet inflammation and loss of key beta cell markers. (A) Confocal images of islets (maximum projection) from WT and *Tg(ins:TRPV)* larvae following csn incubation from 3-5 dpf. The *Tg(NF-kB:GFP)* reporter expresses GFP (green) under the control of six tandem repeats of NF-kB DNA-binding sites. Beta-cells were labelled using an insulin antibody (in red) while the nuclei were marked using Hoechst dye (in grey). (**B)** Single plane from the confocal stack of *Tg(ins:TRPV)* larvae with separate channels. The yellow arrow indicates an insulin positive and GFP positive cell and the white arrow indicates an insulin-negative cell with GFP-expression. Scale bar, 10µm. **(C)** Graph showing the percentage of insulin positive GFP positive beta cells per islet in the WT and *Tg(ins:TRPV)* larvae. Error bars are mean ± SD from n=7 independent samples per group. Unpaired two-tailed *t*-test with Welch's correction, ****P=0.000000818. **(D)** Confocal images of islets (separate channel) from WT and *Tg(ins:TRPV)* larvae following csn incubation from 3-5 dpf. Immunostaining against Insulin labels the beta cells in red and Pdx1 positive cells are shown in green. White arrows indicate beta cells with low Pdx1 expression. Scale bar 10µm. **(E)** Quantification showing the number of beta cells with low Pdx1 expression in WT and *Tg(ins:TRPV)* larvae per islet. Error bars are mean ± SD from n=at least 7 independent samples per group. Unpaired two-tailed *t*-test with Welch's correction, ****P= 0.0000000216. **(F)** Quantification showing the percentage of cells with low Pdx1 expression in WT and *Tg(ins:TRPV)* larvae out of the total number of insulin+ cells per islet. *Tg(ins:TRPV)* larvae show an increase in beta cells with low Pdx1 expression. Error bars are mean ± SD from n=at least 7 independent samples per group. Unpaired two-tailed *t*-test with Welch's correction, ****P=0.0000000000779.

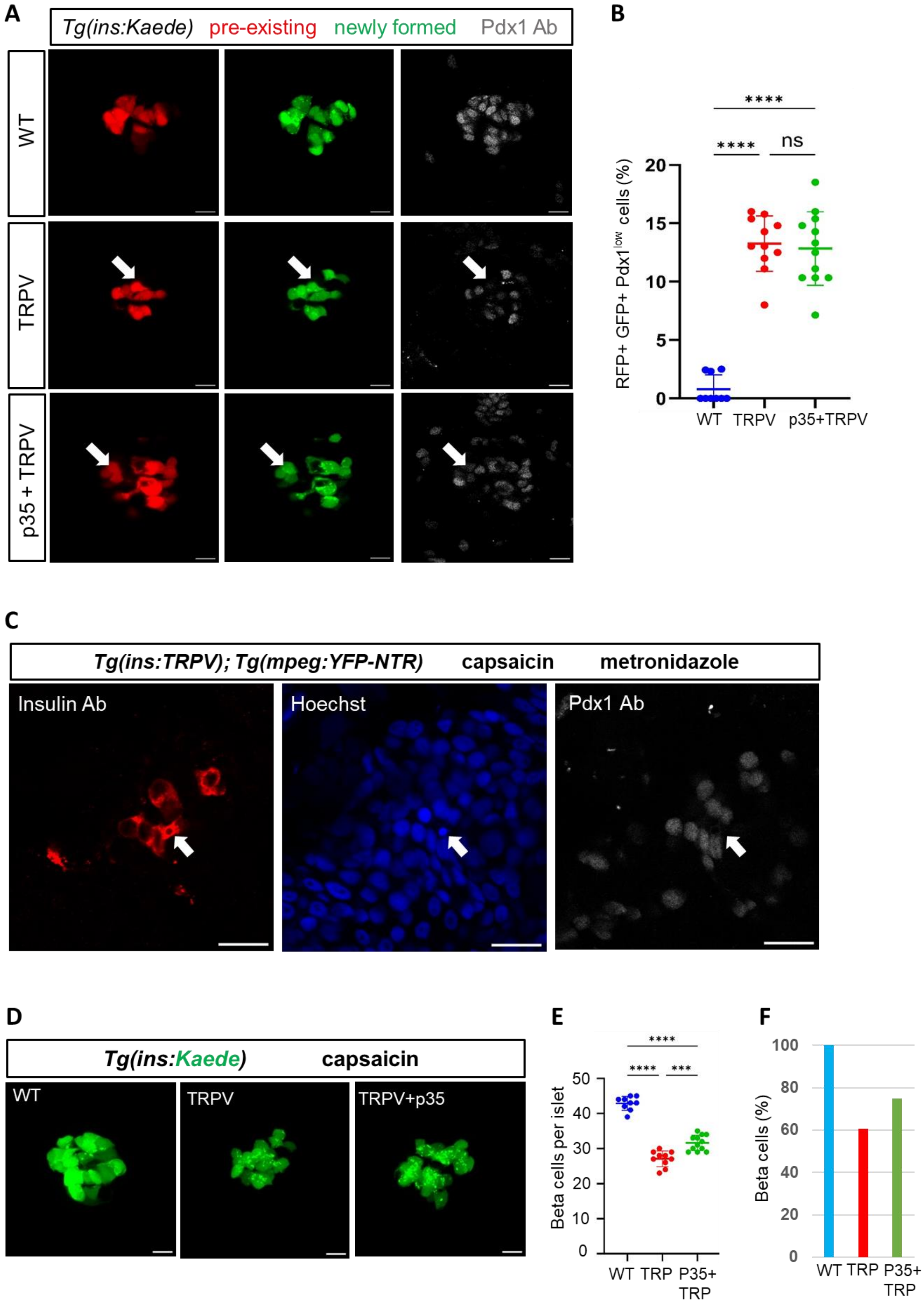
A
Tg(ins:Kaede) pre-existing newly formed Pdx1 Ab
WT
TRPV
p35 + TRPV
B
RFP+ GFP+ Pdx1^low cells (%)
****
****
ns
20
15
10
5
0
WT TRPV p35+TRPV
C
Tg(ins:TRPV); Tg(mpeg:YFP-NTR) capsaicin metronidazole
Insulin Ab
Hoechst
Pdx1 Ab
D
Tg(ins:Kaede) capsaicin
WT
TRPV
TRPV+p35
E
Beta cells per islet
****
****
***
50
40
30
20
10
0
WT TRP P35+ TRP
F
Beta cells (%)
100
80
60
40
20
0
WT TRP P35+ TRP

Appendix Figure S9: Genetic inhibition of endogenous caspases partially rescues beta cells from chronic metabolic stress. (A) Representative confocal images (maximum intensity projection) of islets from WT, *Tg(ins:TRPV)* and *Tg(ins:p35); Tg(ins:TRPV)* larvae in the transgenic background of *Tg(ins:Kaede)* following csn incubation from 3-5 dpf. Beta cells were photo-converted by shining UV light and subsequently the larvae were treated with csn from 3-5 dpf. Pre-existing beta cells express red as well as green fluorescence, newly formed beta cells are marked in green while immunostaining against Pdx1 labels the Pdx1 expressing cells (in grey). White arrow points to pre-existing beta cells with low Pdx1 expression. Scale bar 10µm. **(B)** The graph represents the percentage of beta cells across the three groups Activation of TRPV led to an increase in pre-existing beta cells with low levels of Pdx1. The co-expression of p35 upon TRPV activation did not prevent the reduction of Pdx1 in pre-existing beta cells. Error bars are mean ± SD from n=at least 9 independent samples per group. One-way Anova with Tukey's multiple comparison test, ****P= 0.00000000000174, ns-not significant **(C)** Representative confocal image (single plane) with separate channel of double transgenic *Tg(ins:TRPV);Tg(mpeg:YFP-NTR)* larvae treated with csn and MTZ for 48 hours to activate TRPV while ablating macrophages. Immunostaining against Insulin and Pdx1 labels the beta cells in red and grey, respectively, while the nuclei are stained using Hoechst in blue. The white arrow indicates an apoptotic beta cell that has lost Pdx1 expression. n = 13 cells per islet from 6 independent samples. **(D)** Confocal images (maximum intensity projection) of islets from WT, *Tg(ins:TRPV)* and *Tg(ins:p35); Tg(ins:TRPV)* larvae in the transgenic background of *Tg(ins:Kaede)* following csn incubation from 3-5 dpf. **(E)** Quantification showing the total number of beta cells for each group. Error bars are mean ± SD from n=at least 9 independent samples per group. One-way Anova with Tukey's multiple comparison test, ****P=

0.00000000000000317, ***P=0.000697 **(F)** The data in E expressed as percentages in which the number of cells in WT was set at 100%.

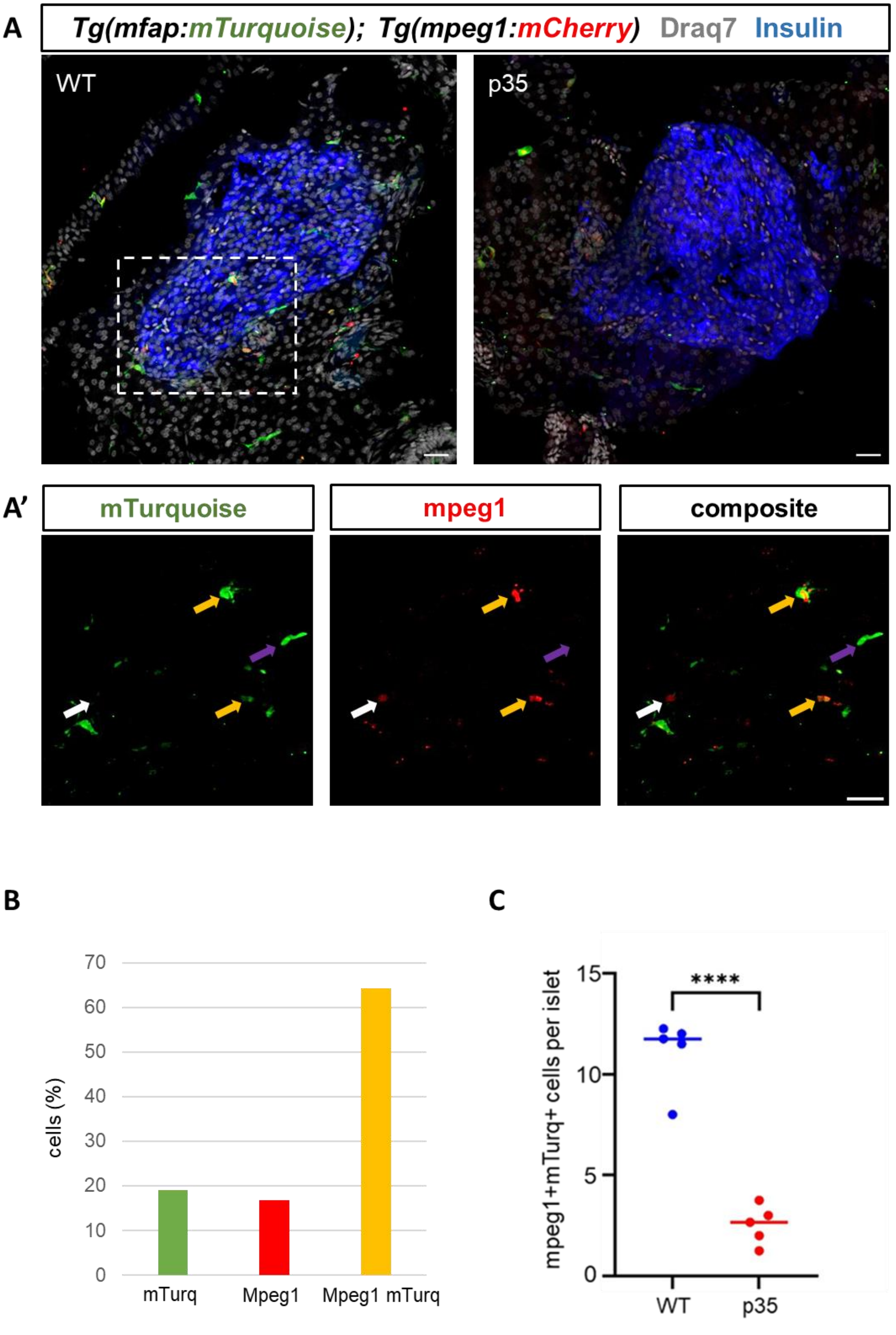

A
Tg(mfap:mTurquoise); Tg(mpeg1:mCherry) Draq7 Insulin
WT
p35
A'
mTurquoise
mpeg1
composite
B
cells (%)
70
60
50
40
30
20
10
0
mTurq
Mpeg1
Mpeg1 mTurq
C
mpeg1+mTurq+ cells per islet
15
10
5
0
****
WT
p35

Appendix Figure S10: *mpeg1* transgenic expression shows co-localized expression with *mfap4* transgenic expression in zebrafish islet. **(A)** Representative confocal images (maximum projection) of islets from 2 months old WT and Tg(ins:p35) animals in the transgenic background of *Tg(mpeg1:mCherry); Tg(mfap4:mTurquoise)*. Immunostaining against insulin marks the beta cells (in blue). The *Tg(mpeg1:mCherry)* line stained with an anti-mCherry antibody marks the mpeg1-positive cells in red, and the *Tg(mfap4:mTurquoise)* line stained with an anti-GFP antibody marks the mfap4 expressing cells in green, while Draq7 dye marks the nuclei in grey. The imaging was performed on tissue sections. **(A')**Insets show high-magnification single plane (separate channel) from the confocal projection of the WT animal (corresponding to the area marked using white dotted-line in A). Yellow arrows point towards mpeg1- and mfap4-double positive cells; white arrows point towards mpeg1-only positive cells, while purple arrows point towards mfap4-only positive cells. Scale bar, 20 µm. **(B)** Percentage of cells with mpeg1 only, mfap4 only and double-positivity. n = 233 cells from 5 animals. **(C)** Quantification showing the total number of mpeg1 and mfap4 double-positive cells per islet in WT and *Tg(ins:p35)*. Error bars are mean ± SD from n= 5 independent samples per group. Unpaired two-tailed t-test with Welch's correction, ****P=0.00000161.

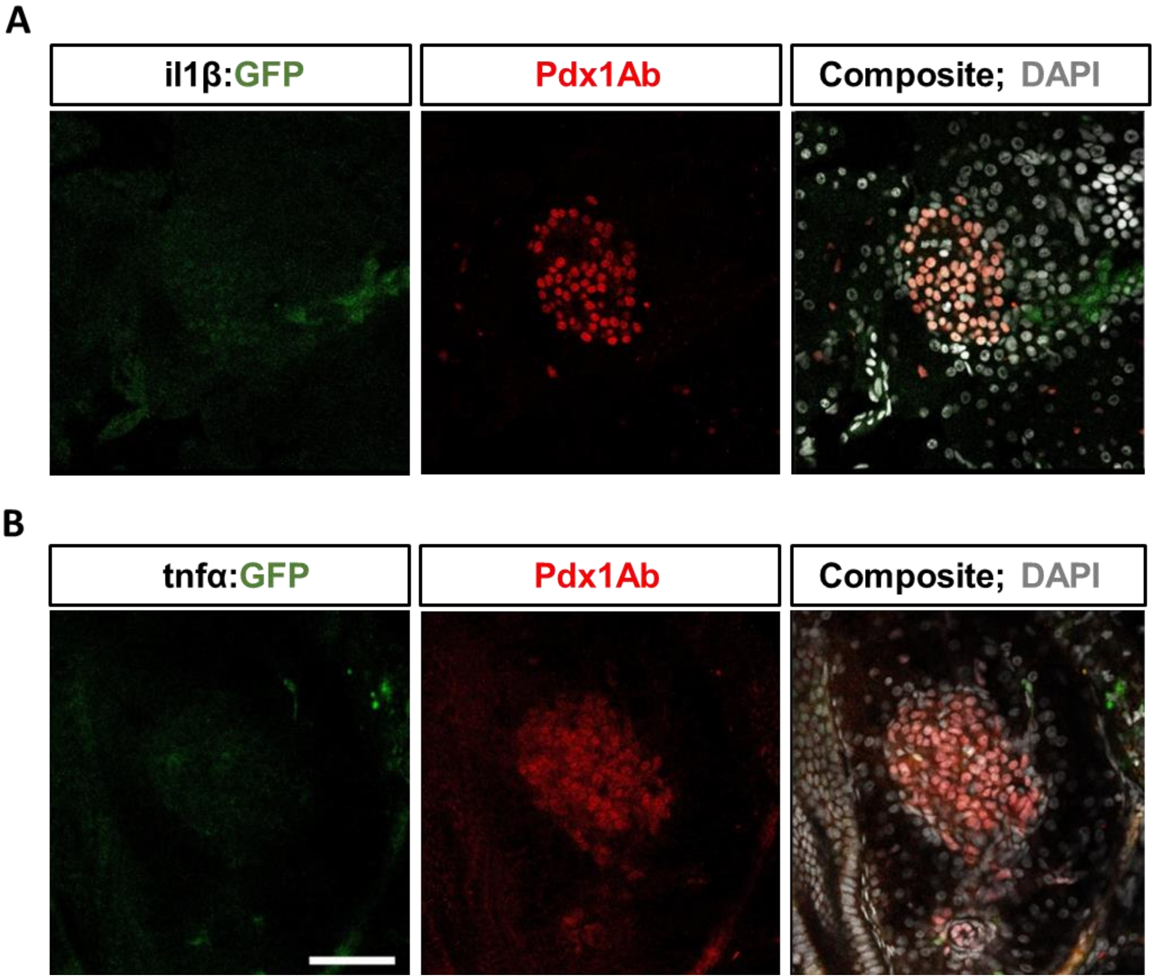
A
il1β:GFP
Pdx1Ab
Composite; DAPI
B
tnfα:GFP
Pdx1Ab
Composite; DAPI

Appendix Figure S11: The majority of beta-cells do not express pro-inflammatory cytokines. Representative confocal images (optical sections) of pancreatic islets from 25 days old juvenile zebrafish. *Tg(il1b:GFP)* marks the cells expressing il1β (panel A) and *Tg(tnfα:GFP)* marks the cells expressing *tnfα* (panel B) in green. Immunostaining against Pdx1 labels the Pdx1 expressing cells in red while the nuclei are stained using DAPI in grey. Scale bar, 50µm.

Appendix Supplementary Materials

Mathematical model of beta cell turnover

Lutz Brusch, Center for Information Services and High Performance Computing, TU Dresden

23 May, 2022

Strategy

The model is here developed through a series of increasingly complex models which stepwise include more mechanisms and flexibility and therefore can fit the data better. From observing how a specific added mechanisms and model flexibility caused a large or small improvement in data fit, we can learn the importance of specific mechanisms. As a start, all models assume fixed parameter values (like the cell cycle duration of proliferating beta cells to be Tc=1d) over the whole observation period of 30d. In a following round, we can add rules for gradual or switch-like changes of such properties. The following sections gradually increase model complexity by adding mechanisms one by one. Each section shows a sketch of the model with the considered mechanisms in black and irgnored processes in grey. For each model, the entire parameter set needs to be optimized independently. Results will be compared for WT (black) and p35 (red) cells to experimental data at three time points 5d, 15d, 30d. The model for p35 cells shares the parameter set with the WT cell model exept for setting the apoptosis rate to zero. Model parameter optimization is performed simultaneously for both cell types, hence fitting 6 data points. The number of fit parameters increases from 2 in model 1 to 8 in model 9, each including the initial condition at time 0d as well. We consider proliferating and quiescent cells with cell numbers Np and Nq, respectively. Model results for the sum N(t) = Np(t) + Nq(t) are compared to the experimental cell number counts. Model 1 is the simplest and worst, models 5b and 7 are intermediate, model 8 is the most complex and best fitting.

1. Beta cell proliferation

Given the experimentally measured cell cycle duration Tc, we first evaluate the resulting cell number dynamics if no other processes would occur. The observed discrepancy between model prediction and experimental data then sets the scale of needed model improvements and allows to judge the scale of the remaining discrepancies in more complex models.

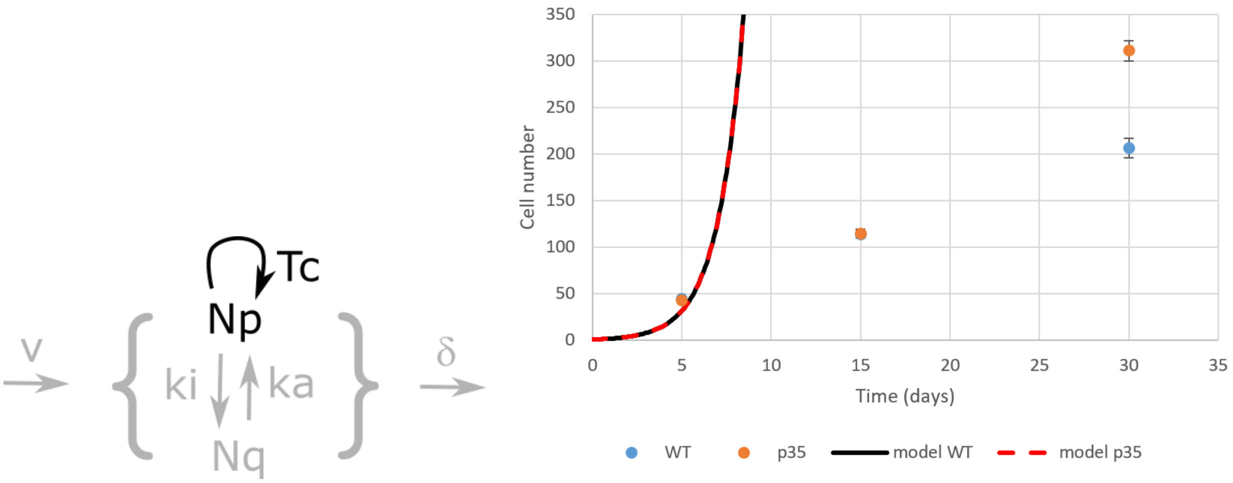

The two fit parameters are the initial conditions Np(t=0) for both experiments (WT, p35) and converged to the given lower bound of 1 cell. The final cell number N(t=30d) = $3.4*10^{10}$ is enormously high and results from the observed cell cycle duration alone. The residual error (RMS) is 2544469. This drastic model misfit motivates the addition of growth-opposing cellular processes like quiescence and apoptosis in the following model extensions.

2. Beta cell proliferation, quiescence

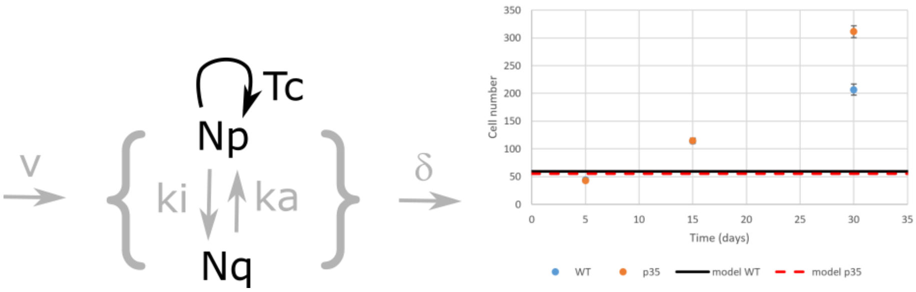

When a fixed quiescent population and the proliferative population (with measured Tc=1d) can start from integer initial cell numbers, then the best fit is achieved by zero proliferative cells and constant cell numbers N(t)=Nq. The residual error (RMS) is 0.560713. This model has 4 fit parameters: the initial cell numbers for Np and Nq separately for each of the two experiments (WT, p35).

3. Beta cell proliferation, apoptosis

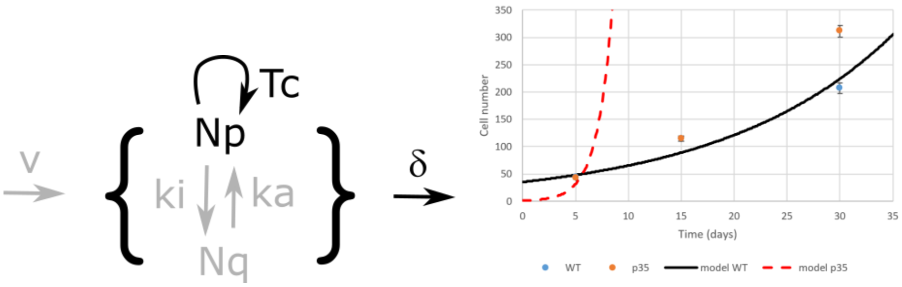

The WT experimental data can be well modelled by proliferation (Tc=1d) and a high rate of apoptosis (63% of cells per day). However, the p35 data cannot be explained that way. The residual error (RMS) is 1408012 (about half the error of model 1 since the p35 data still shows an extreme misfit).

4. Beta cell proliferation, apoptosis, neogenesis

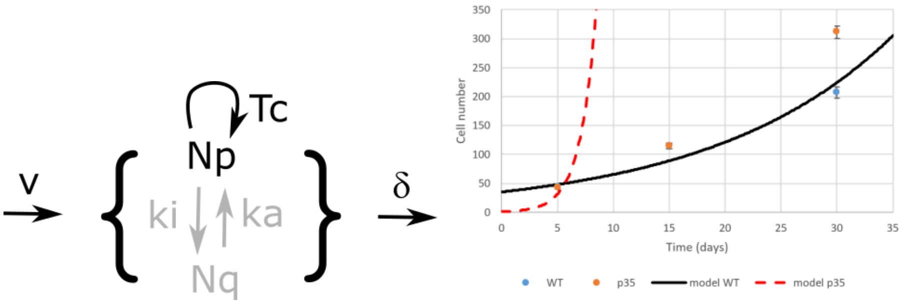

Fit results remain unchanged when neogenesis of beta cells is added to model 3. The fit converges to v=0 and the residual error (RMS) remains at 1408012.

5. Beta cell proliferation, apoptosis, neogenesis, inactivation

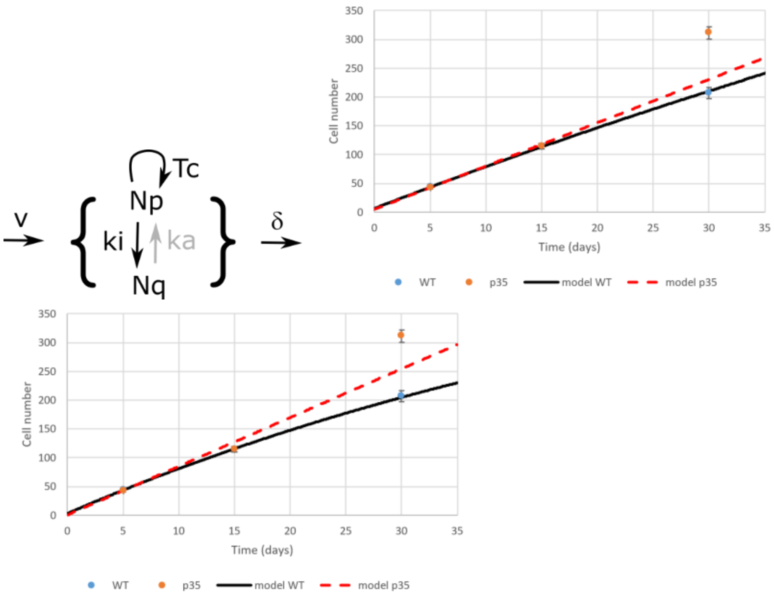

Progressive inactivation of newly formed beta cells improves the fit for the p35 experiment which cannot depend on apoptosis. However, the p35 data are not fitted as well as the WT data. In the first version (middle panel in figure above), model 5a has 5 fit parameters and the initial quiescent population was set to zero in both experiments, i.e. Nq(t=0). The residual error (RMS) is around 0.1, a fifth of model 2's. In the second version (right panel in figure above), model 5b has 7 fit parameters incl. Nq(t=0) separately in both experiments. Each experiment is fitted slightly better but devistions to p35 data remain. The residual error (RMS) is 0.088360.

6. Beta cell proliferation, apoptosis, neogenesis, activation

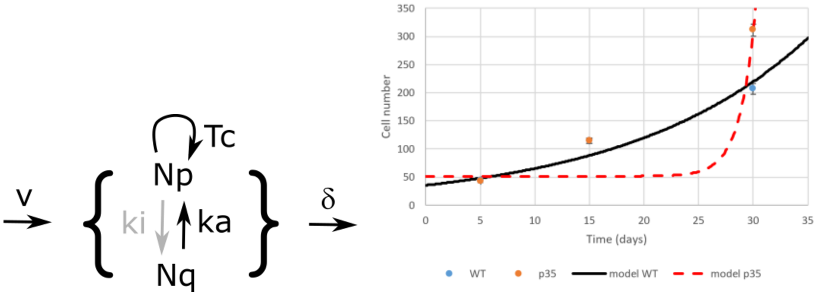

This model 6 benefits from a quiescent cell population but the fit reduced ka to (near) zero. The residual error (RMS) is 0.261089, about half that of model 2.

7. Beta cell proliferation, apoptosis, inactivation, activation

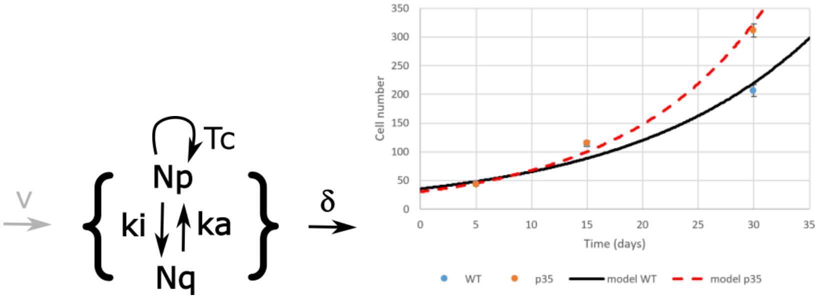

Exchange between proliferating and quiescent cell populations allows for a qualitatively correct fit of both experimental data sets with small remaining deviations. The residual error (RMS) is 0.119491, comparable to model 5a.

8. Beta cell proliferation, neogenesis, inactivation, activation

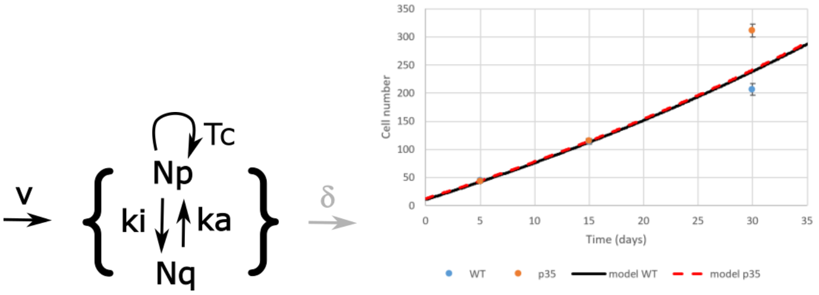

Exchange between proliferating and quiescent cell populations allows for a qualitatively correct fit of both experimental data sets with small remaining deviations in the absence of apoptosis. Without apoptosis, both models possess the same processes and identical parameters (except the initial conditions). The residual error (RMS) is 0.113823, comparable to model 5a.

9. Beta cell proliferation, apoptosis, neogenesis, inactivation, activation

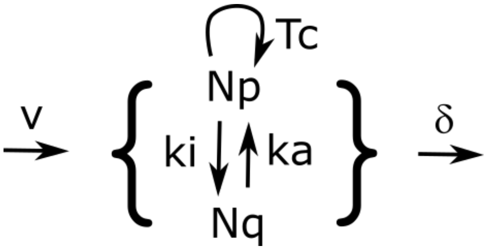

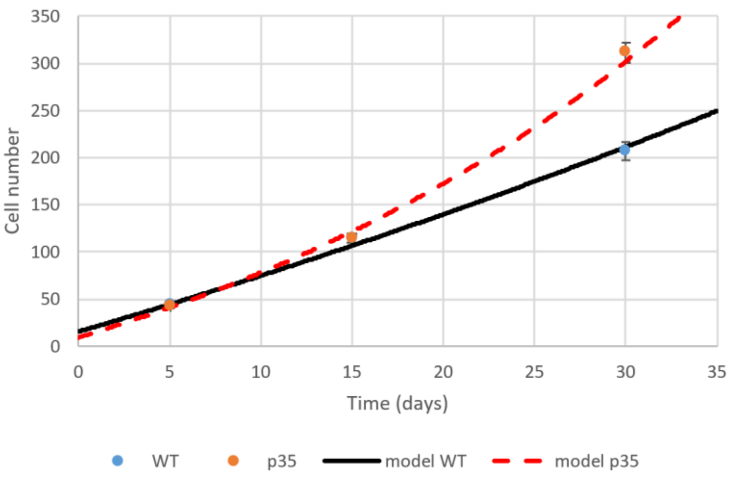

Finally, considering all proposed cellular processes together, both experimental data sets can be modelled well. The residual error (RMS) is as low as 0.041565, about half that of model 5b. This final model has 8 fit parameters as show in the following table.

Parameter	*Unit*	*Value fitted for WT*	*Value fitted for p53*
Cell cycle duration Tc	d	1	set to same as WT
Neogenesis flux v	number of cells/d	5.52	set to same as WT
Apoptosis rate d	% of cells /d	2.25	set to 0
Inactivation rate ki	% of cells /d	7550	set to same as WT
Activation rate ka	% of cells /d	359	set to same as WT
Initial proliferating Np(0)	number of cells	16	10
Initial quiescent Nq(0)	number of cells	0	0

This model fit predicts a fast inactivation process and correspondingly many quiescent cells in both experiments, see figures below. Only 4.5% of WT cells and 4.6% of p35 cells are predicted to be proliferative, the majority would be quiescent.

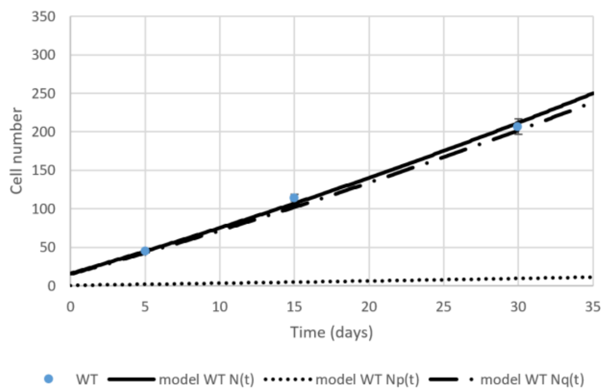

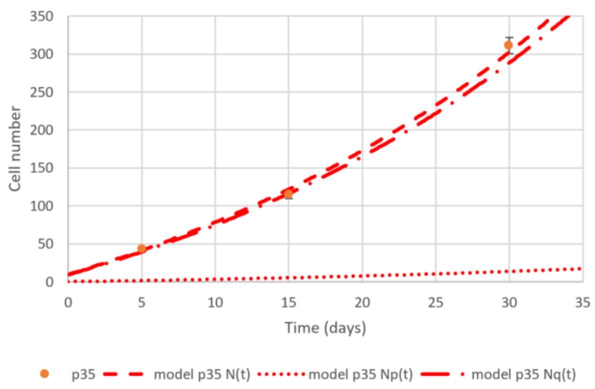

Outlook

(i) Variability between samples from differently grown fish at each time point may be reduced by normalizing to a reference fish size. Then SEM values should be introduced into the fit. However, results may not change much.

(ii) Parameters can be made time-dependend in a continuous or step wise manner, e.g. to modulate cell cycle duration Tc. Then we would need measurements of Tc at different time points.

(iii) Can the fraction of proliferating versus quiescent cells be measured experimentally? That would strongly constrain the inactivation process and other parameter values may need to be readjusted and this may then call for time-dependend rates.

Methods

The mathematical model is formulated as coupled nonlinear ordinary differential equations for Np(t) and Nq(t). Note, the parameter names in the equations do not yet match those in above figures and the table.

$$
\begin{aligned}
\frac{d([Np]\cdot V_V)}{dt} &= +V_V\cdot(kp\cdot[Np]) \\
&\quad -V_V\cdot(k1_{(inactivation)}\cdot[Np]) \\
&\quad +V_V\cdot(k1_{(activation)}\cdot[Nq]) \\
&\quad +V_V\cdot\left(\frac{v\cdot[Np]}{[Np]+[Nq]}\right) \\
&\quad -V_V\cdot(delta\cdot[Np])
\end{aligned}
$$

$$
\begin{aligned}
\frac{d([Nq]\cdot V_V)}{dt} &= +V_V\cdot(k1_{(inactivation)}\cdot[Np]) \\
&\quad -V_V\cdot(k1_{(activation)}\cdot[Nq]) \\
&\quad +V_V\cdot\left(\frac{v\cdot[Nq]}{[Nq]+[Np]}\right) \\
&\quad -V_V\cdot(delta\cdot[Nq])
\end{aligned}
$$

$$N = [Np]+[Nq]$$

$$kp = \frac{0.69314718}{Values[Tc].InitialValue}$$
