# Supplementary material for: Developmental beta-cell death orchestrates the islet’s inflammatory milieu by regulating immune system crosstalk
Source: EMBO J. 2025 Jan 6;44(4):1131–53. doi: 10.1038/s44318-024-00332-w (PMC11833124; doi:10.1038/s44318-024-00332-w)
Supplement: Supplementary file 5 — Movie EV1 [file 44318_2024_332_MOESM5_ESM.zip › Movie EV1/Movie EV1.docx]

**Movie EV1:** Model simulations of islet growth for WT (left) and p35 (right) conditions at synced time from 5-30 dpf as given at bottom. The quiescent cells are marked in red, cycling cells in green while apoptotic cells in blue.
