# Supplementary material for: Developmental beta-cell death orchestrates the islet’s inflammatory milieu by regulating immune system crosstalk
Source: EMBO J. 2025 Jan 6;44(4):1131–53. doi: 10.1038/s44318-024-00332-w (PMC11833124; doi:10.1038/s44318-024-00332-w)
Supplement: Supplementary file 6 — Movie EV2 [file 44318_2024_332_MOESM6_ESM.zip › Movie EV2/Movie EV2.docx]

**Movie EV2:** GCaMP imaging in control fish upon addition of csn. The time-stamp shows 10s per frame.
