# Supplementary material for: Developmental beta-cell death orchestrates the islet’s inflammatory milieu by regulating immune system crosstalk
Source: EMBO J. 2025 Jan 6;44(4):1131–53. doi: 10.1038/s44318-024-00332-w (PMC11833124; doi:10.1038/s44318-024-00332-w)
Supplement: Supplementary file 7 — Movie EV3 [file 44318_2024_332_MOESM7_ESM.zip › Movie EV3/Movie EV3.docx]

**Movie EV3:** GCaMP imaging in *Tg(ins:TRPV)* fish upon addition of csn. The time-stamp shows 10s per frame.
