# Supplementary figures and images for: Developmental beta-cell death orchestrates the islet’s inflammatory milieu by regulating immune system crosstalk (part 1 of 2)
Source: EMBO J. 2025 Jan 6;44(4):1131–53. doi: 10.1038/s44318-024-00332-w (PMC11833124; doi:10.1038/s44318-024-00332-w)

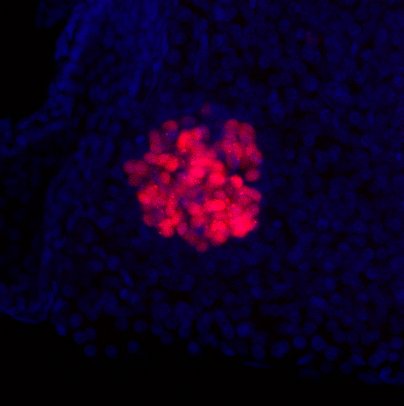

Supplement: Supplementary file 8 — Source data Fig. 1 [file 44318_2024_332_MOESM8_ESM.zip › Figure 1/1C/max proj/p35 15 dpf composite.tif]

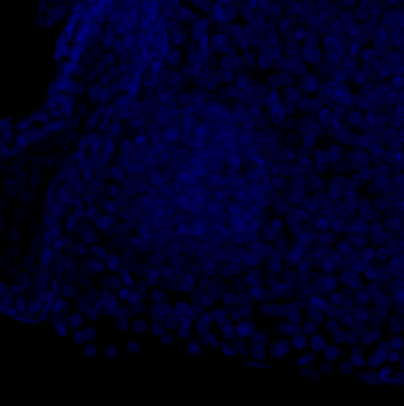

Supplement: Supplementary file 8 — Source data Fig. 1 [file 44318_2024_332_MOESM8_ESM.zip › Figure 1/1C/max proj/p35 15 dpf Hoechst.tif]

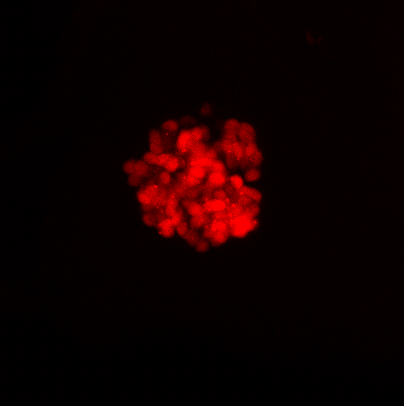

Supplement: Supplementary file 8 — Source data Fig. 1 [file 44318_2024_332_MOESM8_ESM.zip › Figure 1/1C/max proj/p35 15 dpf mCherry.tif]

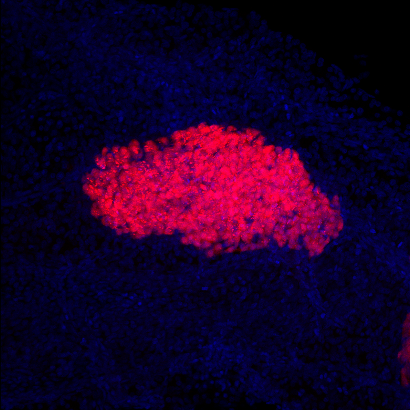

Supplement: Supplementary file 8 — Source data Fig. 1 [file 44318_2024_332_MOESM8_ESM.zip › Figure 1/1C/max proj/p35 30 dpf composite.tif]

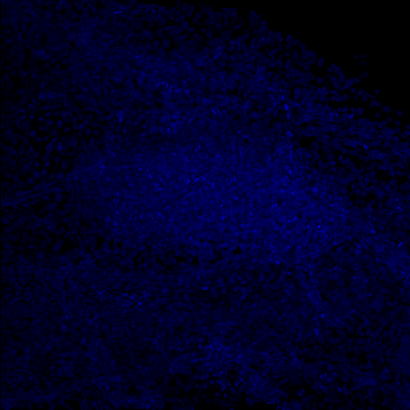

Supplement: Supplementary file 8 — Source data Fig. 1 [file 44318_2024_332_MOESM8_ESM.zip › Figure 1/1C/max proj/p35 30 dpf Hoechst.tif]

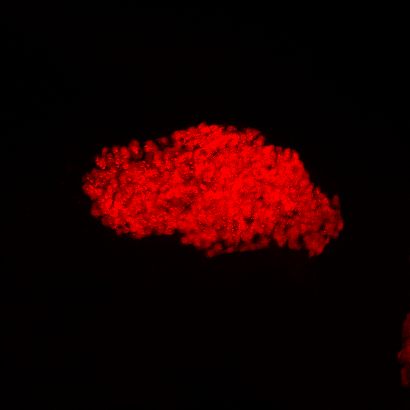

Supplement: Supplementary file 8 — Source data Fig. 1 [file 44318_2024_332_MOESM8_ESM.zip › Figure 1/1C/max proj/p35 30 dpf mCherry.tif]

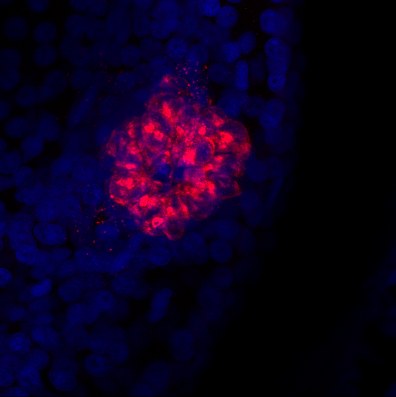

Supplement: Supplementary file 8 — Source data Fig. 1 [file 44318_2024_332_MOESM8_ESM.zip › Figure 1/1C/max proj/p35 5 dpf composite.tif]

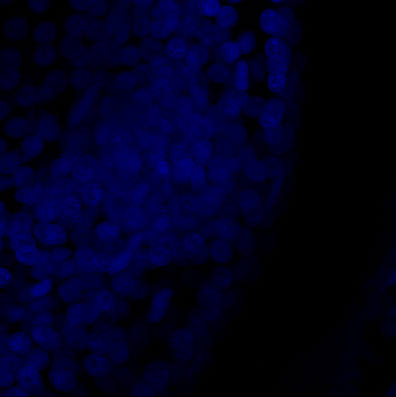

Supplement: Supplementary file 8 — Source data Fig. 1 [file 44318_2024_332_MOESM8_ESM.zip › Figure 1/1C/max proj/p35 5 dpf Hoechst.tif]

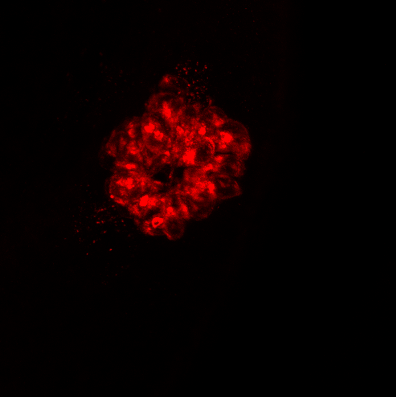

Supplement: Supplementary file 8 — Source data Fig. 1 [file 44318_2024_332_MOESM8_ESM.zip › Figure 1/1C/max proj/p35 5 dpf Ins.tif]

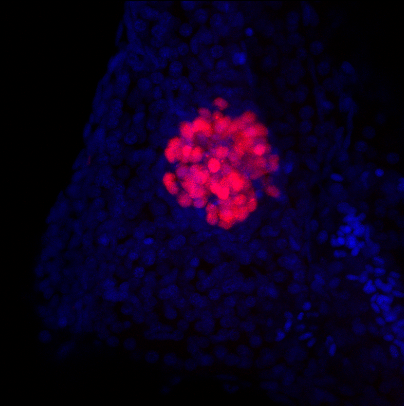

Supplement: Supplementary file 8 — Source data Fig. 1 [file 44318_2024_332_MOESM8_ESM.zip › Figure 1/1C/max proj/WT 15 dpf composite.tif]

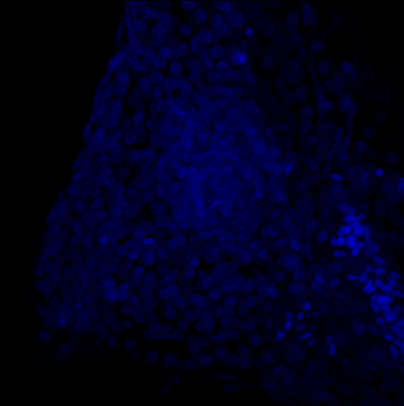

Supplement: Supplementary file 8 — Source data Fig. 1 [file 44318_2024_332_MOESM8_ESM.zip › Figure 1/1C/max proj/WT 15 dpf Hoechst.tif]

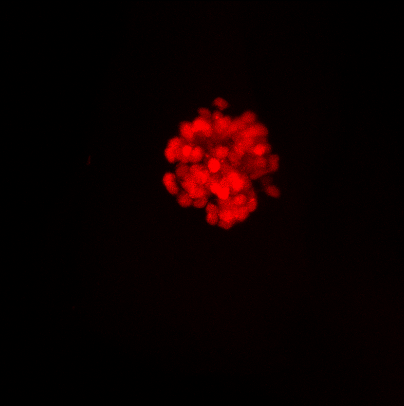

Supplement: Supplementary file 8 — Source data Fig. 1 [file 44318_2024_332_MOESM8_ESM.zip › Figure 1/1C/max proj/WT 15 dpf mCherry.tif]

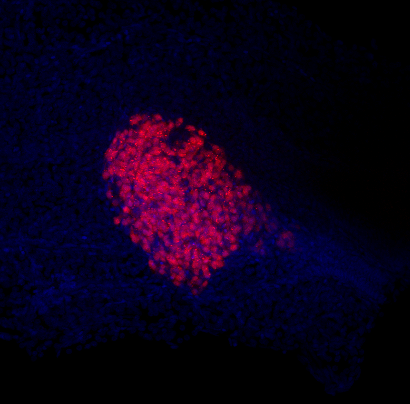

Supplement: Supplementary file 8 — Source data Fig. 1 [file 44318_2024_332_MOESM8_ESM.zip › Figure 1/1C/max proj/WT 30 dpf composite.tif]

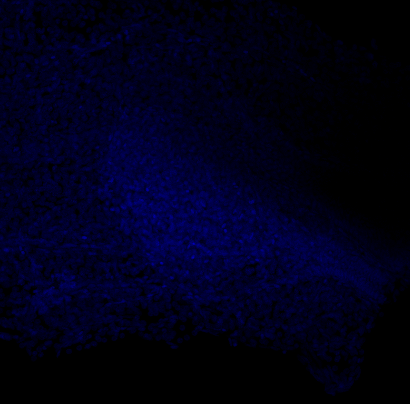

Supplement: Supplementary file 8 — Source data Fig. 1 [file 44318_2024_332_MOESM8_ESM.zip › Figure 1/1C/max proj/WT 30 dpf Hoechst.tif]

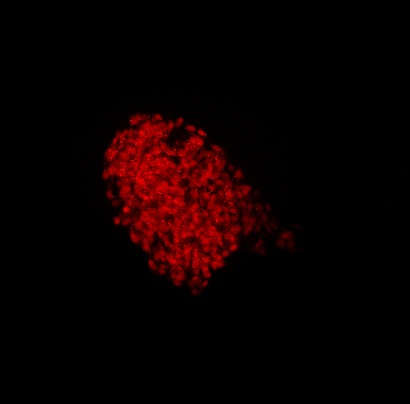

Supplement: Supplementary file 8 — Source data Fig. 1 [file 44318_2024_332_MOESM8_ESM.zip › Figure 1/1C/max proj/WT 30 dpf mCherry.tif]

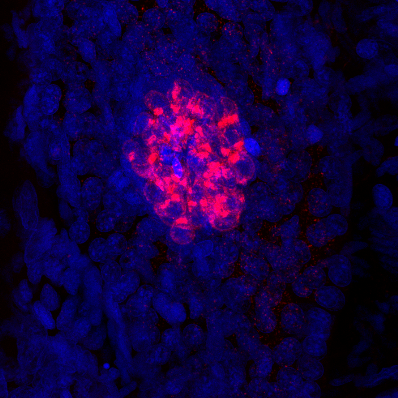

Supplement: Supplementary file 8 — Source data Fig. 1 [file 44318_2024_332_MOESM8_ESM.zip › Figure 1/1C/max proj/WT 5 dpf composite.tif]

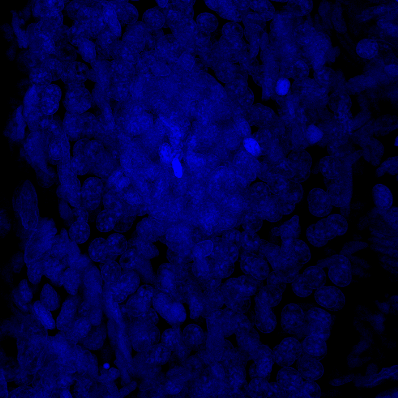

Supplement: Supplementary file 8 — Source data Fig. 1 [file 44318_2024_332_MOESM8_ESM.zip › Figure 1/1C/max proj/WT 5 dpf Hoechst.tif]

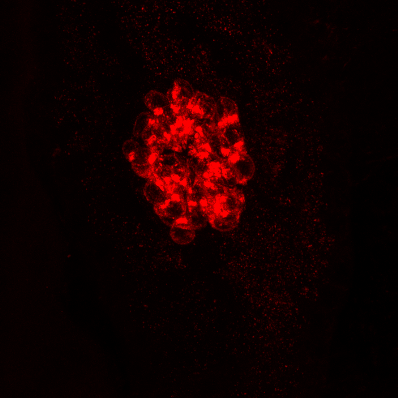

Supplement: Supplementary file 8 — Source data Fig. 1 [file 44318_2024_332_MOESM8_ESM.zip › Figure 1/1C/max proj/WT 5 dpf mCherry.tif]

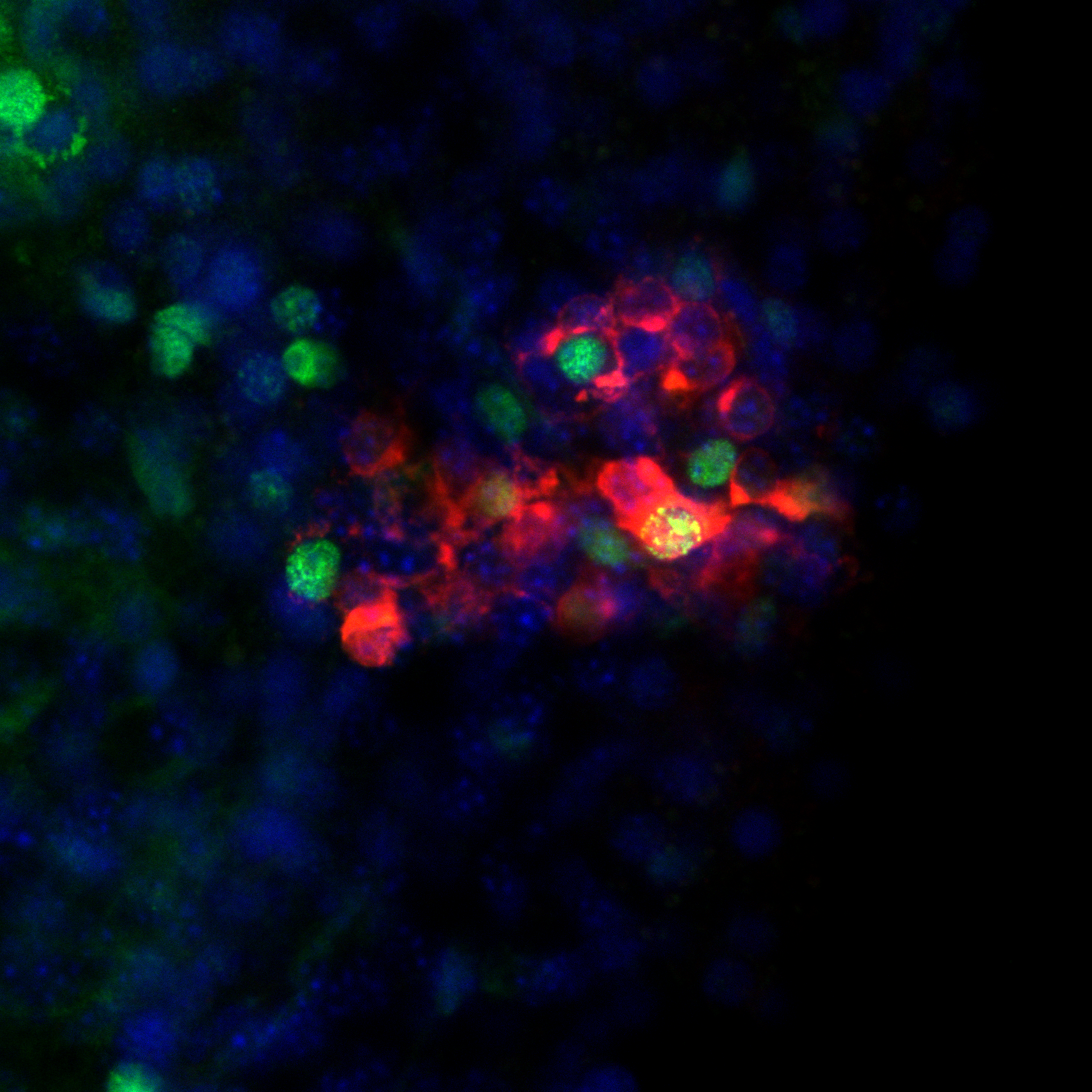

Supplement: Supplementary file 8 — Source data Fig. 1 [file 44318_2024_332_MOESM8_ESM.zip › Figure 1/1D/single plane/p35 15 dpf composite.tif]

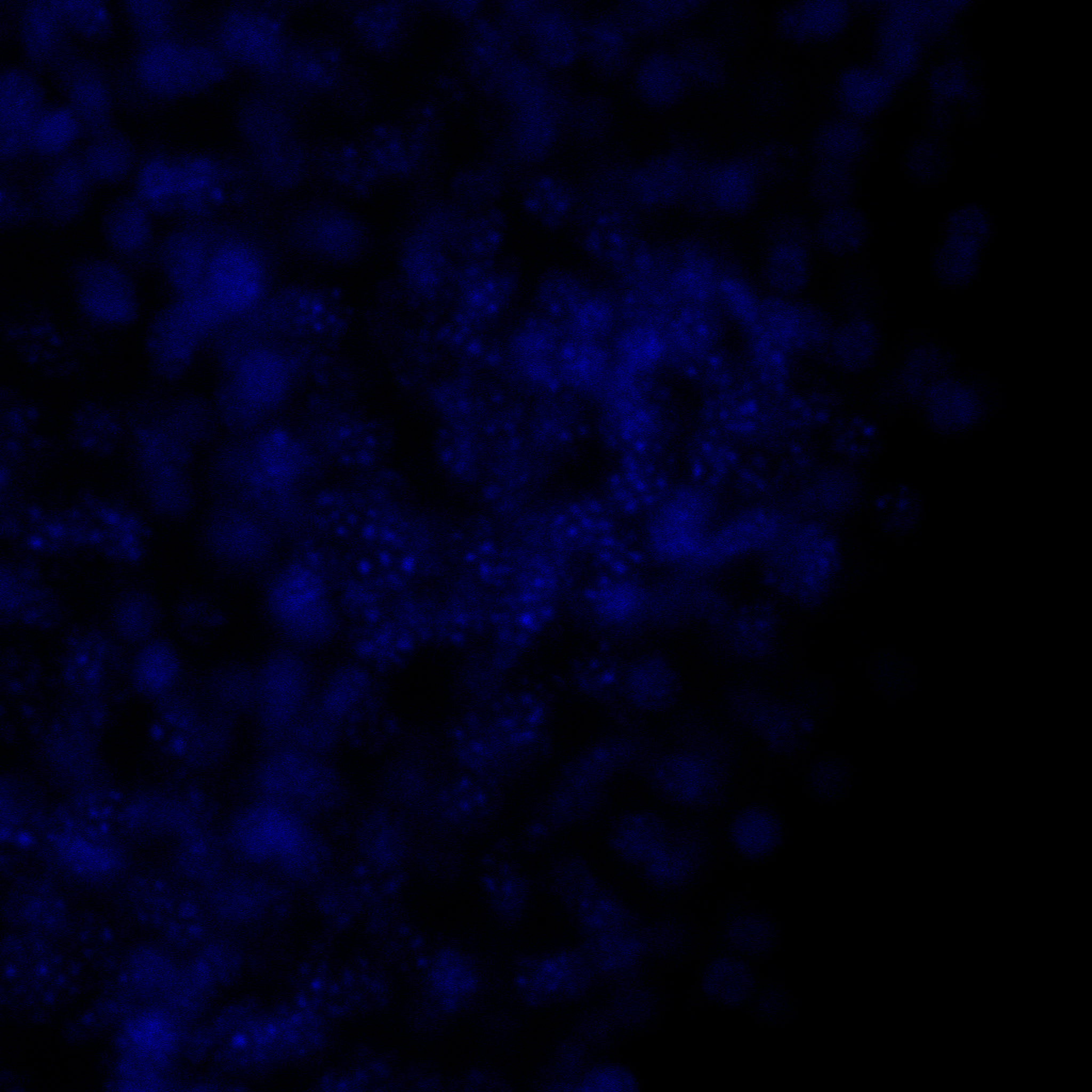

Supplement: Supplementary file 8 — Source data Fig. 1 [file 44318_2024_332_MOESM8_ESM.zip › Figure 1/1D/single plane/p35 15 dpf Hoechst.tif]

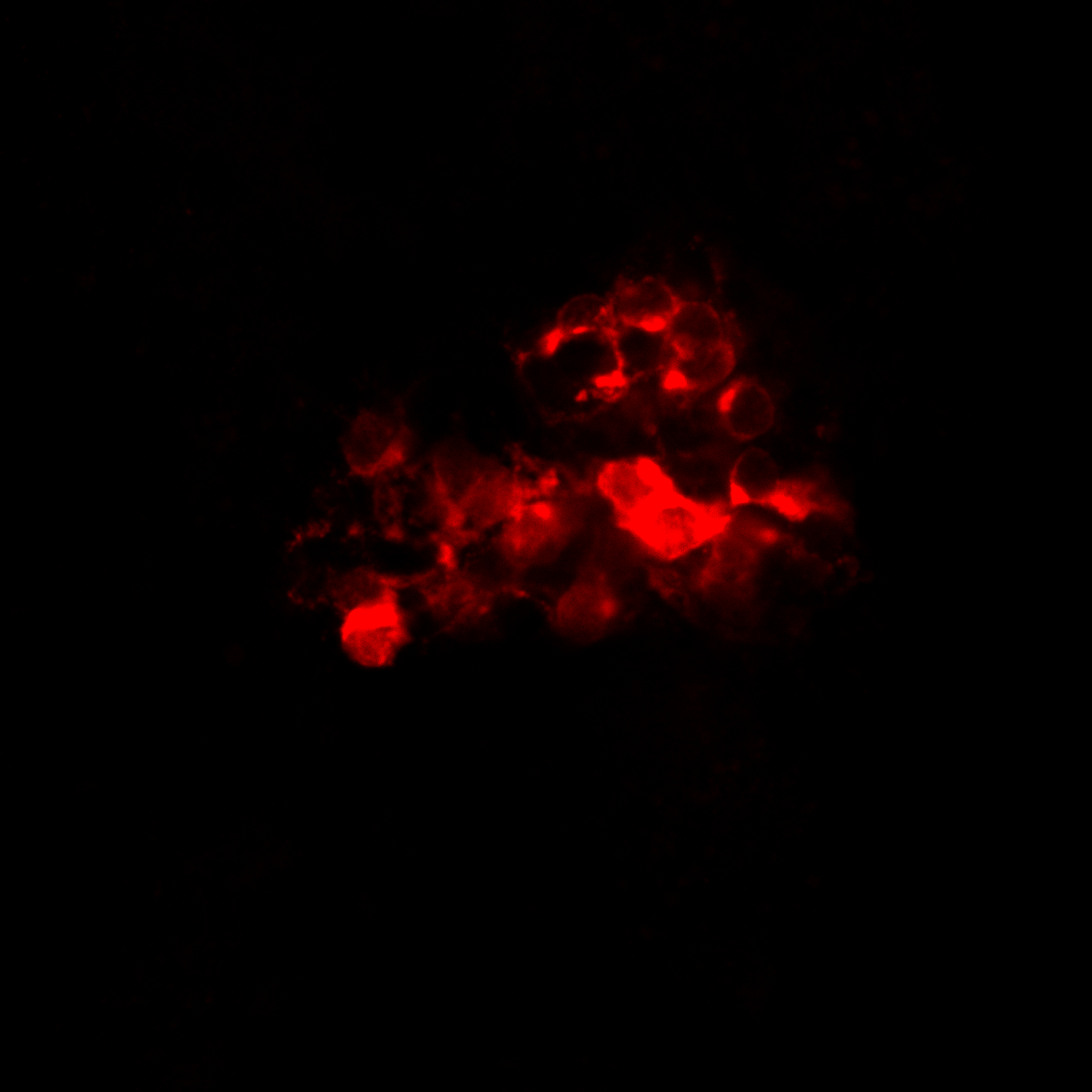

Supplement: Supplementary file 8 — Source data Fig. 1 [file 44318_2024_332_MOESM8_ESM.zip › Figure 1/1D/single plane/p35 15 dpf Ins.tif]

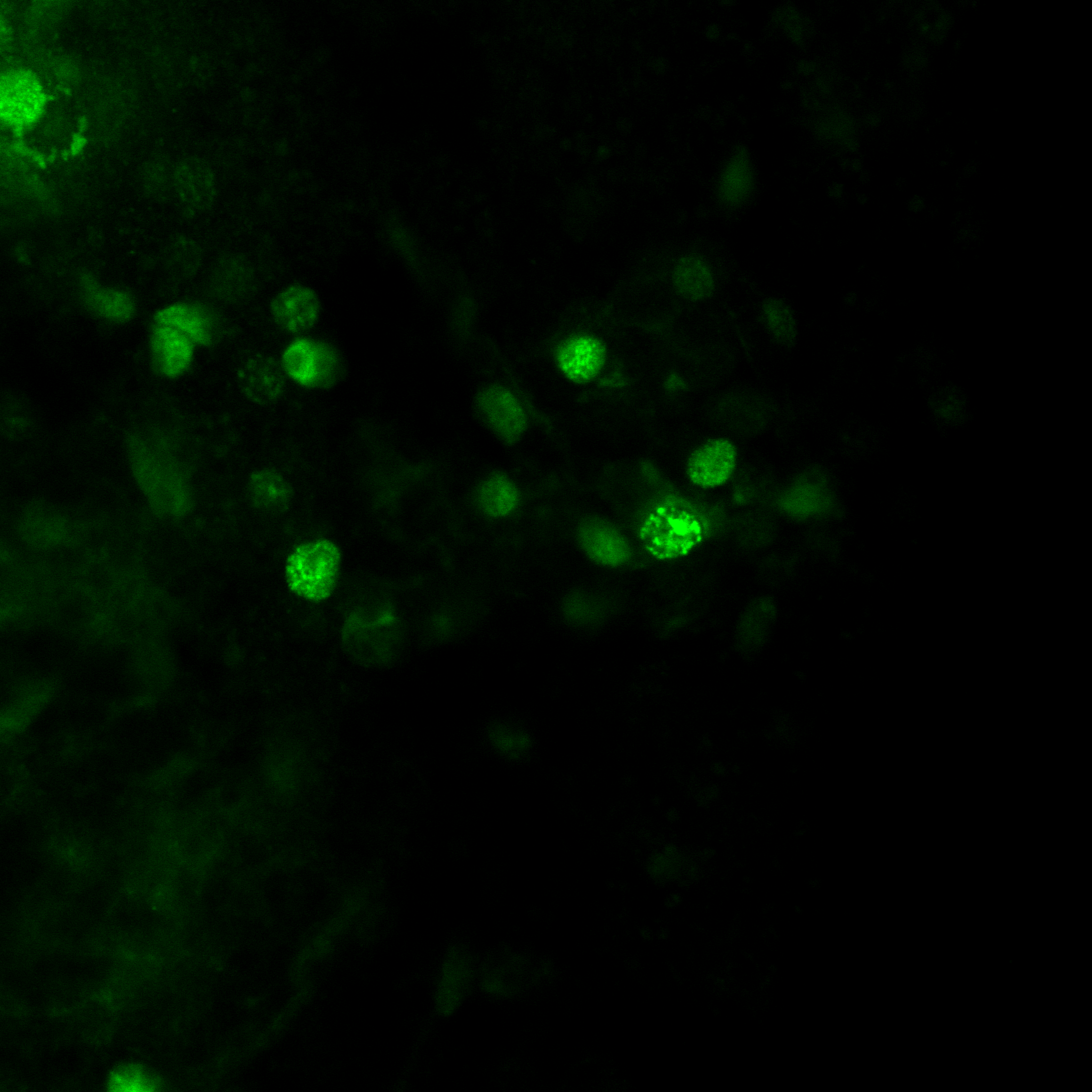

Supplement: Supplementary file 8 — Source data Fig. 1 [file 44318_2024_332_MOESM8_ESM.zip › Figure 1/1D/single plane/p35 15 dpf PCNA.tif]

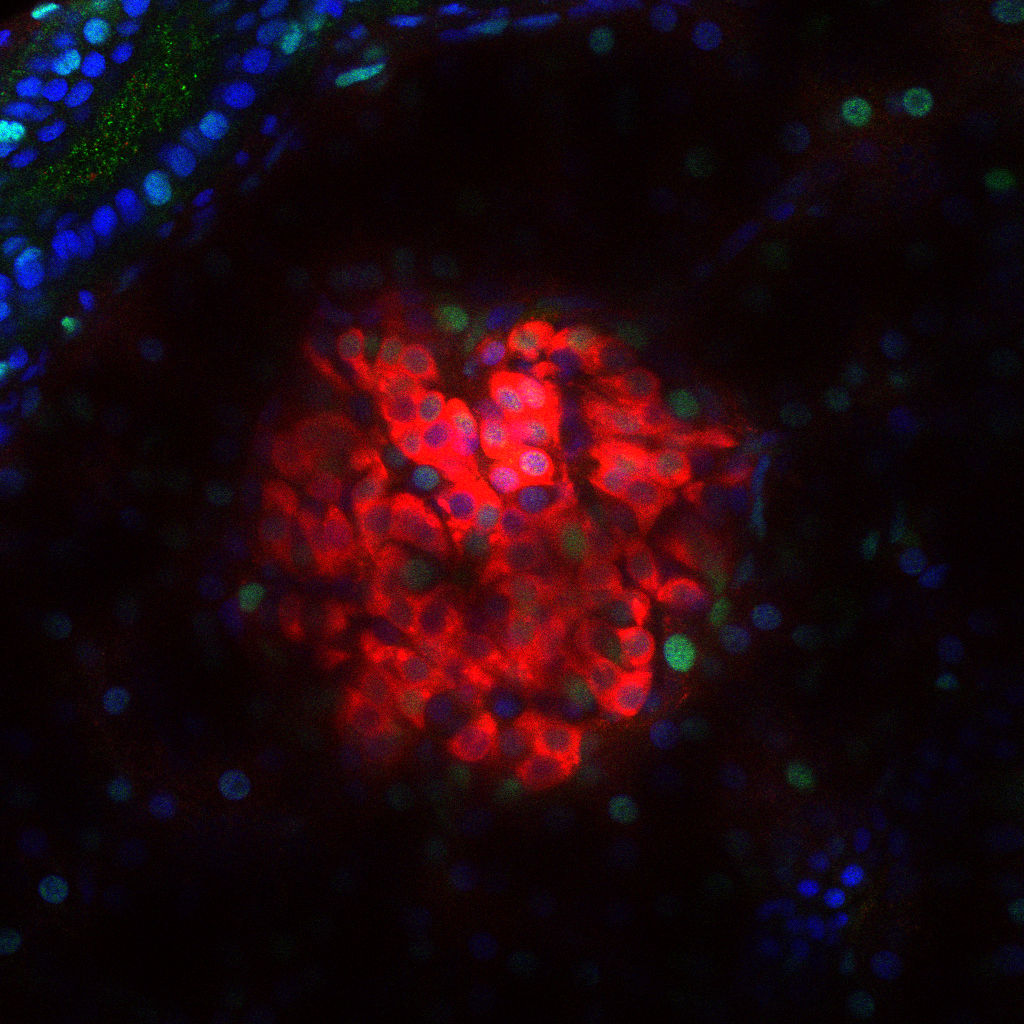

Supplement: Supplementary file 8 — Source data Fig. 1 [file 44318_2024_332_MOESM8_ESM.zip › Figure 1/1D/single plane/p35 25 dpf composite.tif]

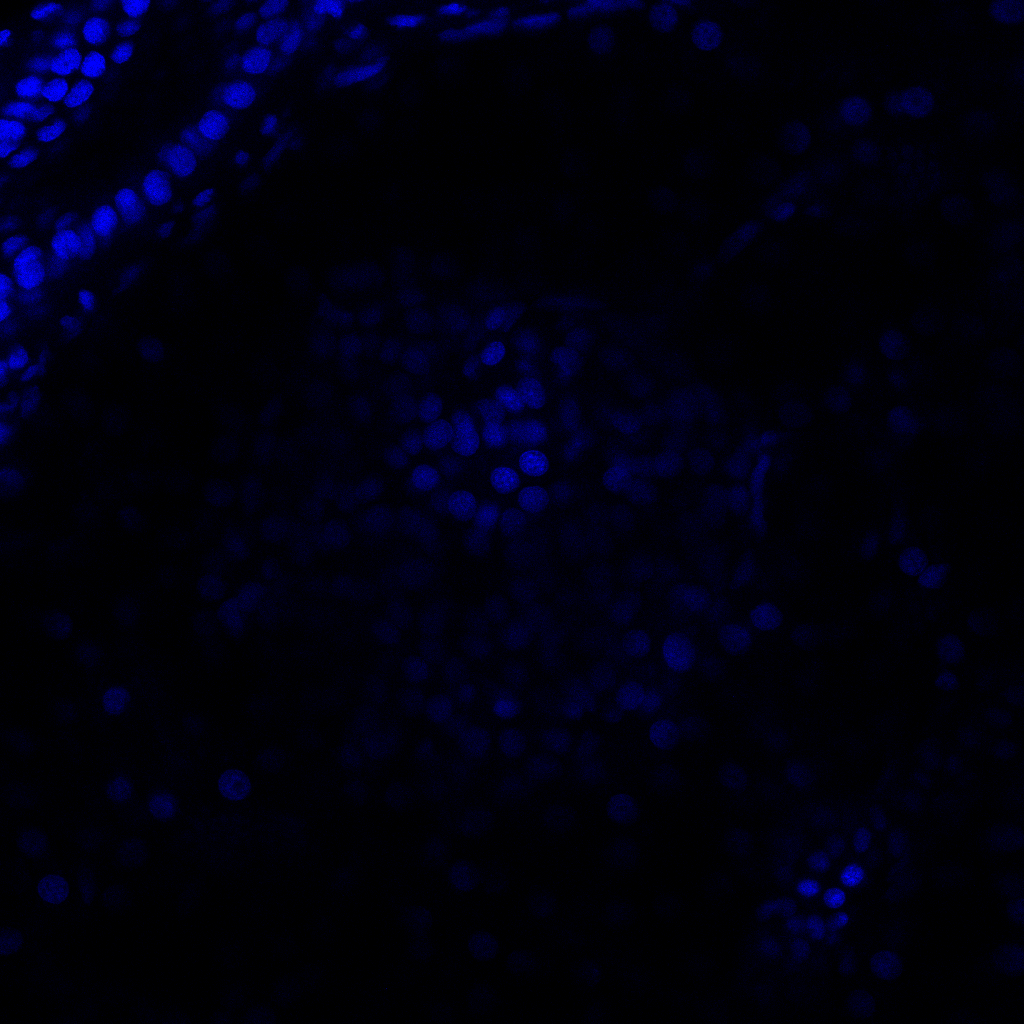

Supplement: Supplementary file 8 — Source data Fig. 1 [file 44318_2024_332_MOESM8_ESM.zip › Figure 1/1D/single plane/p35 25 dpf Hoechst.tif]

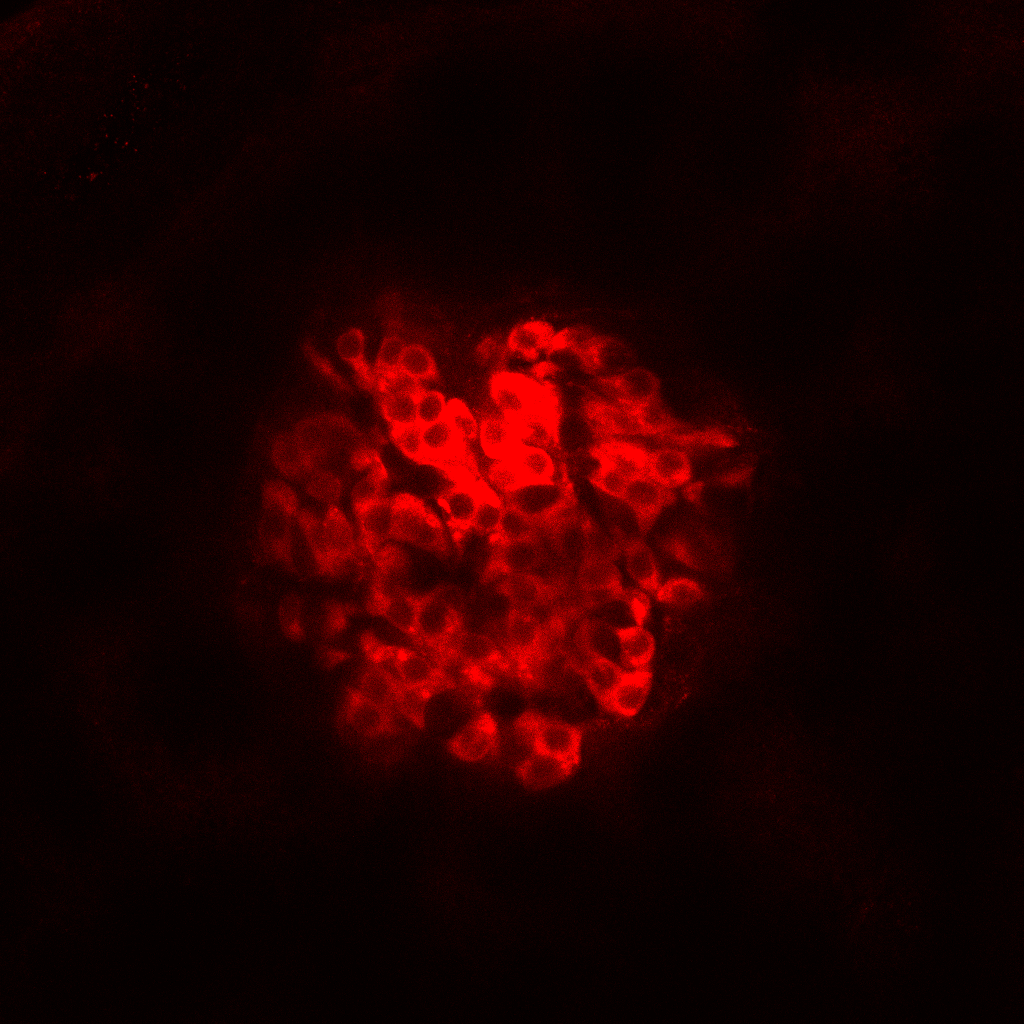

Supplement: Supplementary file 8 — Source data Fig. 1 [file 44318_2024_332_MOESM8_ESM.zip › Figure 1/1D/single plane/p35 25 dpf Ins.tif]

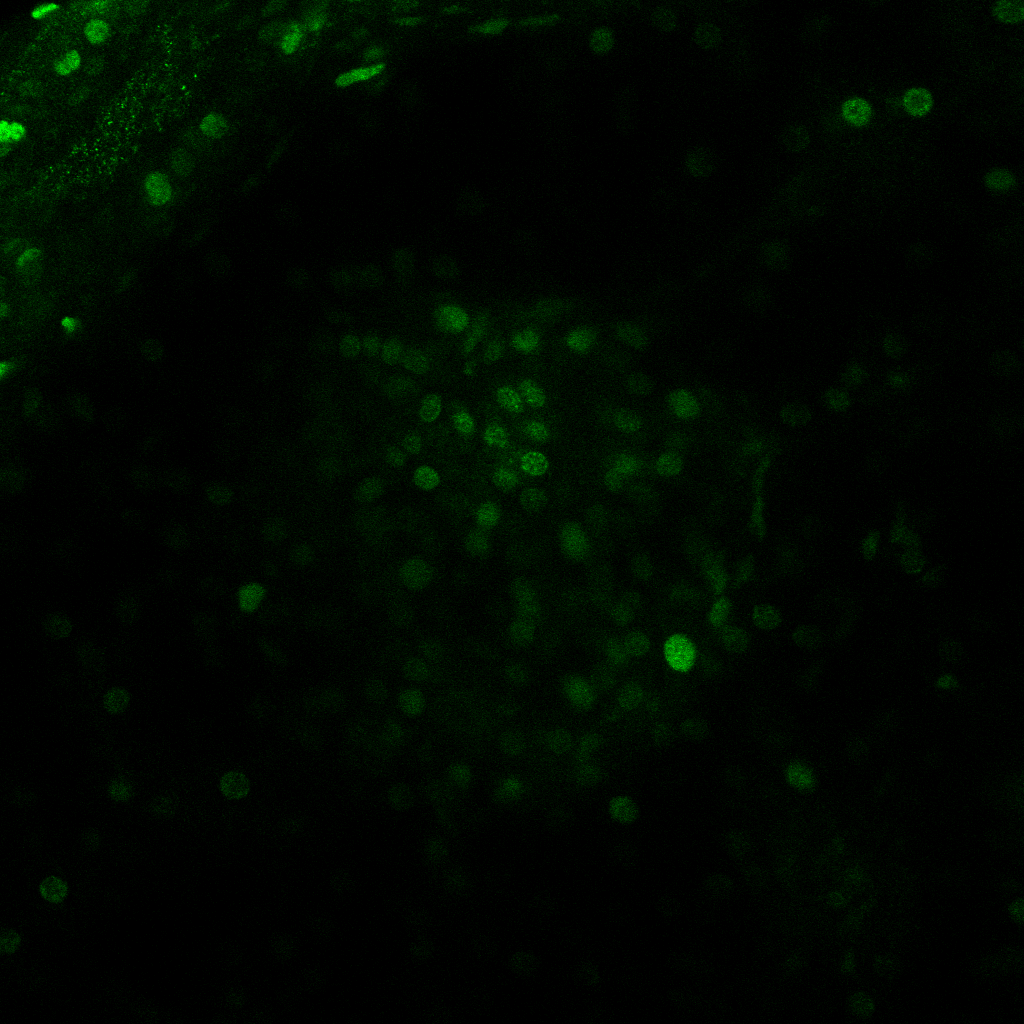

Supplement: Supplementary file 8 — Source data Fig. 1 [file 44318_2024_332_MOESM8_ESM.zip › Figure 1/1D/single plane/p35 25 dpf PCNA.tif]

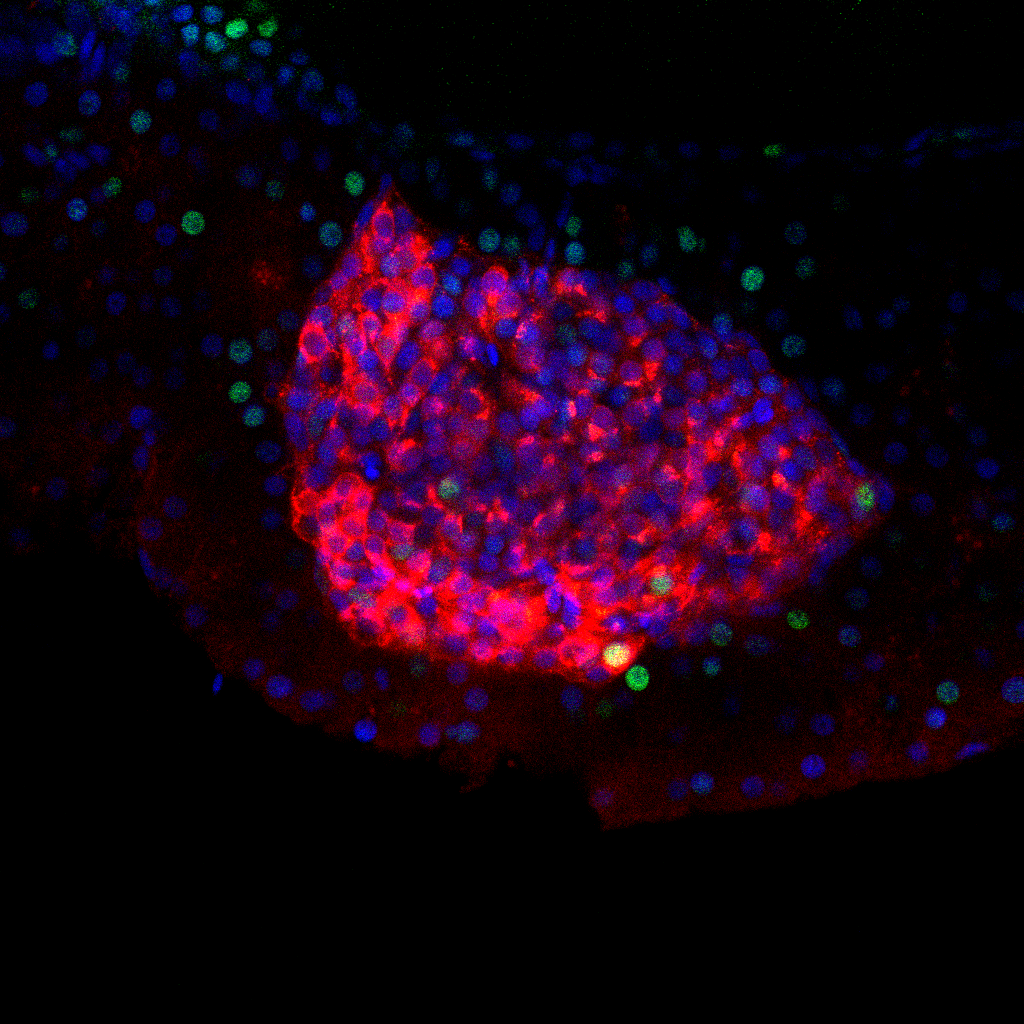

Supplement: Supplementary file 8 — Source data Fig. 1 [file 44318_2024_332_MOESM8_ESM.zip › Figure 1/1D/single plane/p35 30 dpf composite.tif]

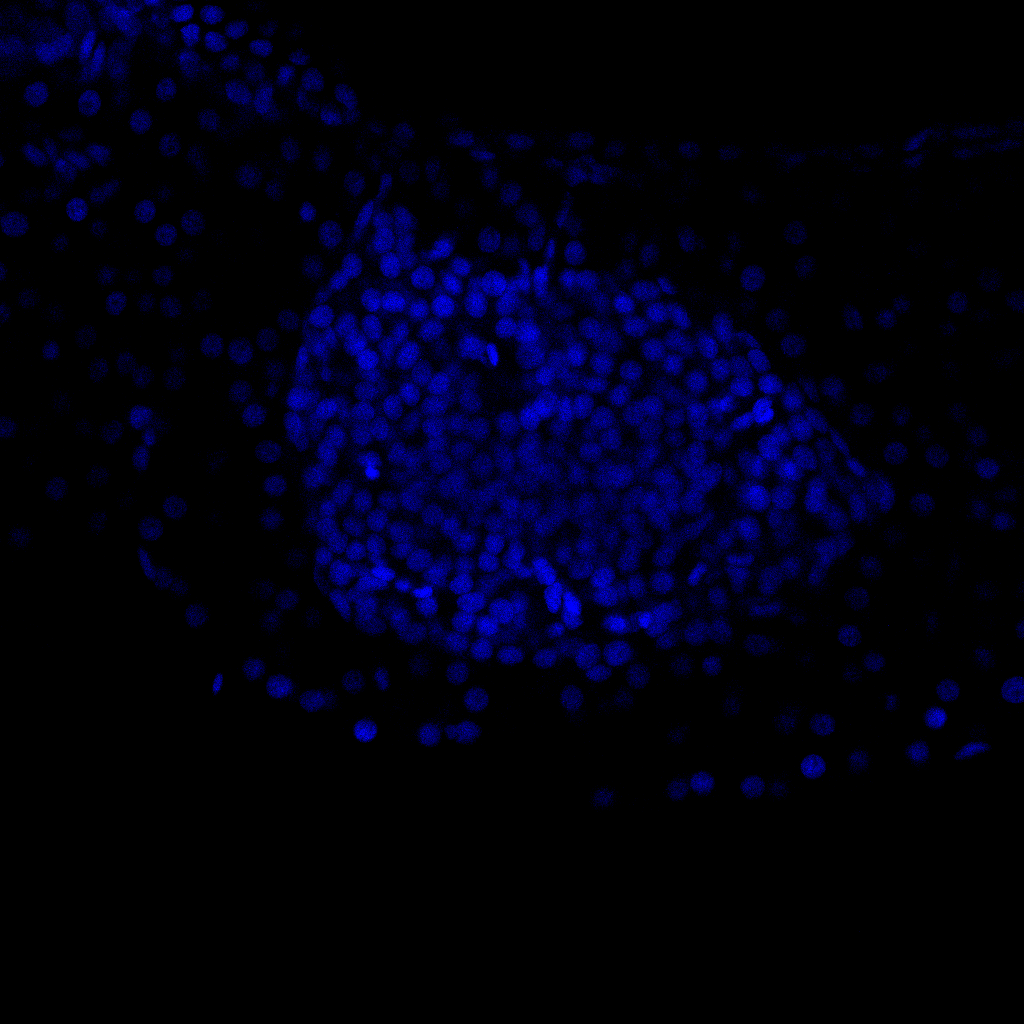

Supplement: Supplementary file 8 — Source data Fig. 1 [file 44318_2024_332_MOESM8_ESM.zip › Figure 1/1D/single plane/p35 30 dpf Hoechst.tif]

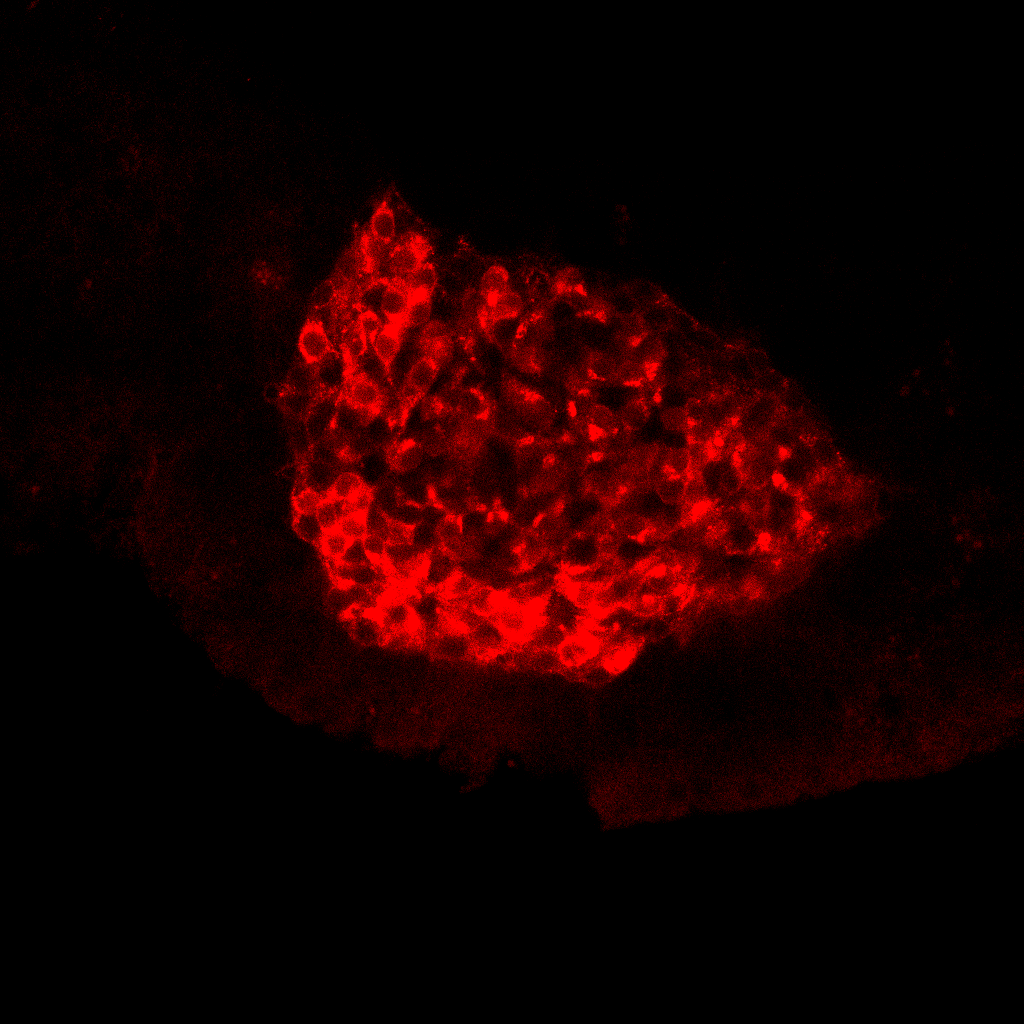

Supplement: Supplementary file 8 — Source data Fig. 1 [file 44318_2024_332_MOESM8_ESM.zip › Figure 1/1D/single plane/p35 30 dpf Ins.tif]

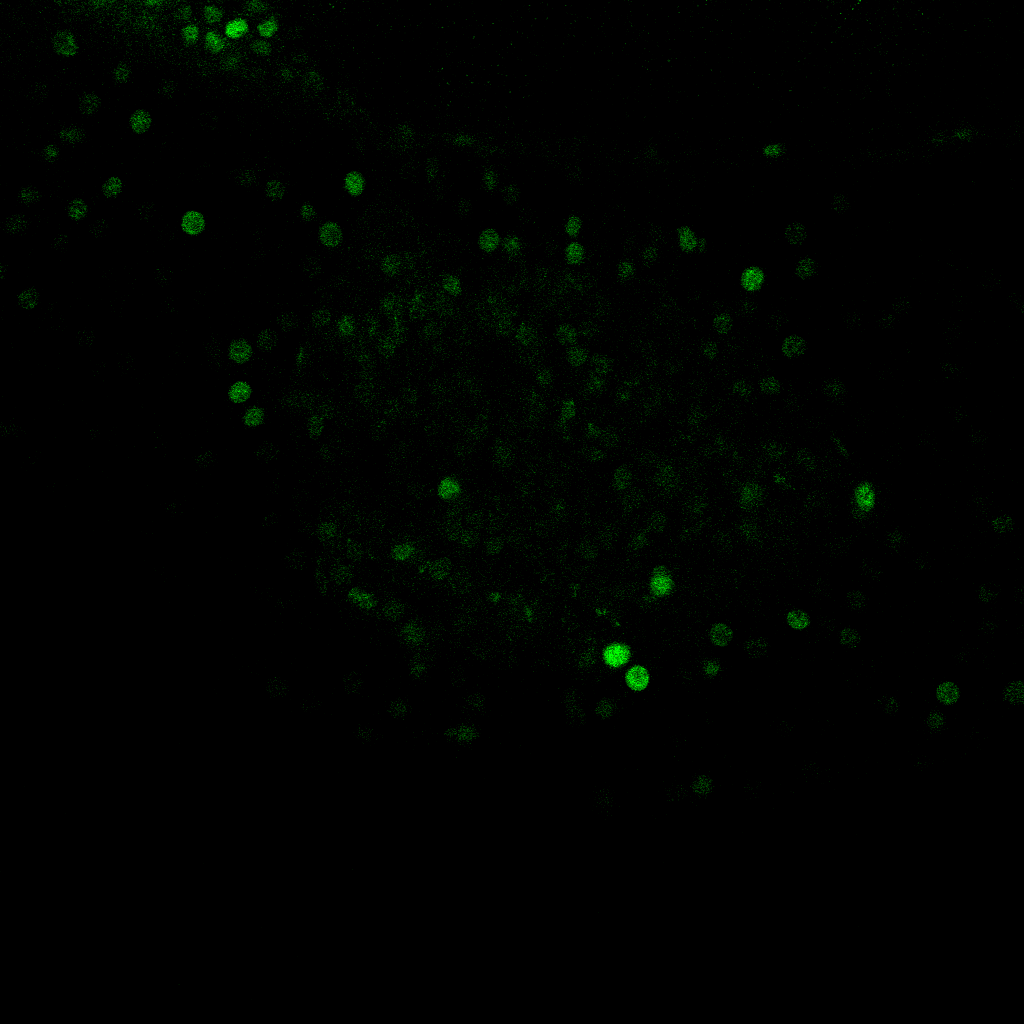

Supplement: Supplementary file 8 — Source data Fig. 1 [file 44318_2024_332_MOESM8_ESM.zip › Figure 1/1D/single plane/p35 30 dpf PCNA.tif]

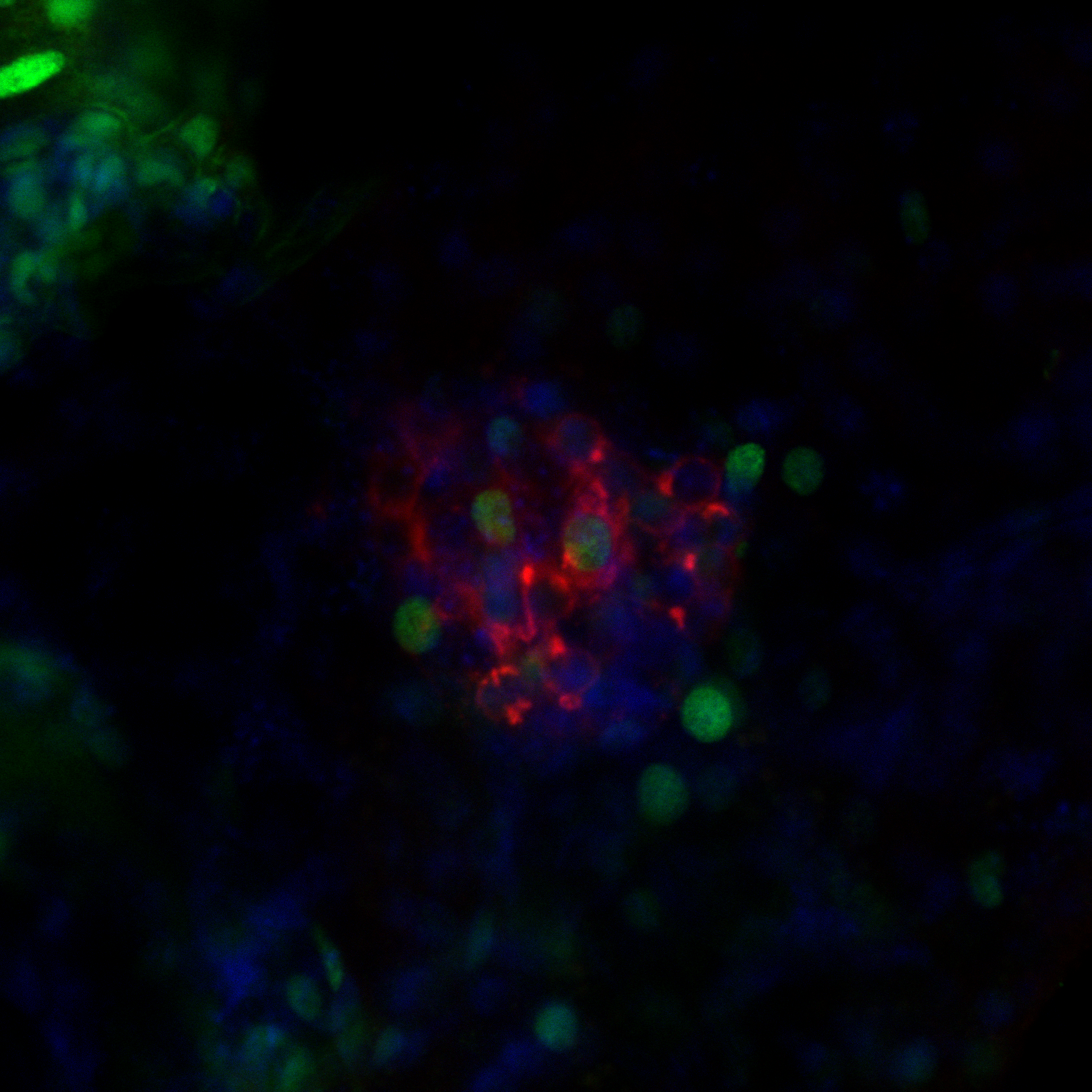

Supplement: Supplementary file 8 — Source data Fig. 1 [file 44318_2024_332_MOESM8_ESM.zip › Figure 1/1D/single plane/WT 15 dpf composite.tif]

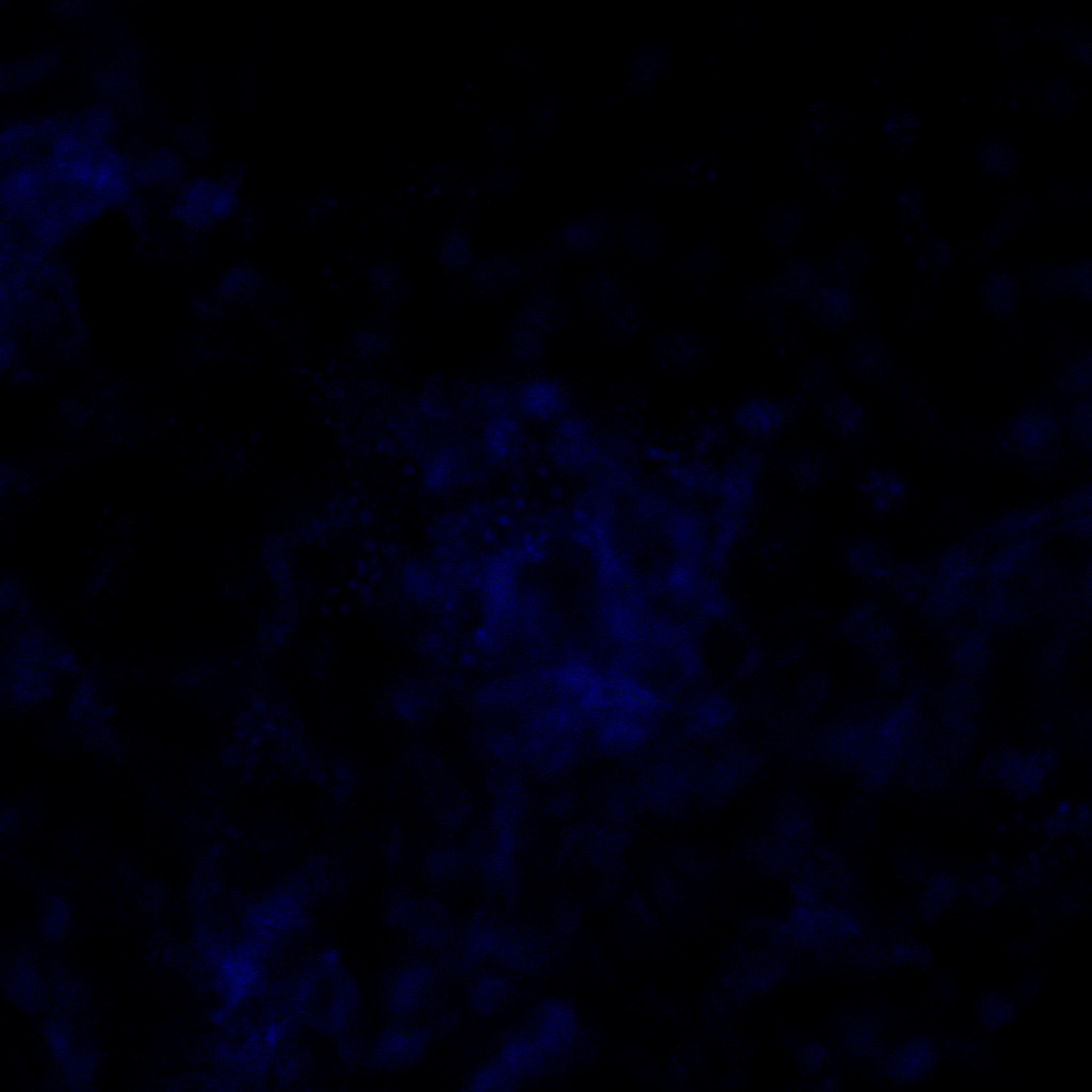

Supplement: Supplementary file 8 — Source data Fig. 1 [file 44318_2024_332_MOESM8_ESM.zip › Figure 1/1D/single plane/WT 15 dpf Hoechst.tif]

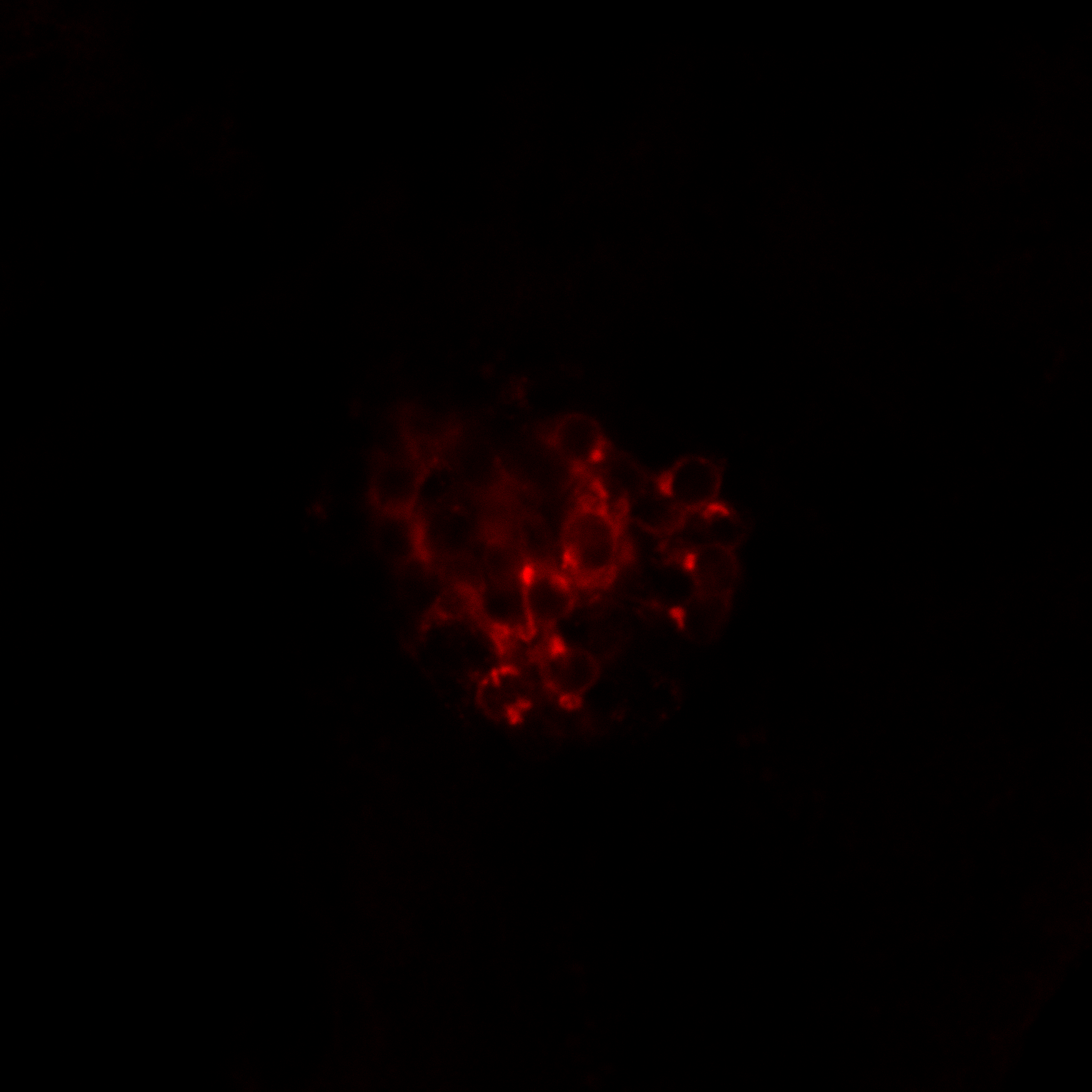

Supplement: Supplementary file 8 — Source data Fig. 1 [file 44318_2024_332_MOESM8_ESM.zip › Figure 1/1D/single plane/WT 15 dpf Ins.tif]

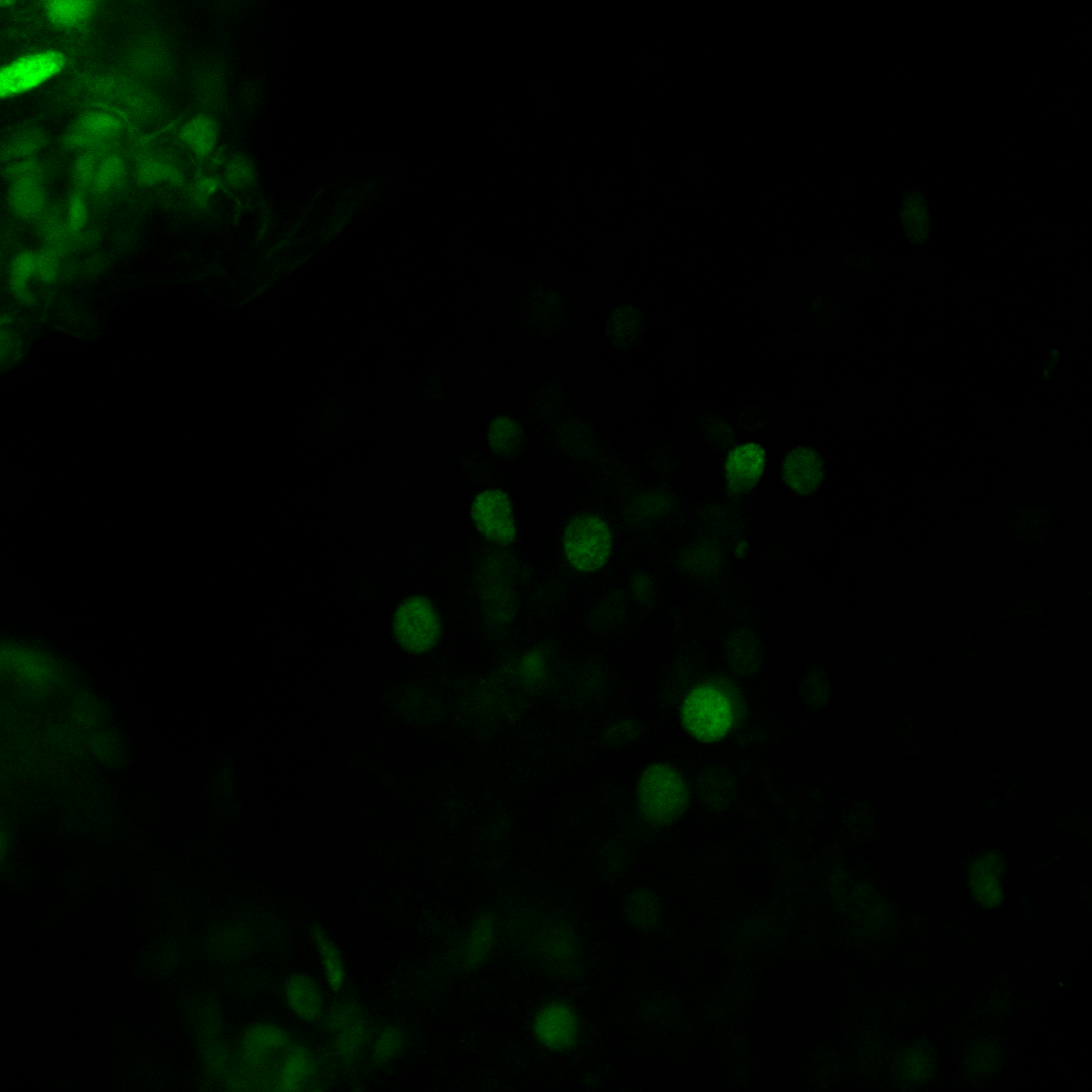

Supplement: Supplementary file 8 — Source data Fig. 1 [file 44318_2024_332_MOESM8_ESM.zip › Figure 1/1D/single plane/WT 15 dpf PCNA.tif]

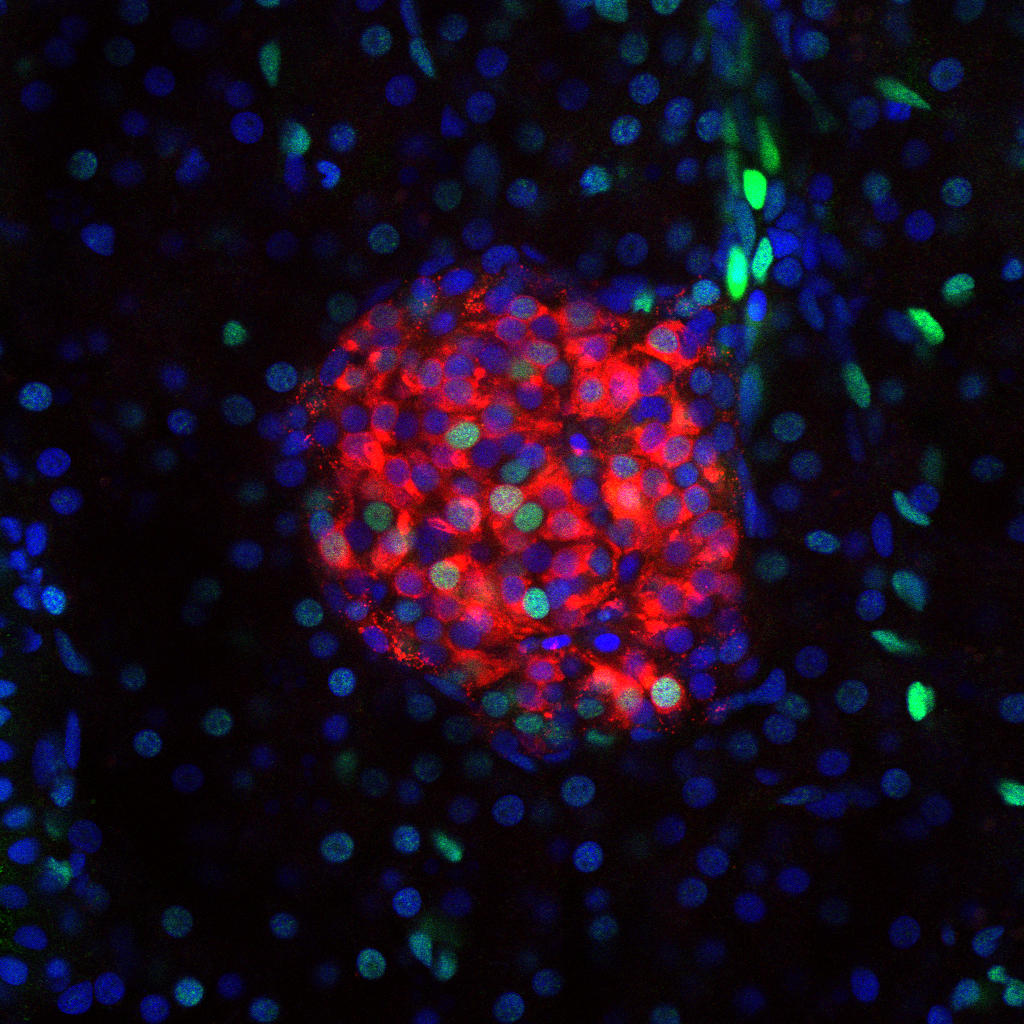

Supplement: Supplementary file 8 — Source data Fig. 1 [file 44318_2024_332_MOESM8_ESM.zip › Figure 1/1D/single plane/WT 25 dpf composite.tif]

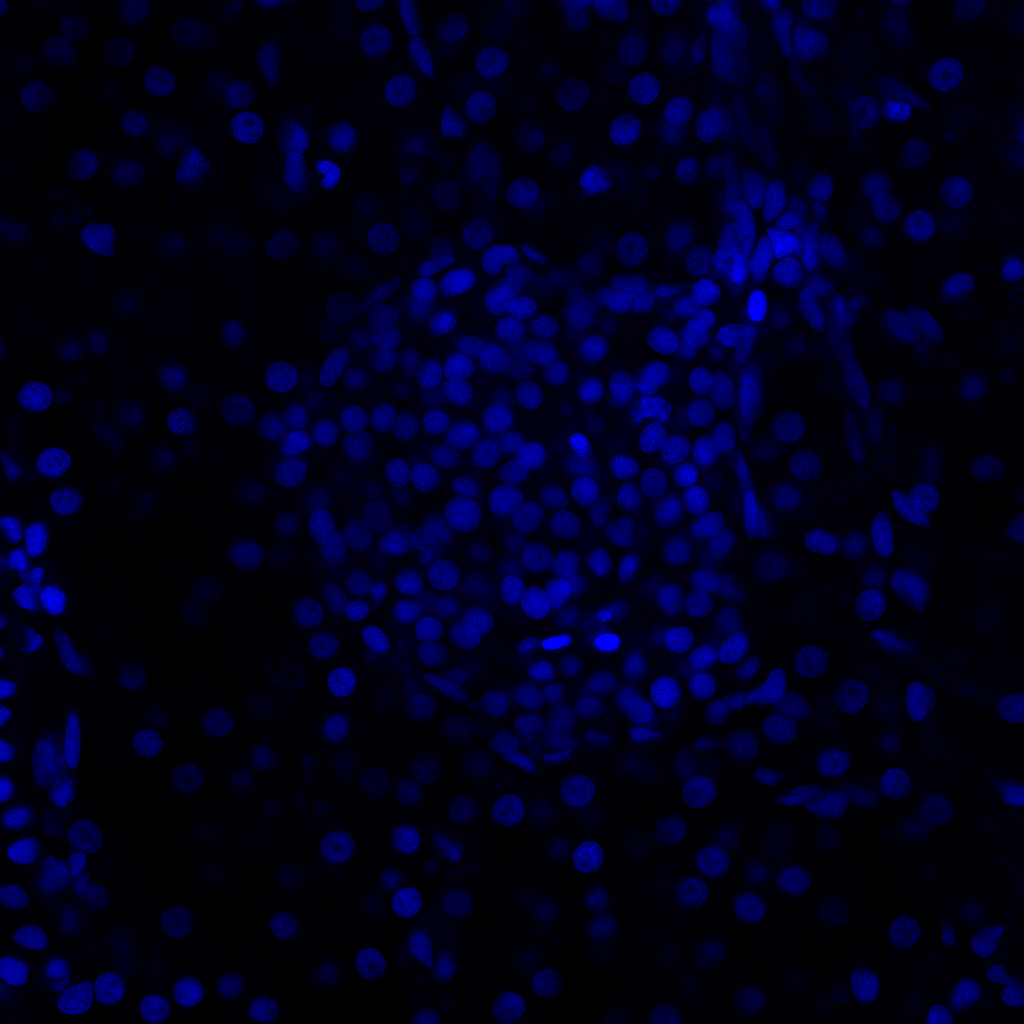

Supplement: Supplementary file 8 — Source data Fig. 1 [file 44318_2024_332_MOESM8_ESM.zip › Figure 1/1D/single plane/WT 25 dpf Hoechst.tif]

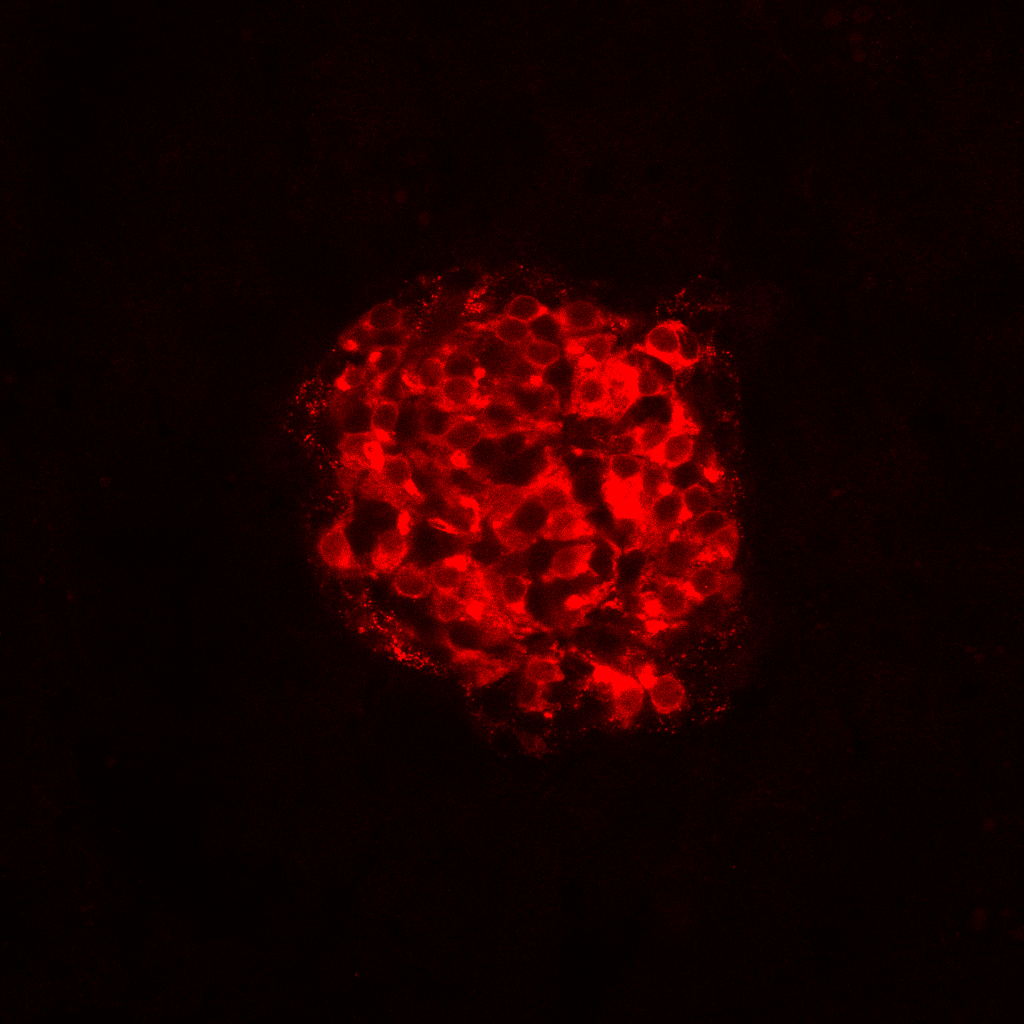

Supplement: Supplementary file 8 — Source data Fig. 1 [file 44318_2024_332_MOESM8_ESM.zip › Figure 1/1D/single plane/WT 25 dpf Ins.tif]

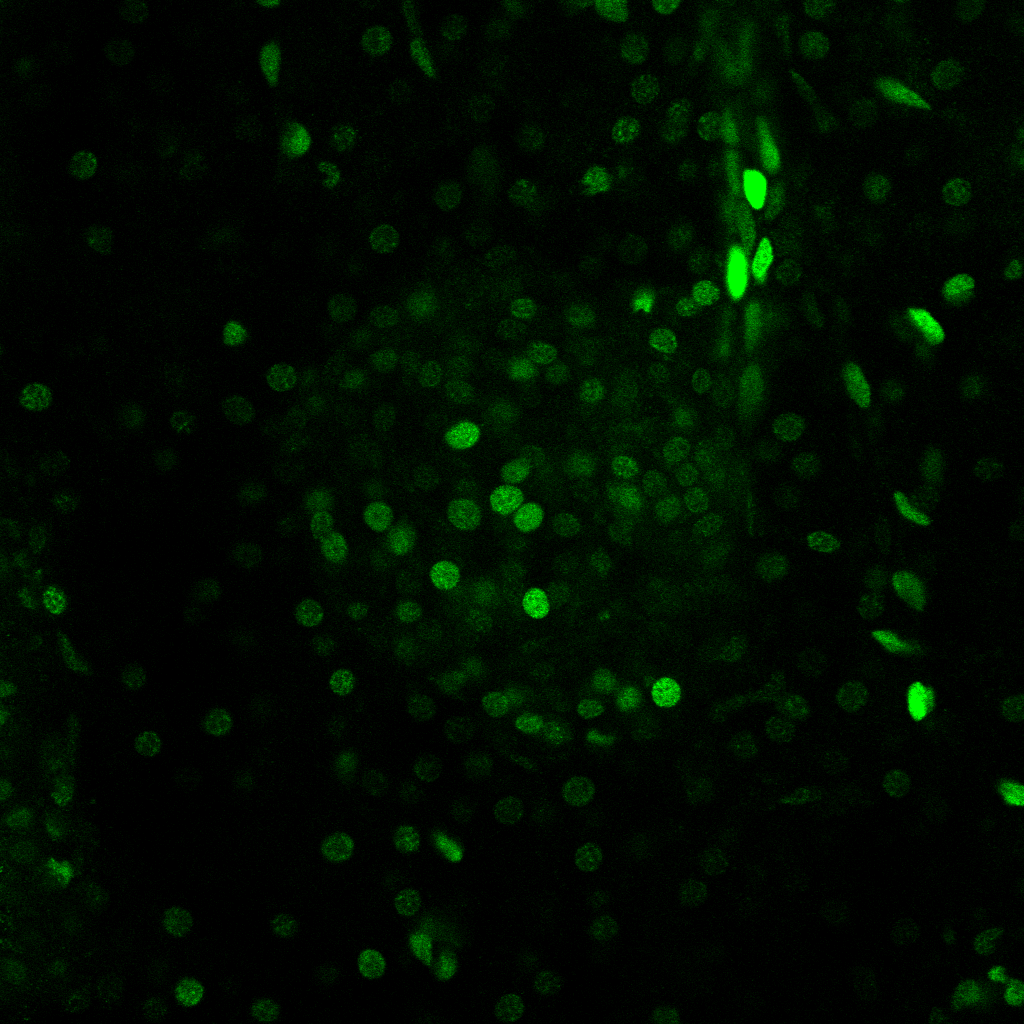

Supplement: Supplementary file 8 — Source data Fig. 1 [file 44318_2024_332_MOESM8_ESM.zip › Figure 1/1D/single plane/WT 25 dpf PCNA.tif]

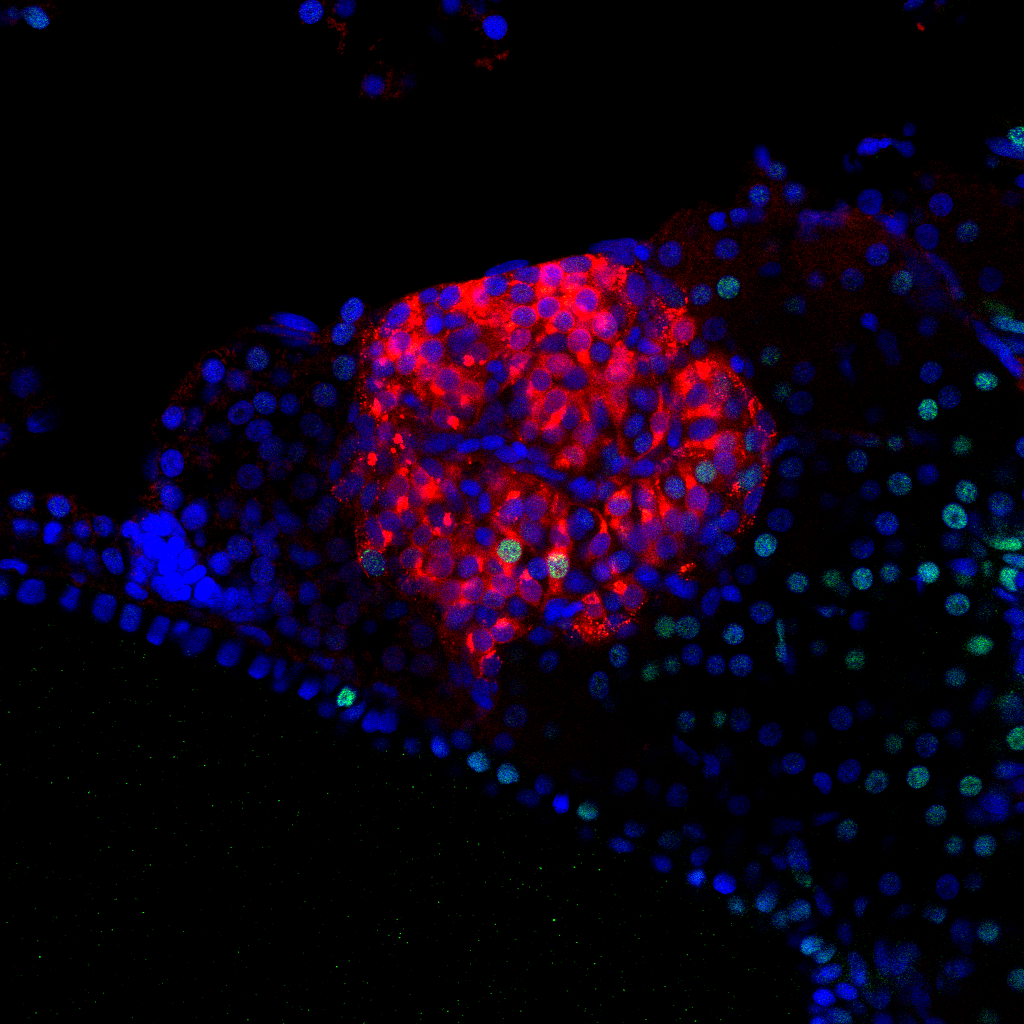

Supplement: Supplementary file 8 — Source data Fig. 1 [file 44318_2024_332_MOESM8_ESM.zip › Figure 1/1D/single plane/WT 30 dpf composite.tif]

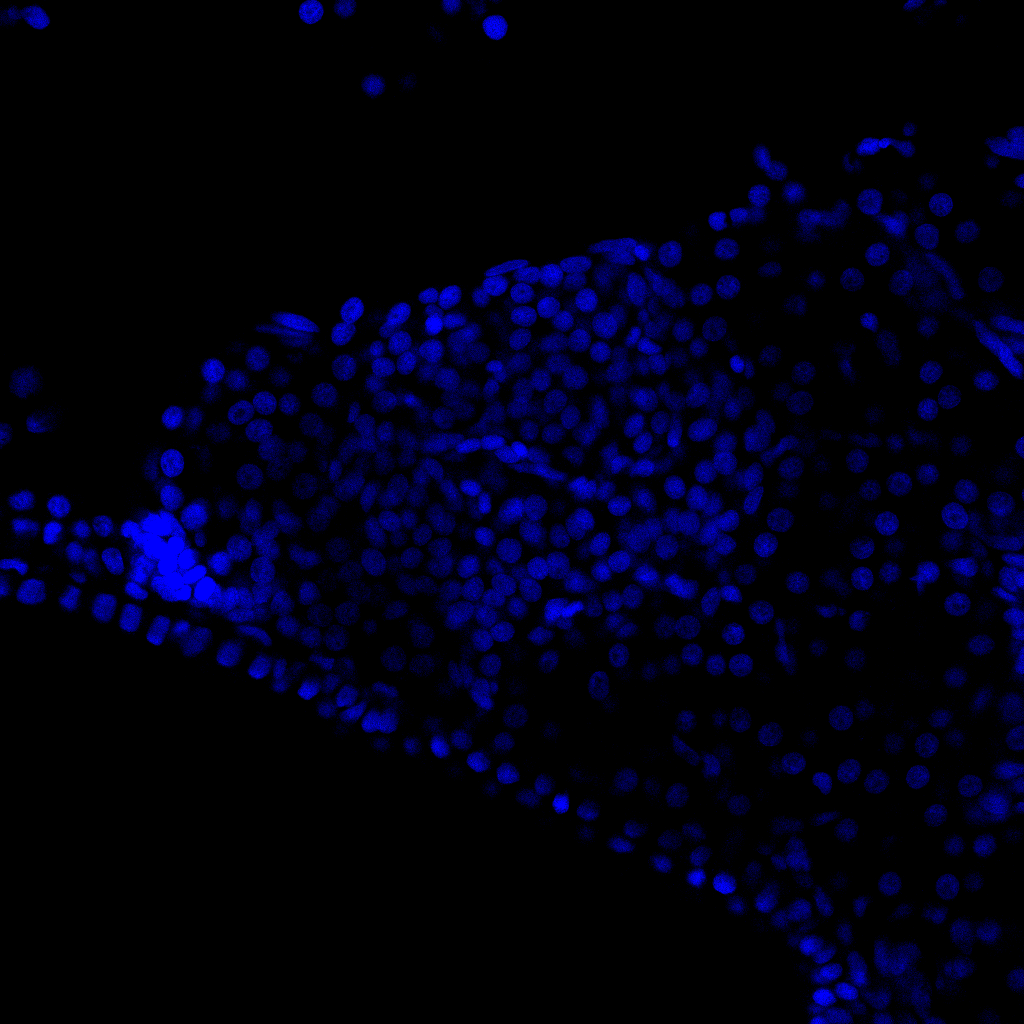

Supplement: Supplementary file 8 — Source data Fig. 1 [file 44318_2024_332_MOESM8_ESM.zip › Figure 1/1D/single plane/WT 30 dpf Hoechst.tif]

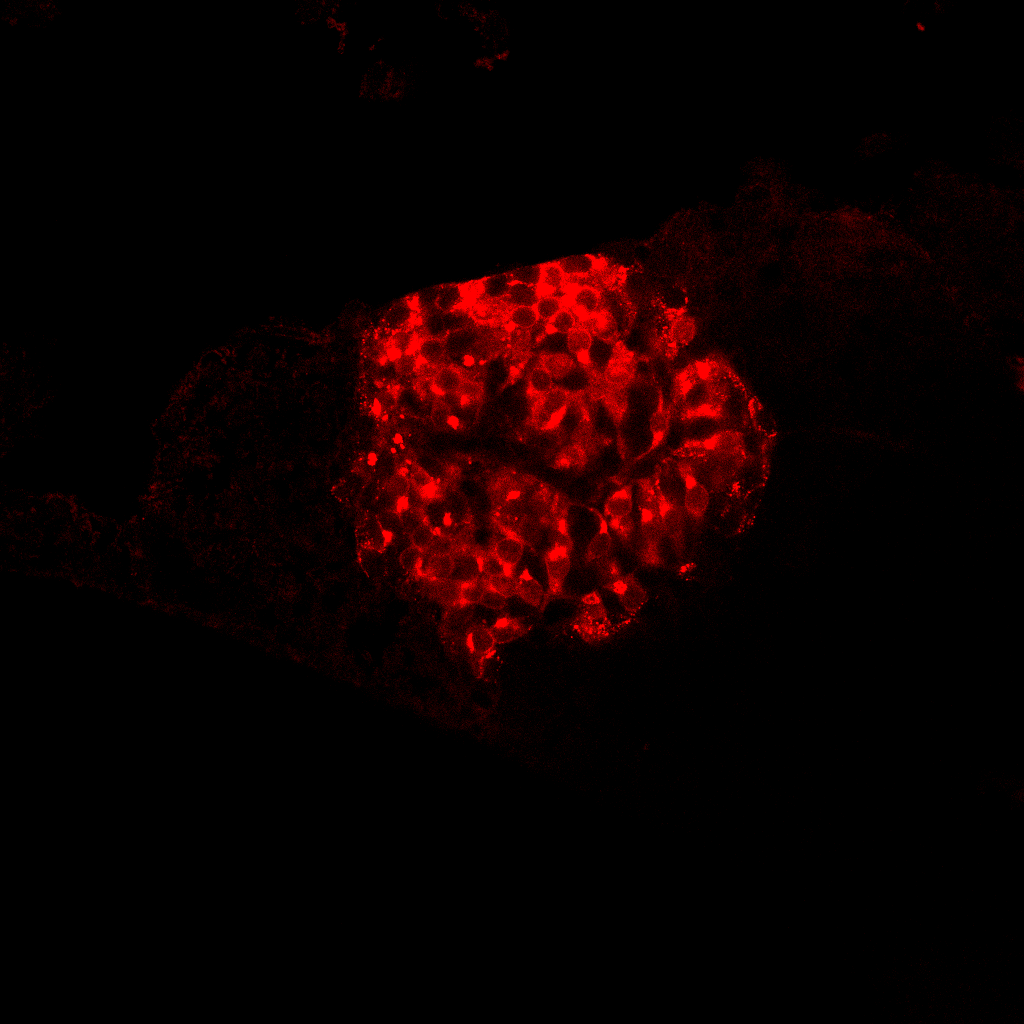

Supplement: Supplementary file 8 — Source data Fig. 1 [file 44318_2024_332_MOESM8_ESM.zip › Figure 1/1D/single plane/WT 30 dpf Ins.tif]

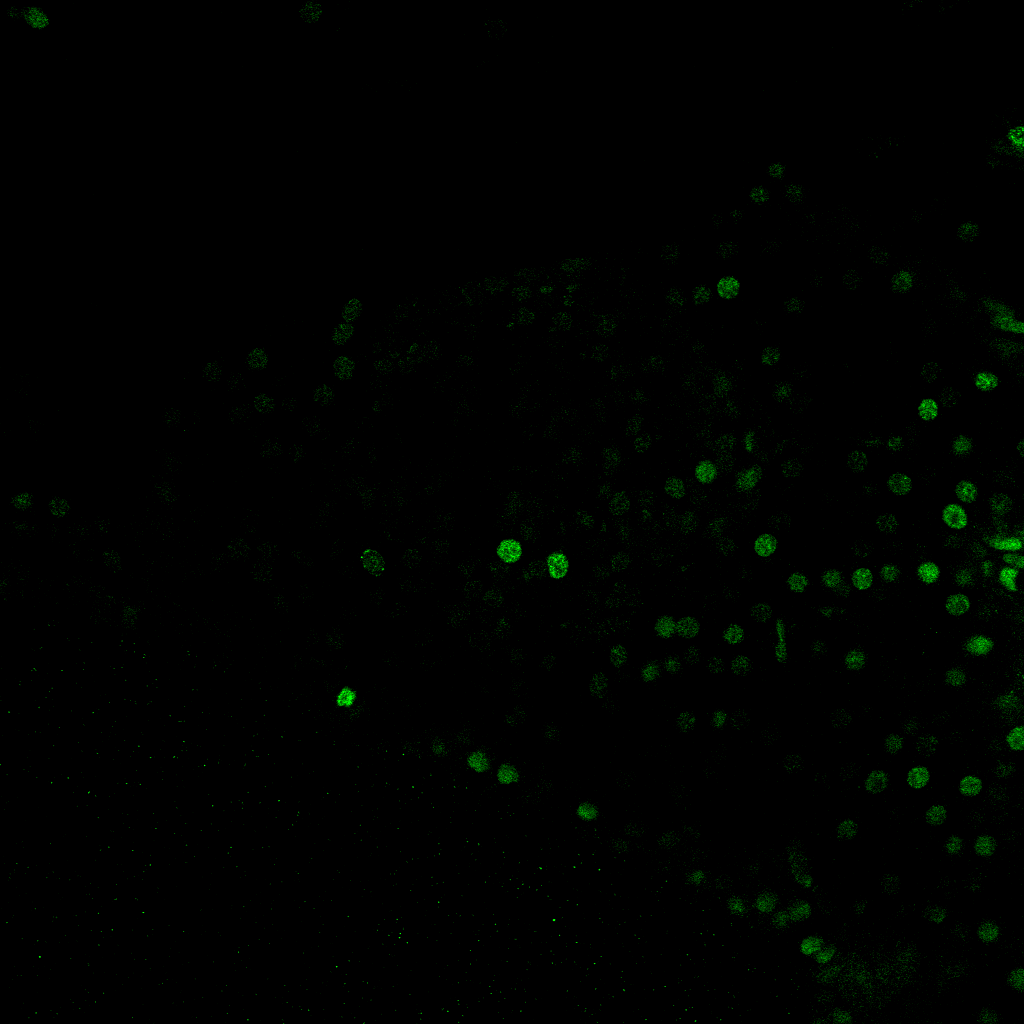

Supplement: Supplementary file 8 — Source data Fig. 1 [file 44318_2024_332_MOESM8_ESM.zip › Figure 1/1D/single plane/WT 30 dpf PCNA.tif]

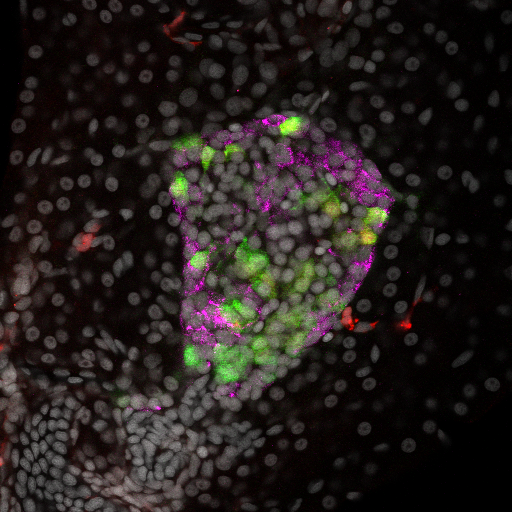

Supplement: Supplementary file 10 — Source data Fig. 3 [file 44318_2024_332_MOESM10_ESM.zip › Figure 3/A/21 dpf composite.tif]

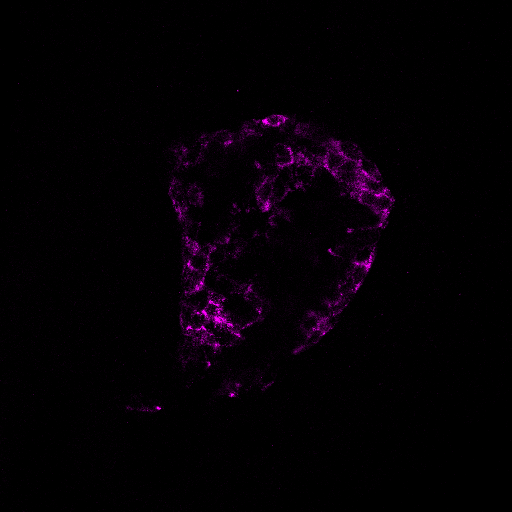

Supplement: Supplementary file 10 — Source data Fig. 3 [file 44318_2024_332_MOESM10_ESM.zip › Figure 3/A/21 dpf Glucagon.tif]

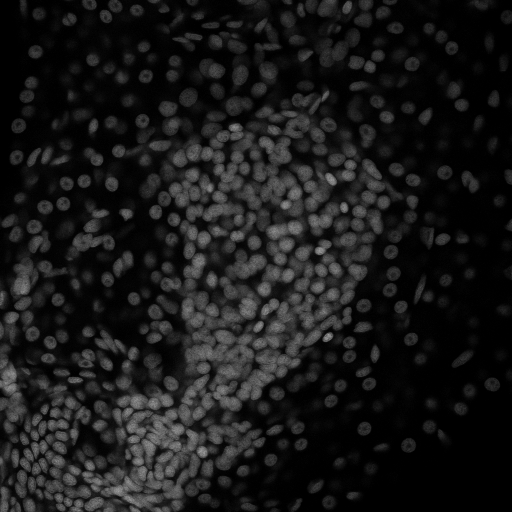

Supplement: Supplementary file 10 — Source data Fig. 3 [file 44318_2024_332_MOESM10_ESM.zip › Figure 3/A/21 dpf Hoechst.tif]

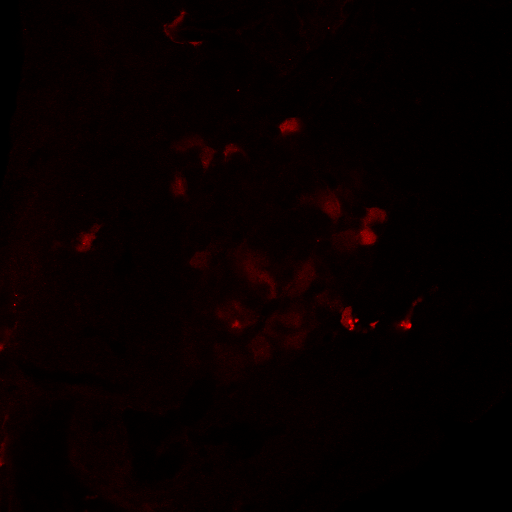

Supplement: Supplementary file 10 — Source data Fig. 3 [file 44318_2024_332_MOESM10_ESM.zip › Figure 3/A/21 dpf mCherry.tif]

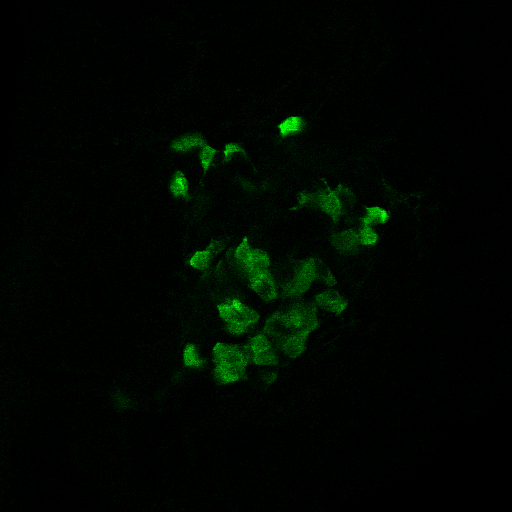

Supplement: Supplementary file 10 — Source data Fig. 3 [file 44318_2024_332_MOESM10_ESM.zip › Figure 3/A/21 dpf YFP.tif]

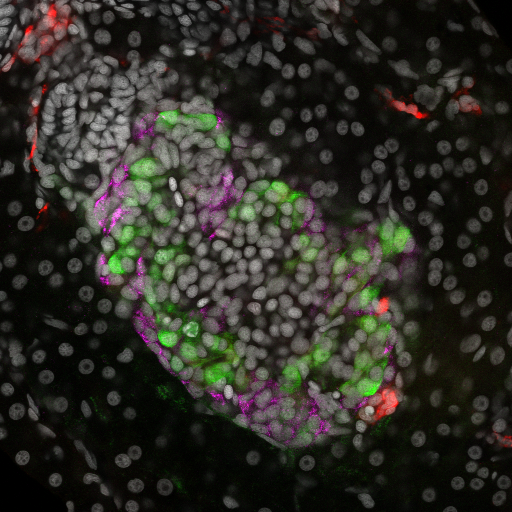

Supplement: Supplementary file 10 — Source data Fig. 3 [file 44318_2024_332_MOESM10_ESM.zip › Figure 3/A/33 dpf composite.tif]

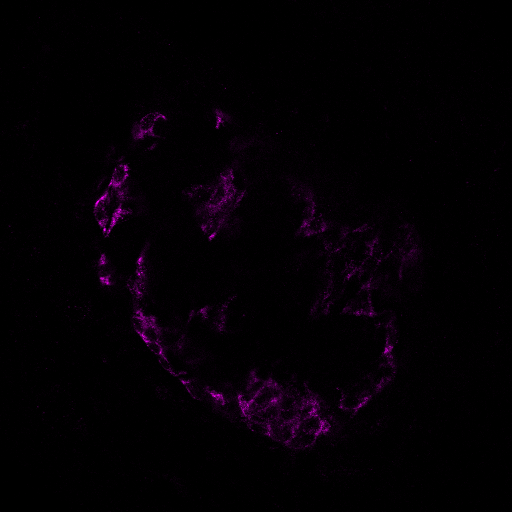

Supplement: Supplementary file 10 — Source data Fig. 3 [file 44318_2024_332_MOESM10_ESM.zip › Figure 3/A/33 dpf Glucagon.tif]

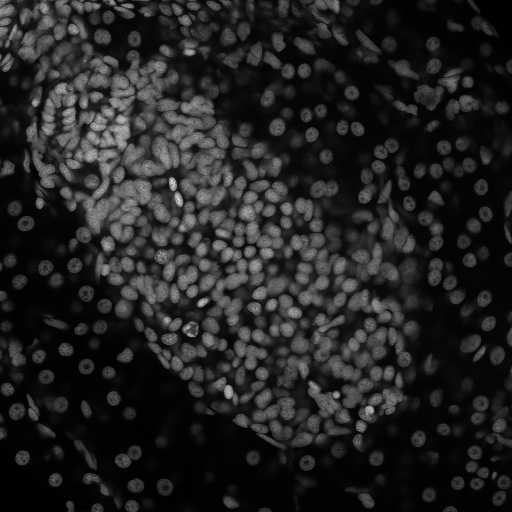

Supplement: Supplementary file 10 — Source data Fig. 3 [file 44318_2024_332_MOESM10_ESM.zip › Figure 3/A/33 dpf Hoechst.tif]

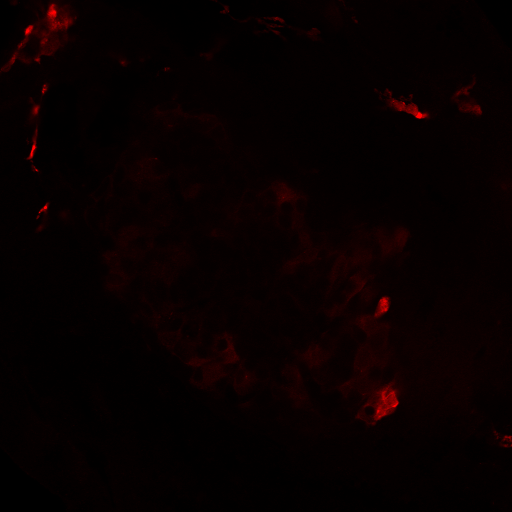

Supplement: Supplementary file 10 — Source data Fig. 3 [file 44318_2024_332_MOESM10_ESM.zip › Figure 3/A/33 dpf mCherry.tif]

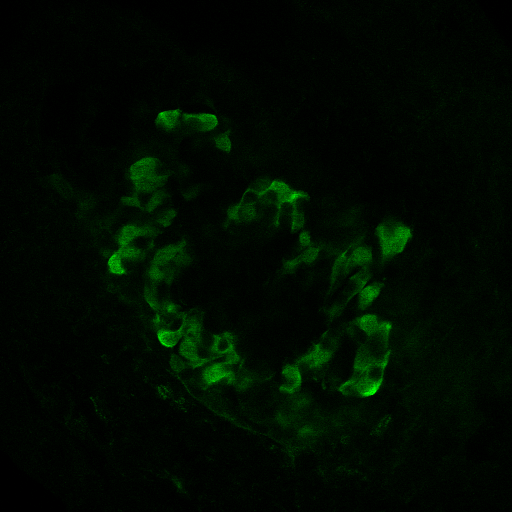

Supplement: Supplementary file 10 — Source data Fig. 3 [file 44318_2024_332_MOESM10_ESM.zip › Figure 3/A/33 dpf YFP.tif]

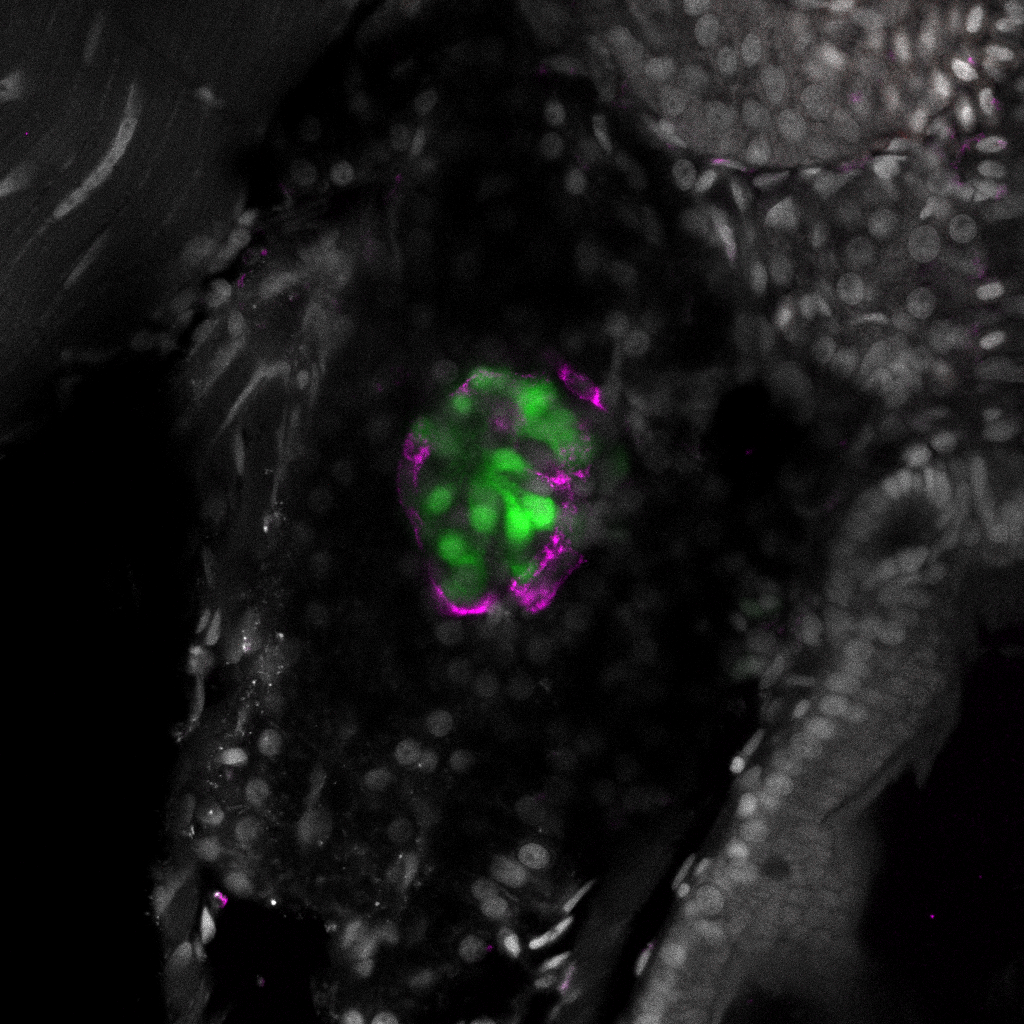

Supplement: Supplementary file 10 — Source data Fig. 3 [file 44318_2024_332_MOESM10_ESM.zip › Figure 3/A/5 dpf composite.tif]

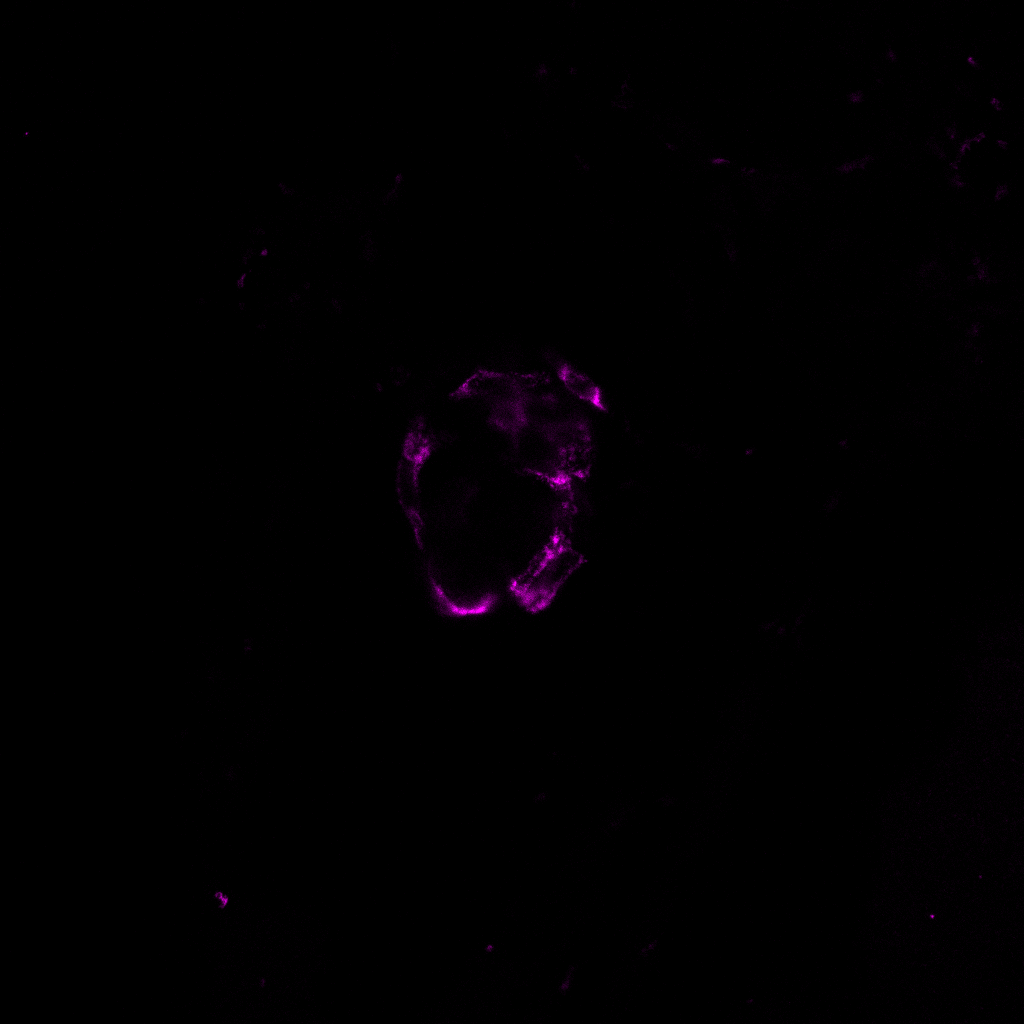

Supplement: Supplementary file 10 — Source data Fig. 3 [file 44318_2024_332_MOESM10_ESM.zip › Figure 3/A/5 dpf Glucagon.tif]

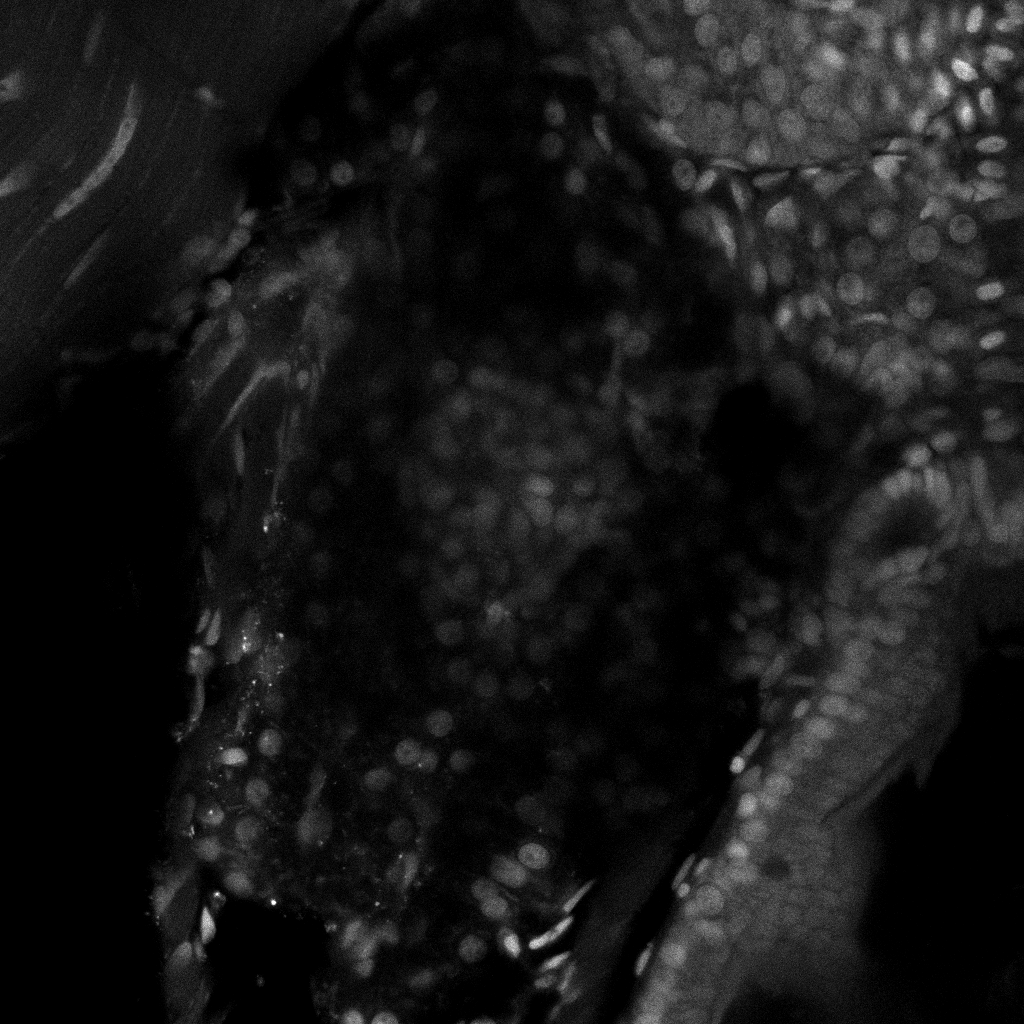

Supplement: Supplementary file 10 — Source data Fig. 3 [file 44318_2024_332_MOESM10_ESM.zip › Figure 3/A/5 dpf Hoechst.tif]

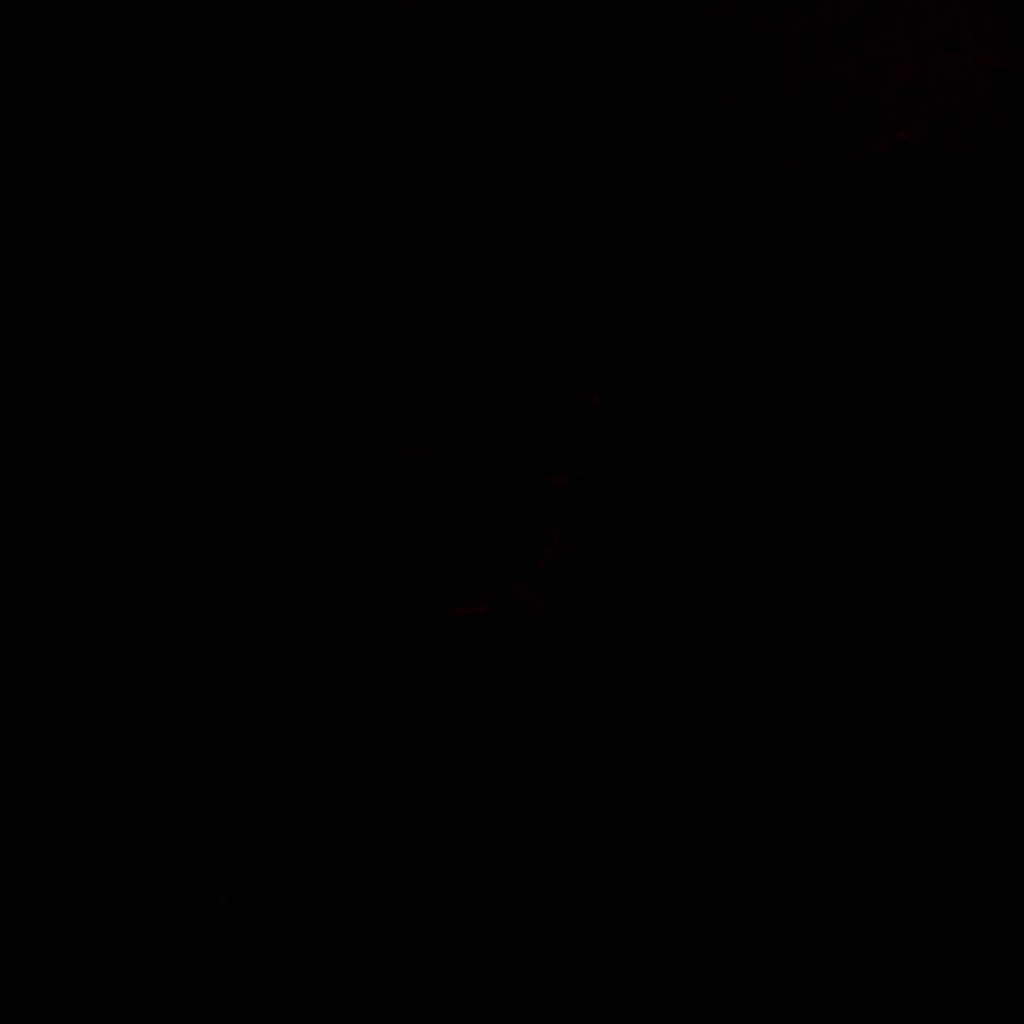

Supplement: Supplementary file 10 — Source data Fig. 3 [file 44318_2024_332_MOESM10_ESM.zip › Figure 3/A/5 dpf mCherry.tif]

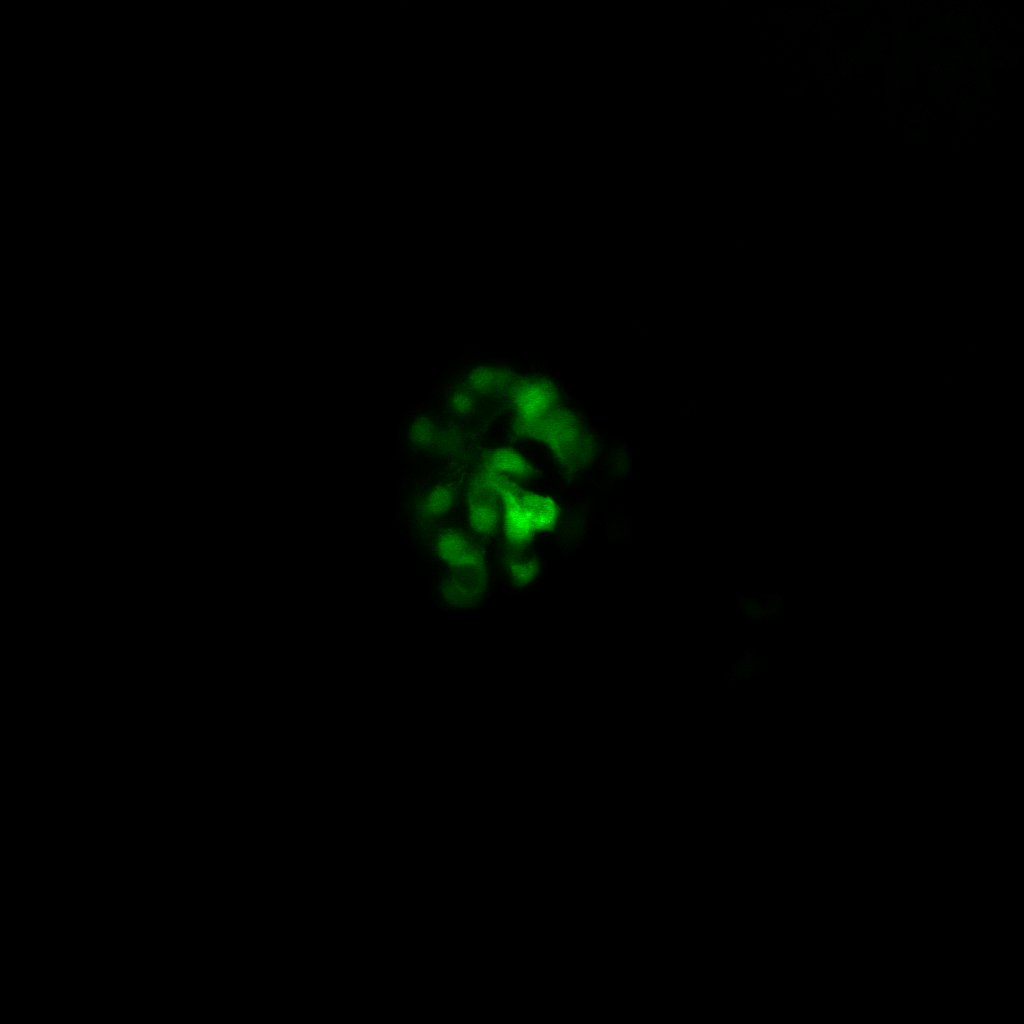

Supplement: Supplementary file 10 — Source data Fig. 3 [file 44318_2024_332_MOESM10_ESM.zip › Figure 3/A/5 dpf YFP.tif]

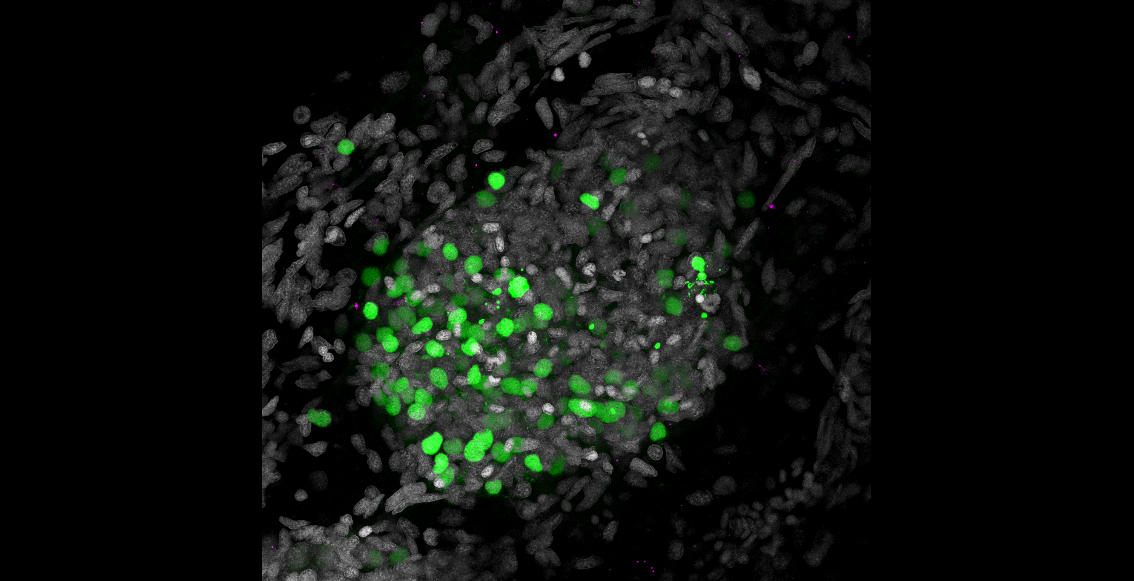

Supplement: Supplementary file 10 — Source data Fig. 3 [file 44318_2024_332_MOESM10_ESM.zip › Figure 3/C/Mutant composite.tif]

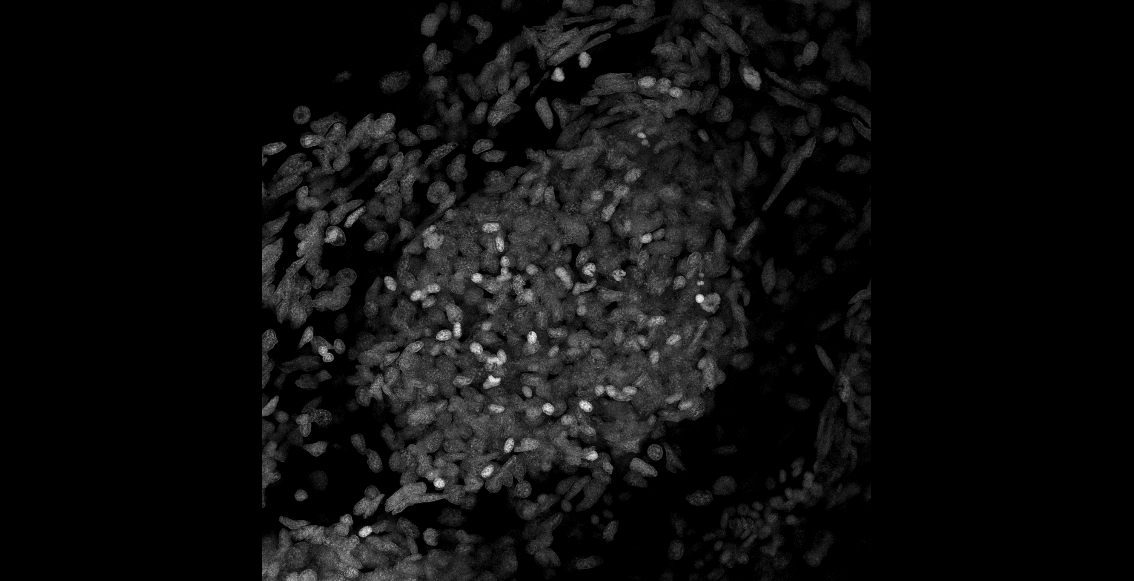

Supplement: Supplementary file 10 — Source data Fig. 3 [file 44318_2024_332_MOESM10_ESM.zip › Figure 3/C/Mutant Hoechst.tif]

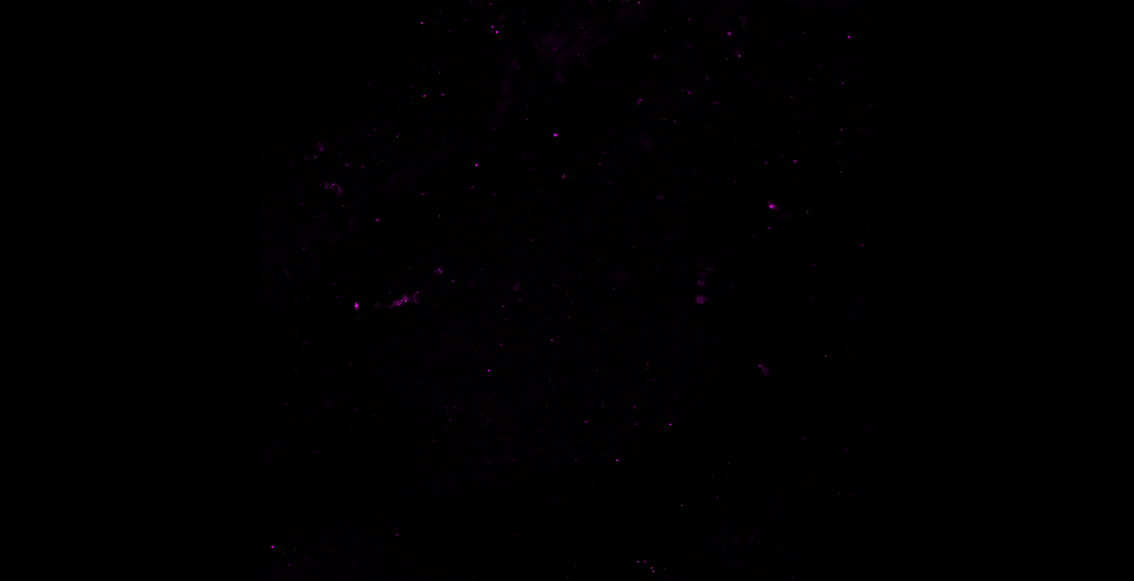

Supplement: Supplementary file 10 — Source data Fig. 3 [file 44318_2024_332_MOESM10_ESM.zip › Figure 3/C/Mutant TUNEL.tif]

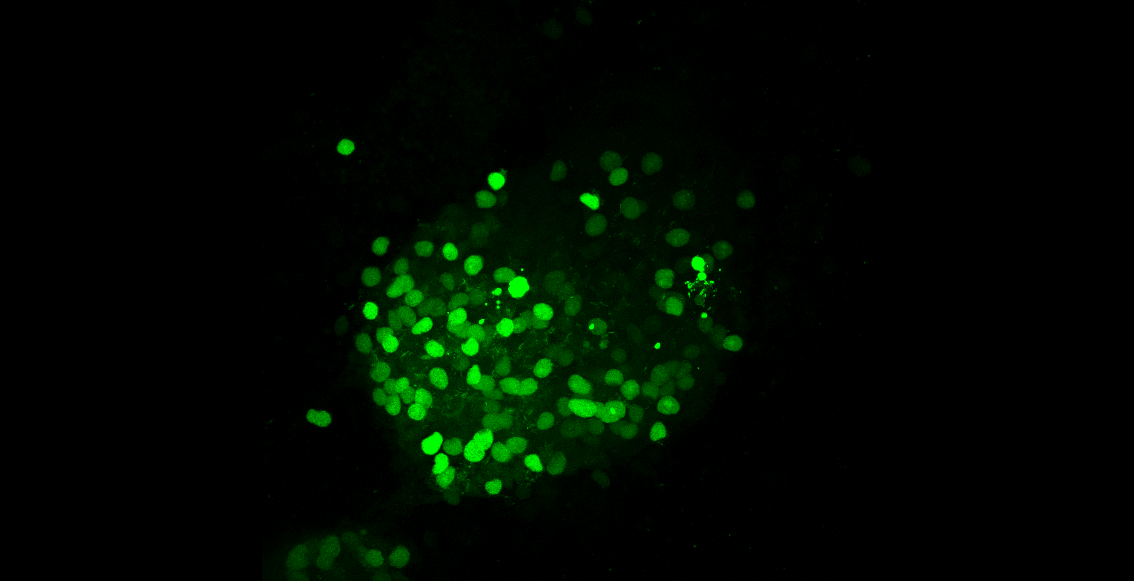

Supplement: Supplementary file 10 — Source data Fig. 3 [file 44318_2024_332_MOESM10_ESM.zip › Figure 3/C/Mutant YFP.tif]

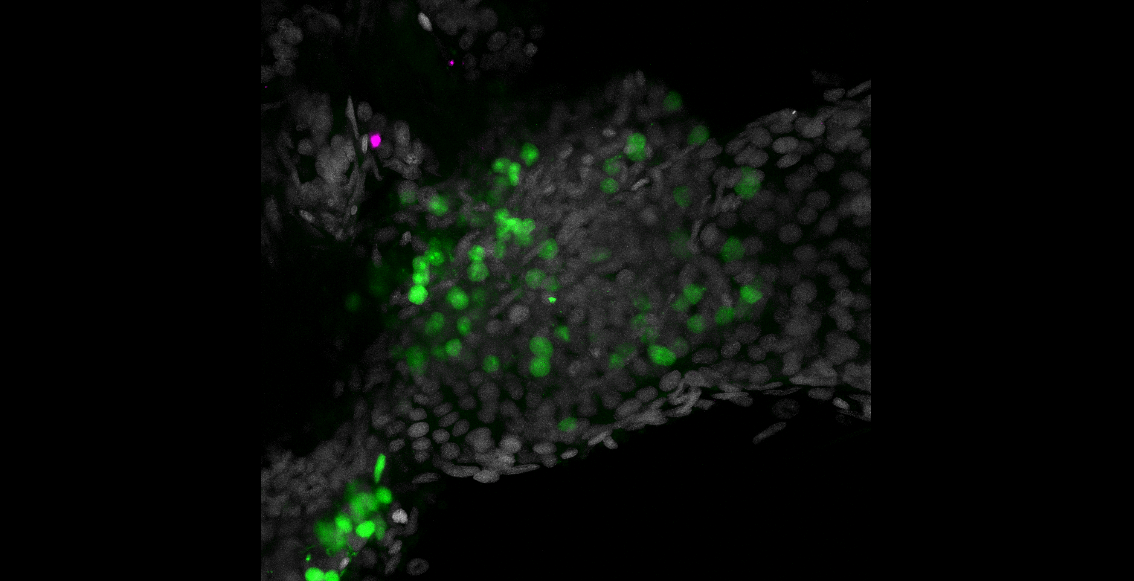

Supplement: Supplementary file 10 — Source data Fig. 3 [file 44318_2024_332_MOESM10_ESM.zip › Figure 3/C/WT composite.tif]

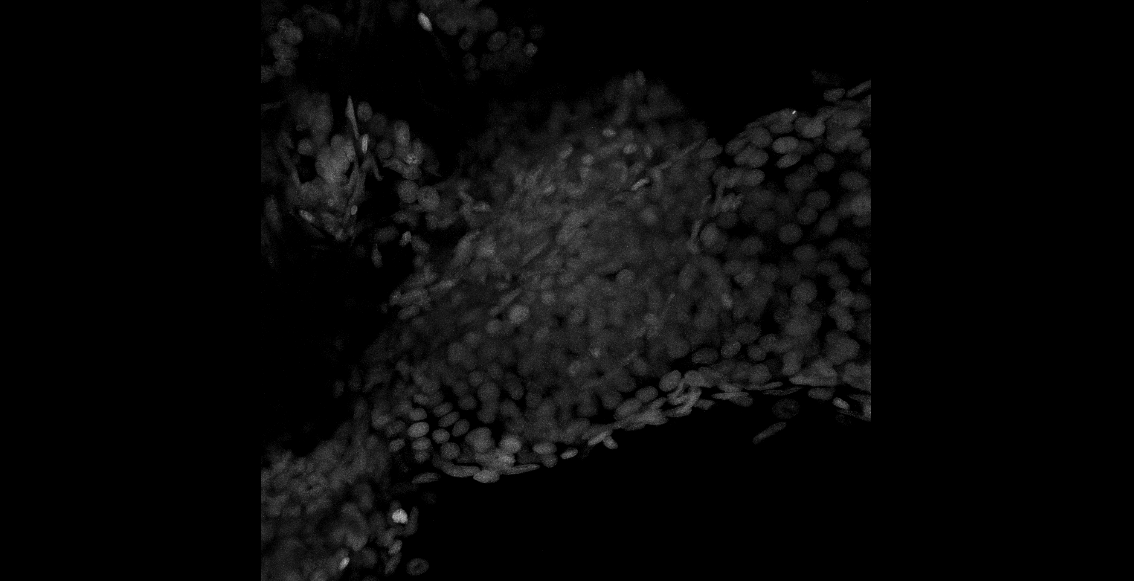

Supplement: Supplementary file 10 — Source data Fig. 3 [file 44318_2024_332_MOESM10_ESM.zip › Figure 3/C/WT Hoechst.tif]

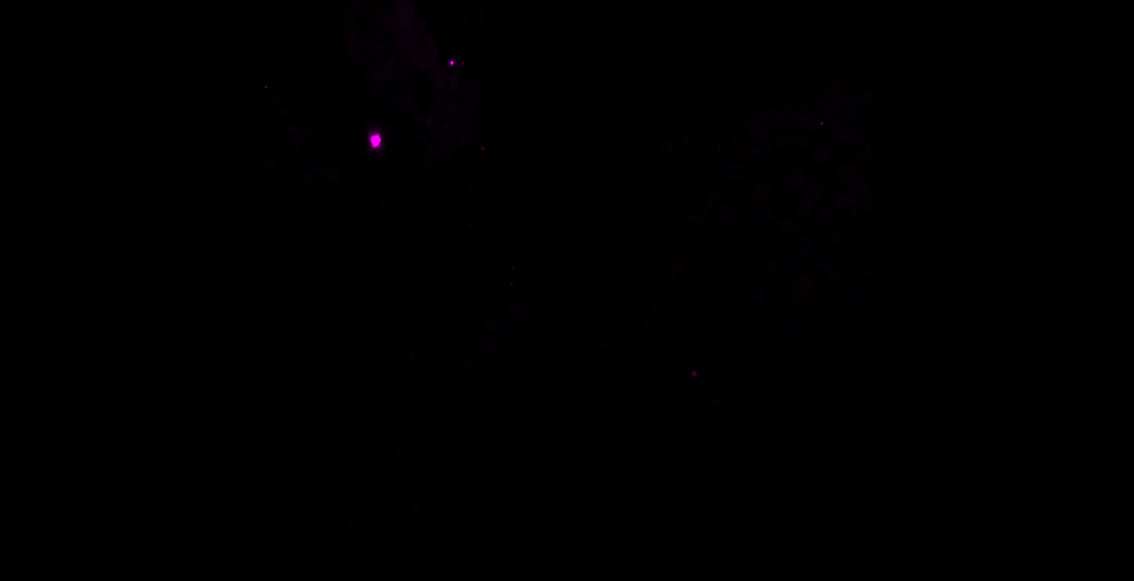

Supplement: Supplementary file 10 — Source data Fig. 3 [file 44318_2024_332_MOESM10_ESM.zip › Figure 3/C/WT TUNEL.tif]

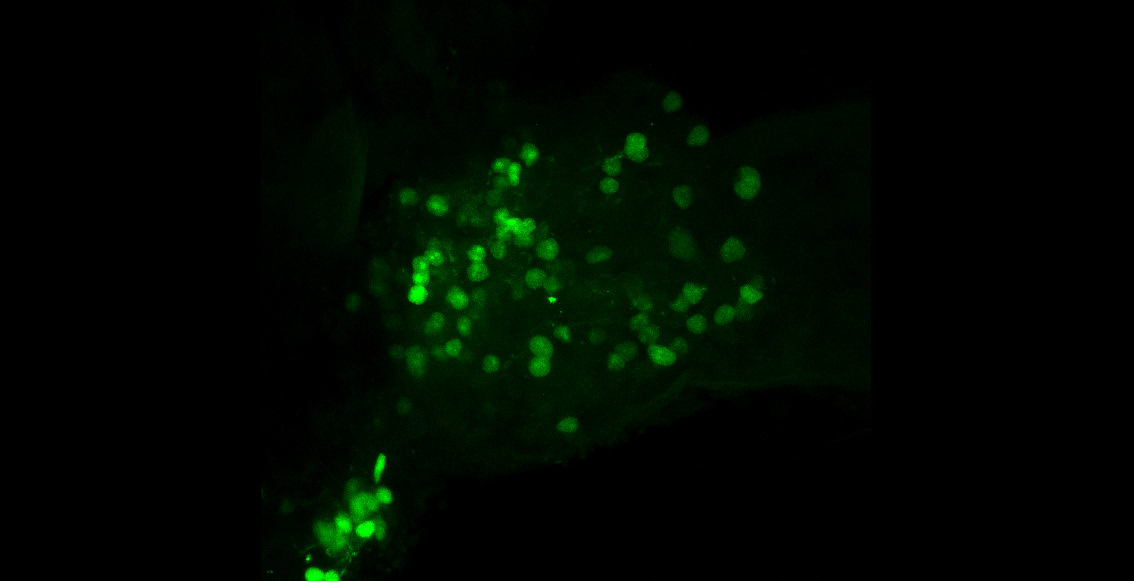

Supplement: Supplementary file 10 — Source data Fig. 3 [file 44318_2024_332_MOESM10_ESM.zip › Figure 3/C/WT YFP.tif]

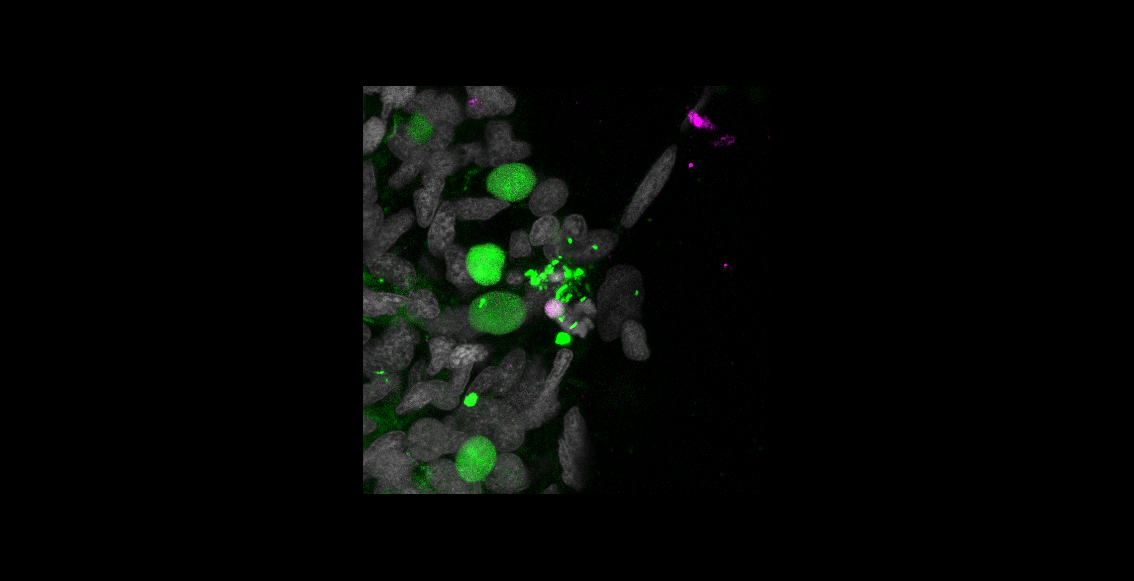

Supplement: Supplementary file 10 — Source data Fig. 3 [file 44318_2024_332_MOESM10_ESM.zip › Figure 3/C/zoom composite.tif]

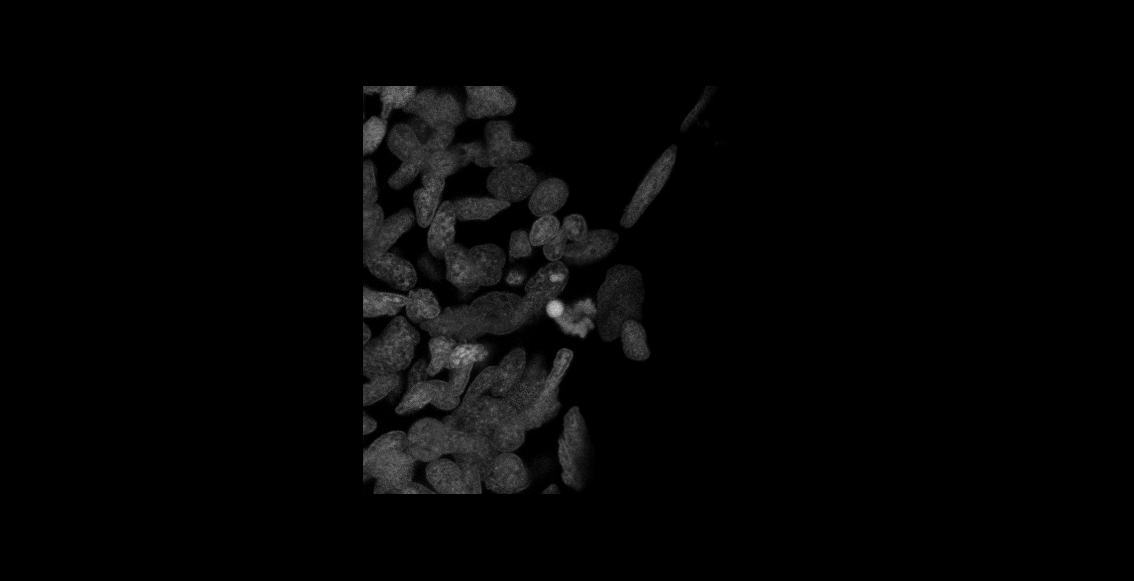

Supplement: Supplementary file 10 — Source data Fig. 3 [file 44318_2024_332_MOESM10_ESM.zip › Figure 3/C/zoom Hoechst.tif]

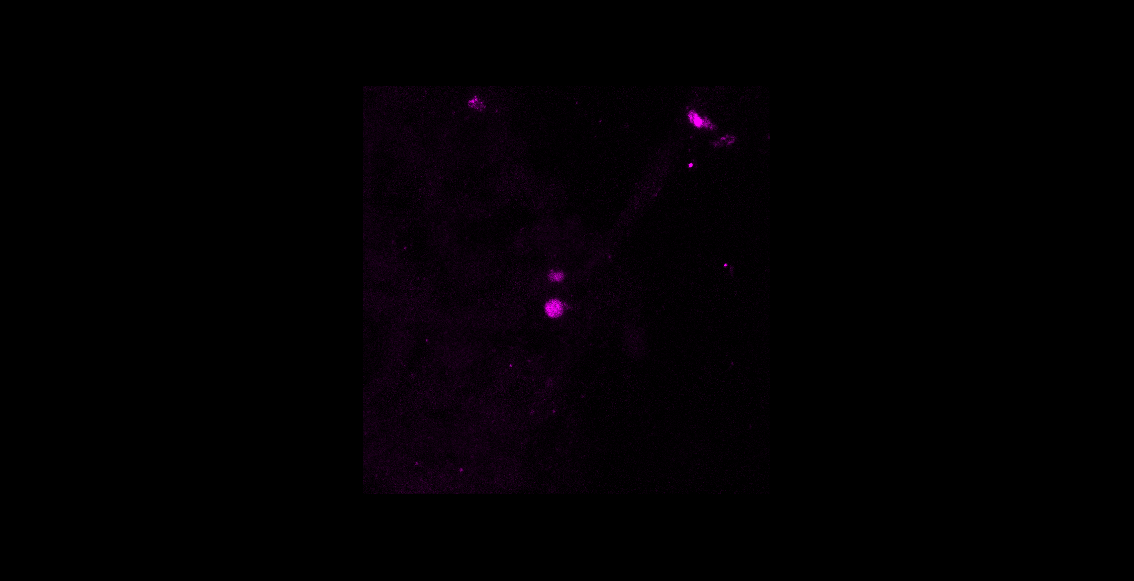

Supplement: Supplementary file 10 — Source data Fig. 3 [file 44318_2024_332_MOESM10_ESM.zip › Figure 3/C/zoom TUNEL.tif]

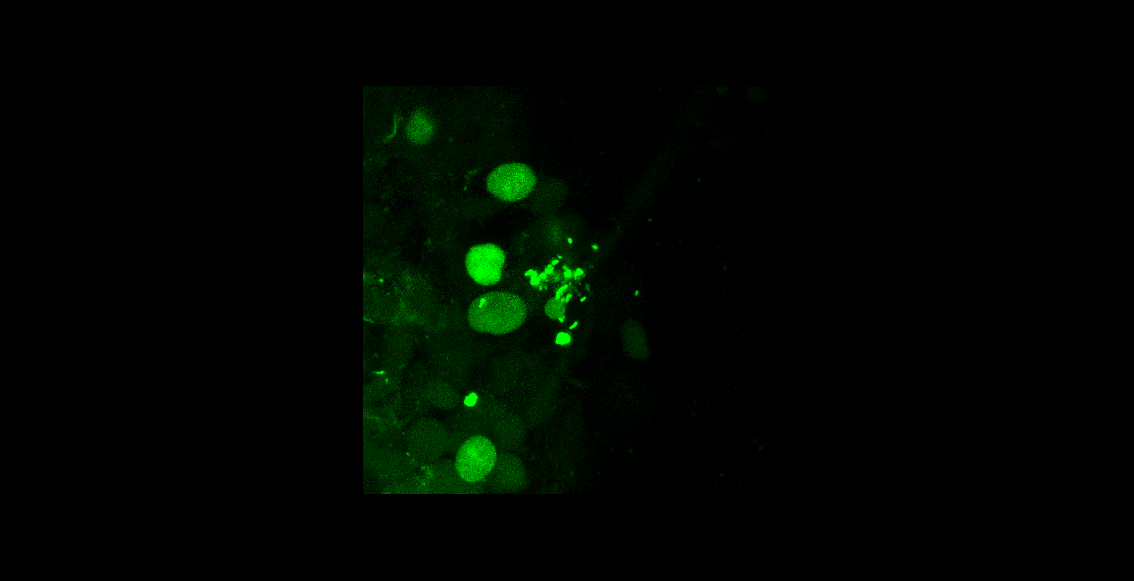

Supplement: Supplementary file 10 — Source data Fig. 3 [file 44318_2024_332_MOESM10_ESM.zip › Figure 3/C/zoom YFP.tif]

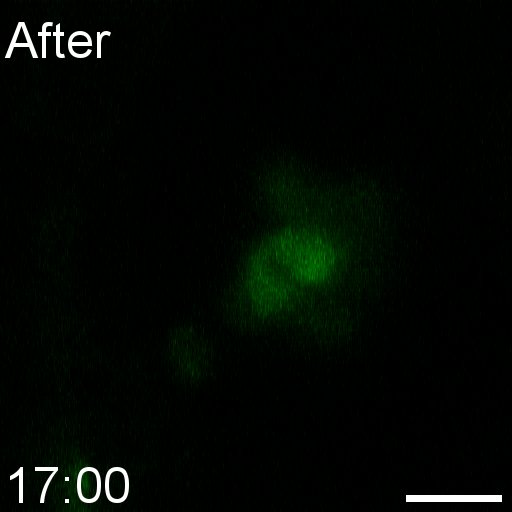

Supplement: Supplementary file 11 — Source data Fig. 4 [file 44318_2024_332_MOESM11_ESM.zip › Figure 4/4C/control after.jpg]

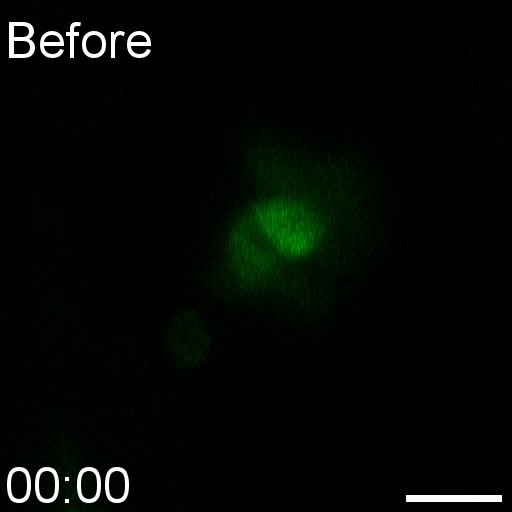

Supplement: Supplementary file 11 — Source data Fig. 4 [file 44318_2024_332_MOESM11_ESM.zip › Figure 4/4C/control before.jpg]

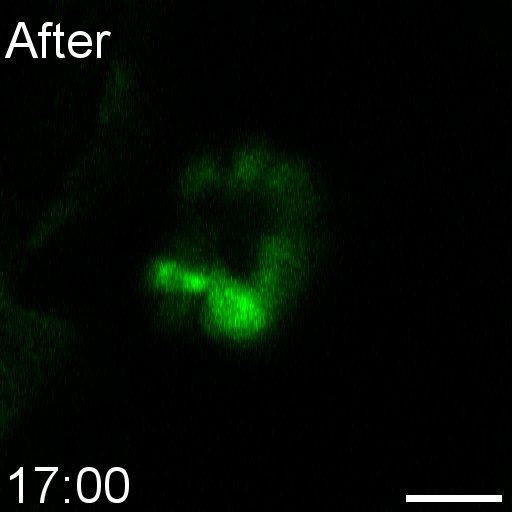

Supplement: Supplementary file 11 — Source data Fig. 4 [file 44318_2024_332_MOESM11_ESM.zip › Figure 4/4C/TRPV after.jpg]

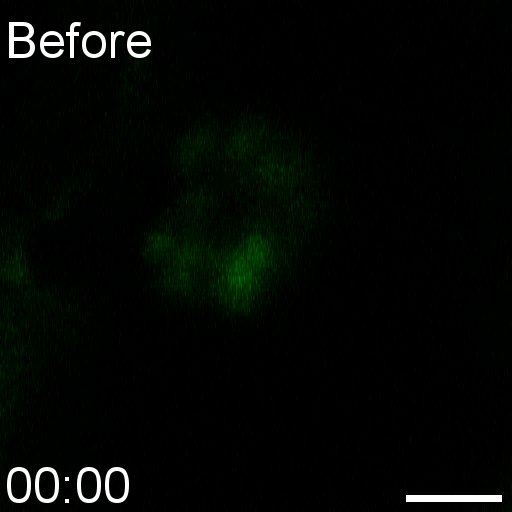

Supplement: Supplementary file 11 — Source data Fig. 4 [file 44318_2024_332_MOESM11_ESM.zip › Figure 4/4C/TRPV before.jpg]

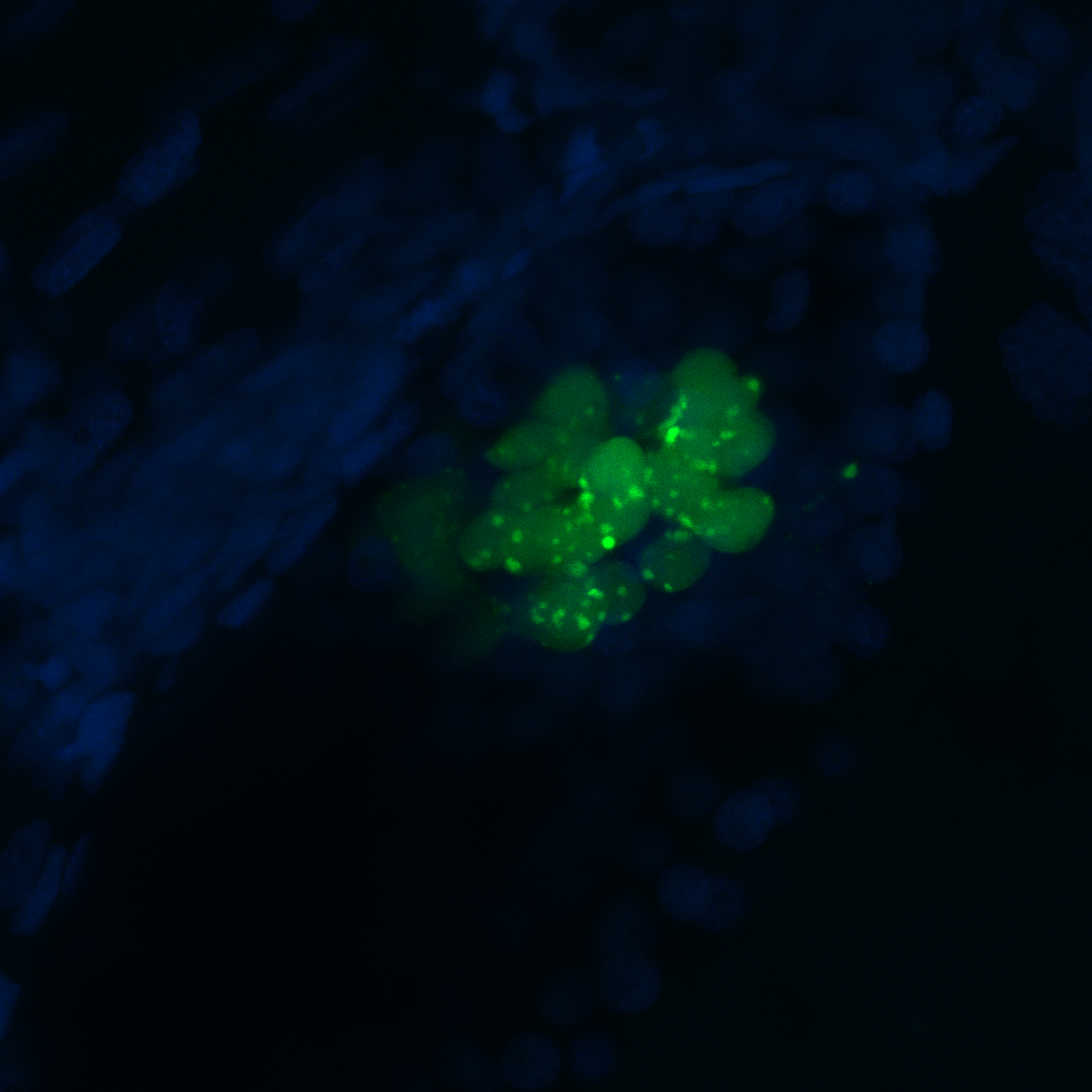

Supplement: Supplementary file 11 — Source data Fig. 4 [file 44318_2024_332_MOESM11_ESM.zip › Figure 4/4E/TRPV larvae composite.tif]

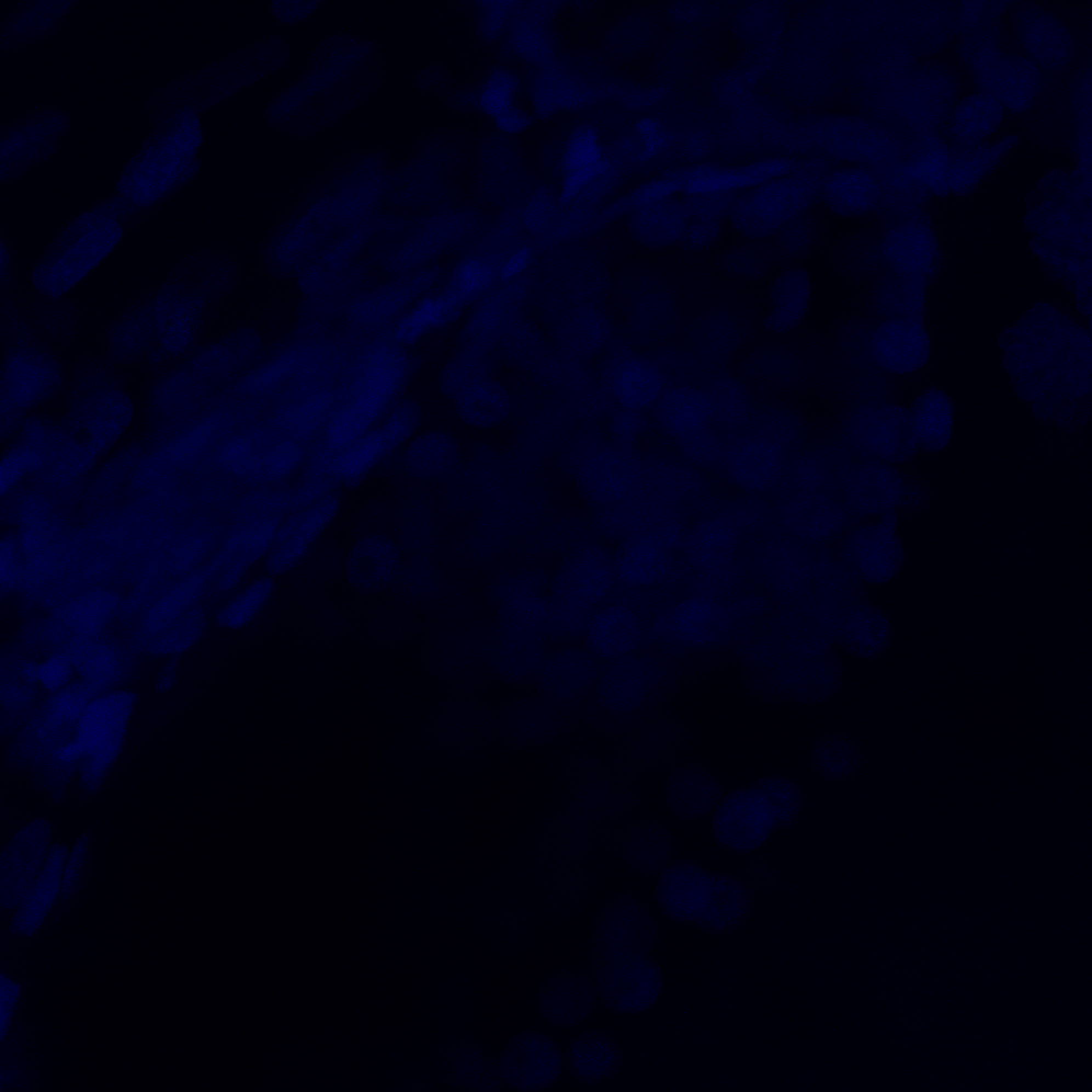

Supplement: Supplementary file 11 — Source data Fig. 4 [file 44318_2024_332_MOESM11_ESM.zip › Figure 4/4E/TRPV larvae Hoechst.tif]

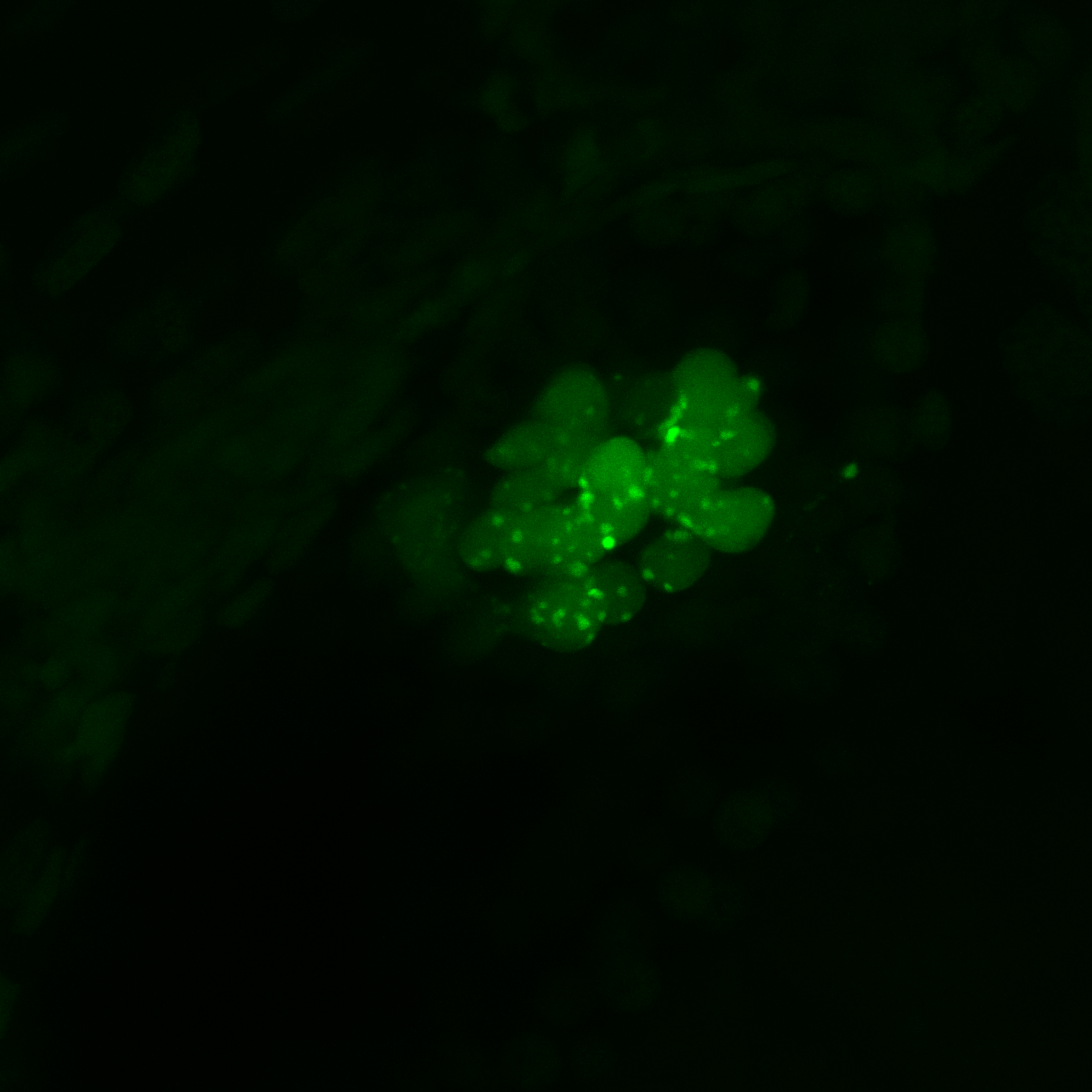

Supplement: Supplementary file 11 — Source data Fig. 4 [file 44318_2024_332_MOESM11_ESM.zip › Figure 4/4E/TRPV larvae Kaede.tif]

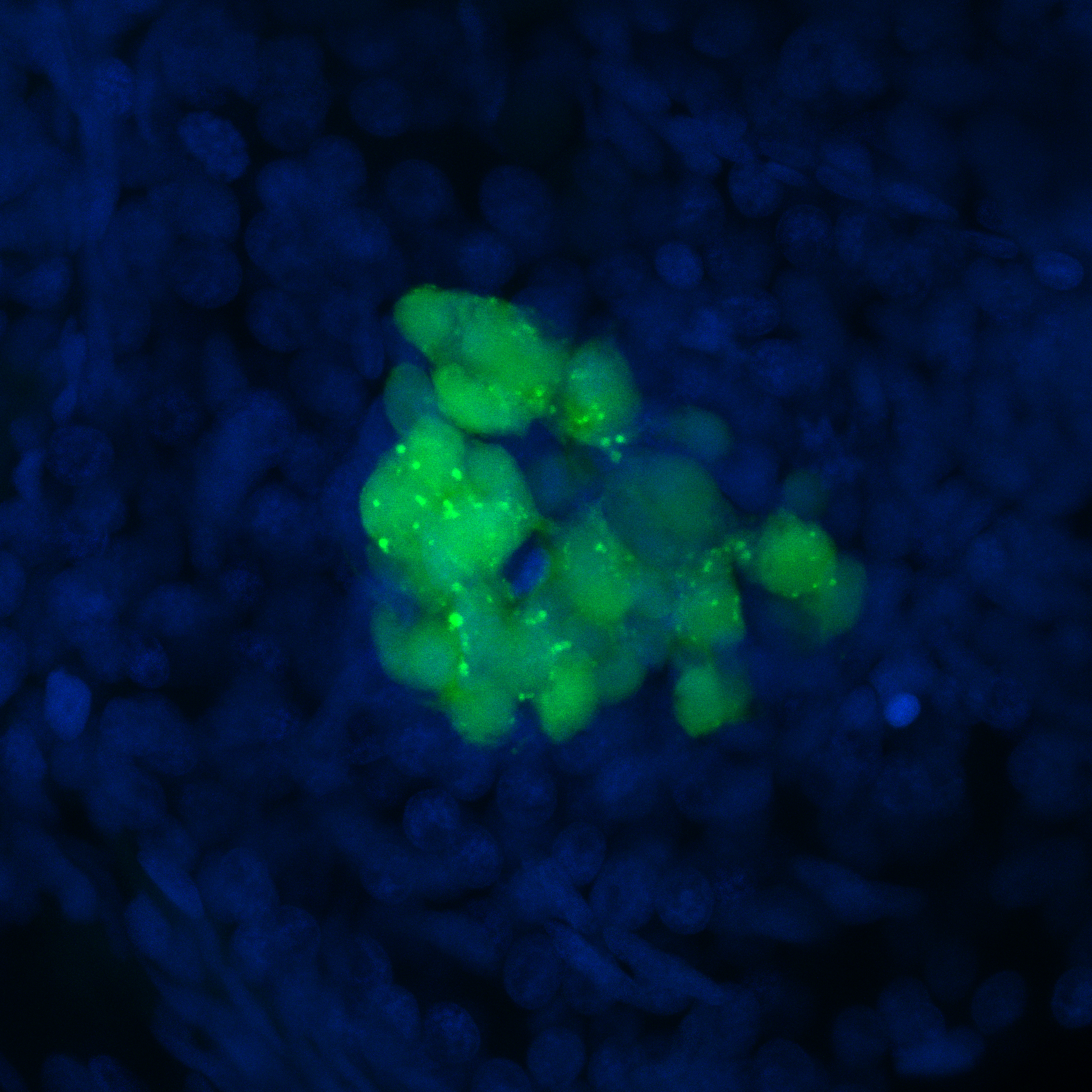

Supplement: Supplementary file 11 — Source data Fig. 4 [file 44318_2024_332_MOESM11_ESM.zip › Figure 4/4E/WT larvae composite.tif]

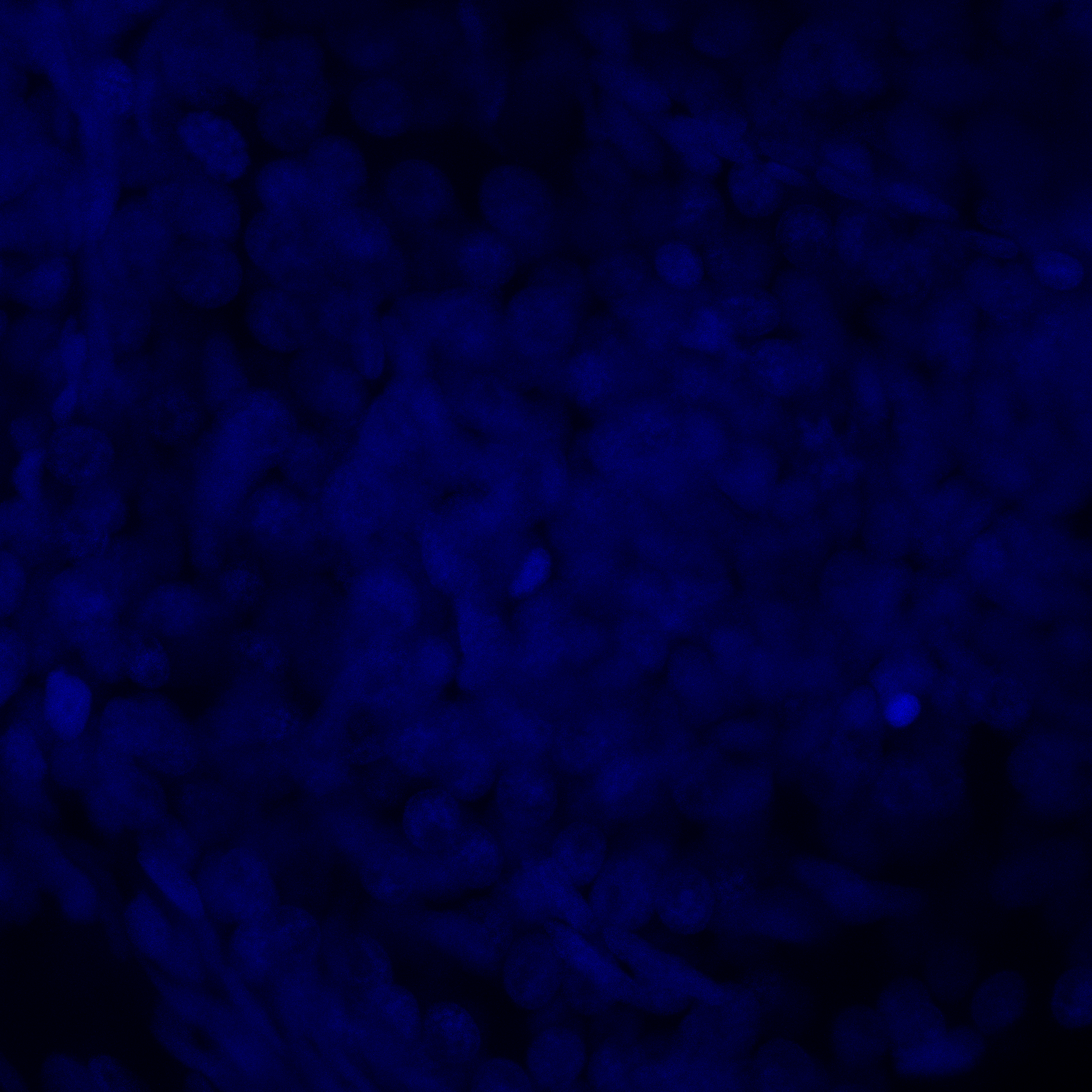

Supplement: Supplementary file 11 — Source data Fig. 4 [file 44318_2024_332_MOESM11_ESM.zip › Figure 4/4E/WT larvae Hoechst.tif]

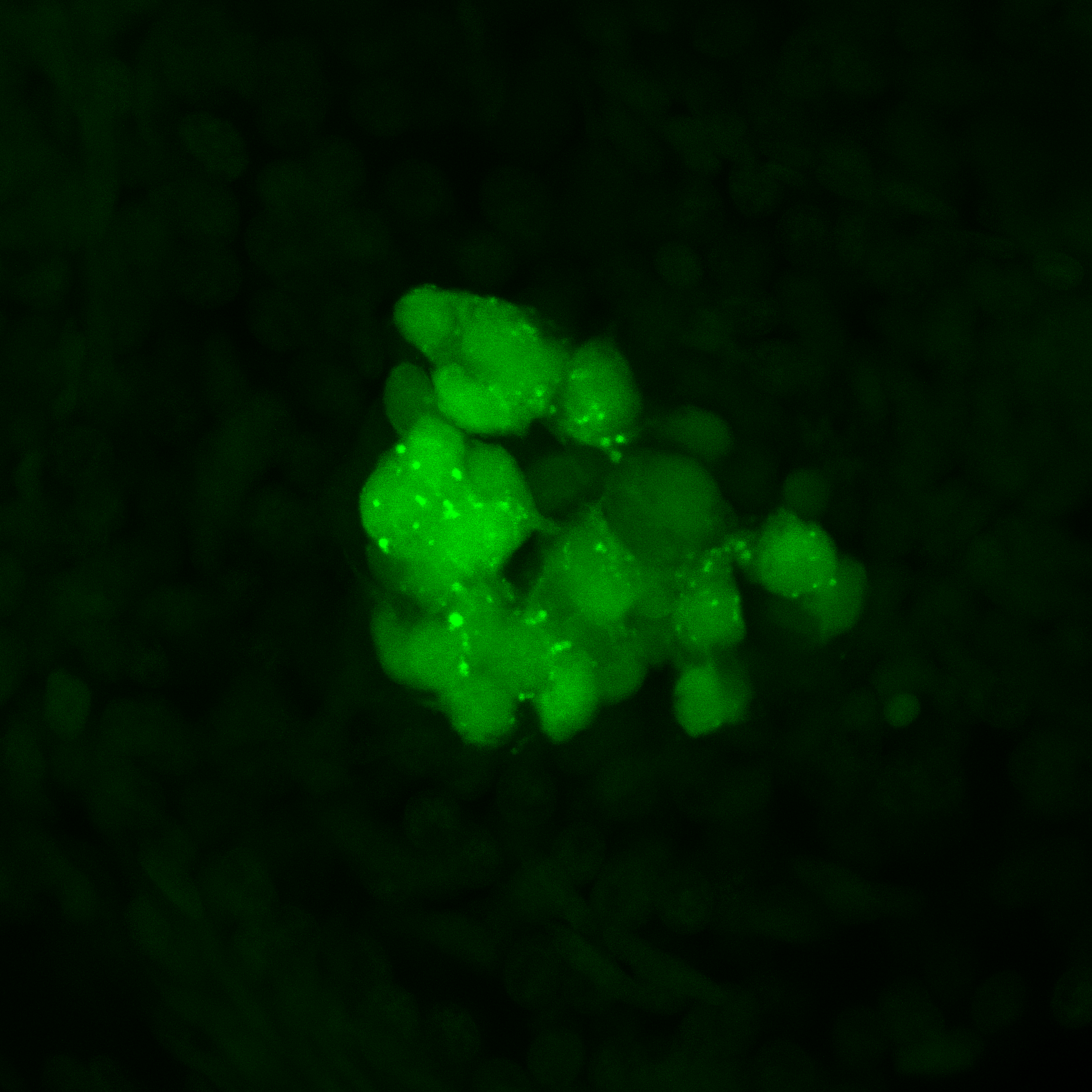

Supplement: Supplementary file 11 — Source data Fig. 4 [file 44318_2024_332_MOESM11_ESM.zip › Figure 4/4E/WT larvae Kaede.tif]

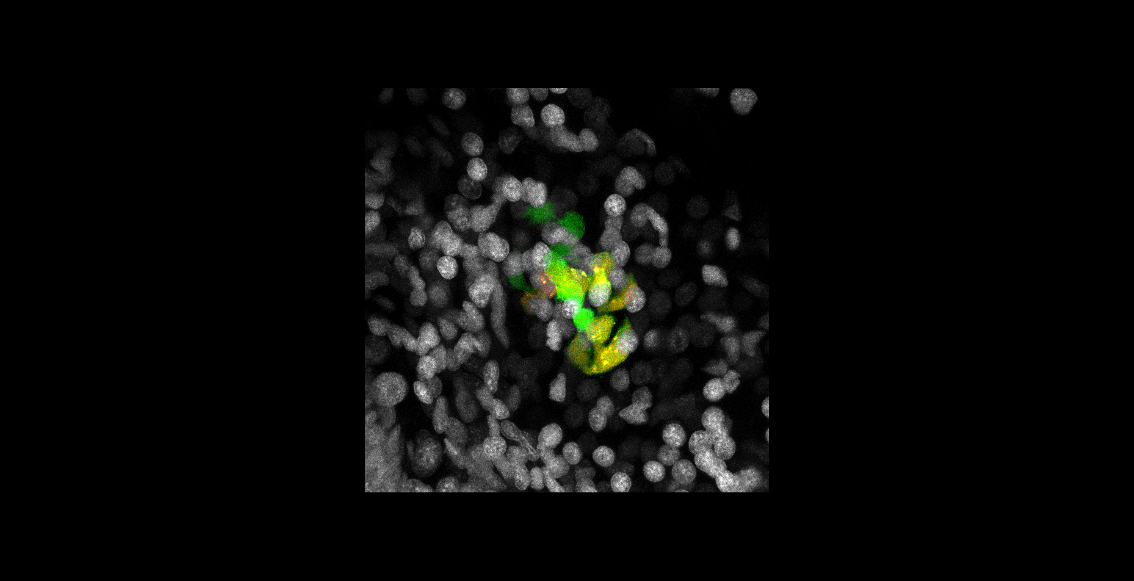

Supplement: Supplementary file 11 — Source data Fig. 4 [file 44318_2024_332_MOESM11_ESM.zip › Figure 4/4H/TRPV larvae composite.tif]

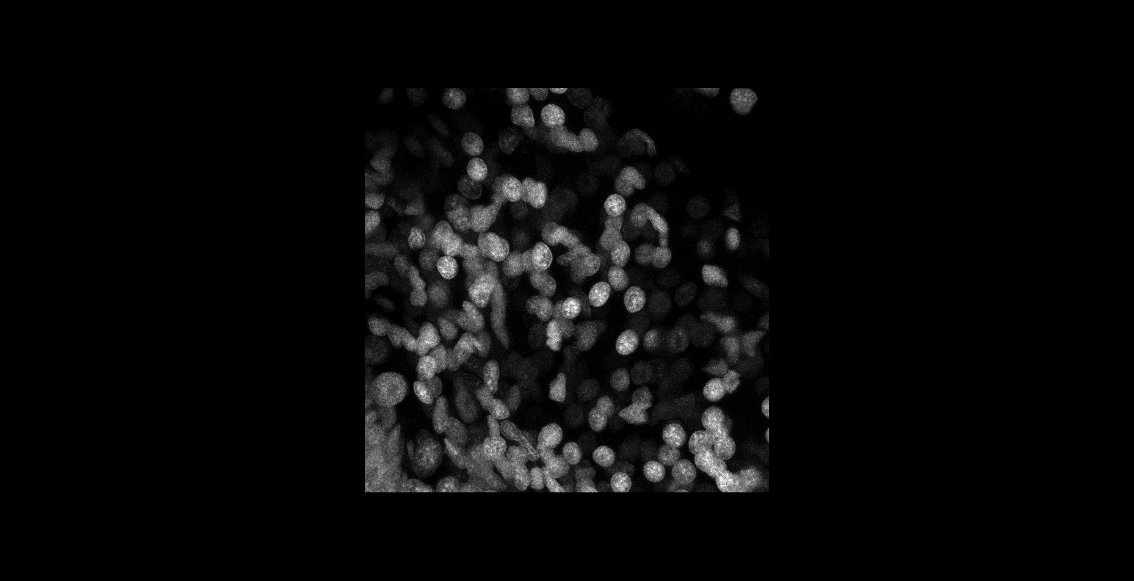

Supplement: Supplementary file 11 — Source data Fig. 4 [file 44318_2024_332_MOESM11_ESM.zip › Figure 4/4H/TRPV larvae EdU.tif]

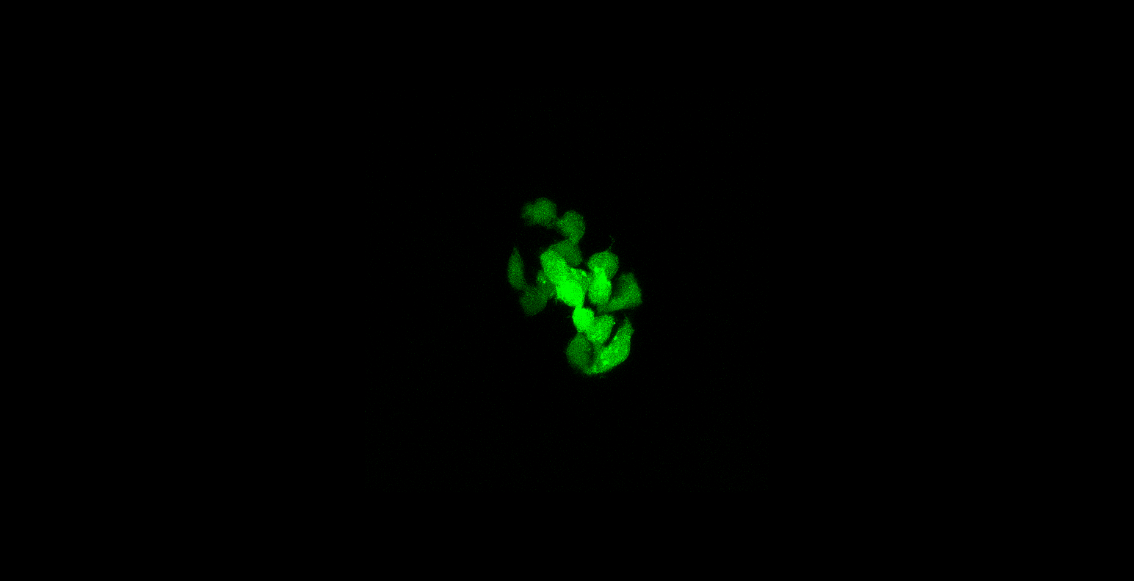

Supplement: Supplementary file 11 — Source data Fig. 4 [file 44318_2024_332_MOESM11_ESM.zip › Figure 4/4H/TRPV larvae Kaede Green.tif]

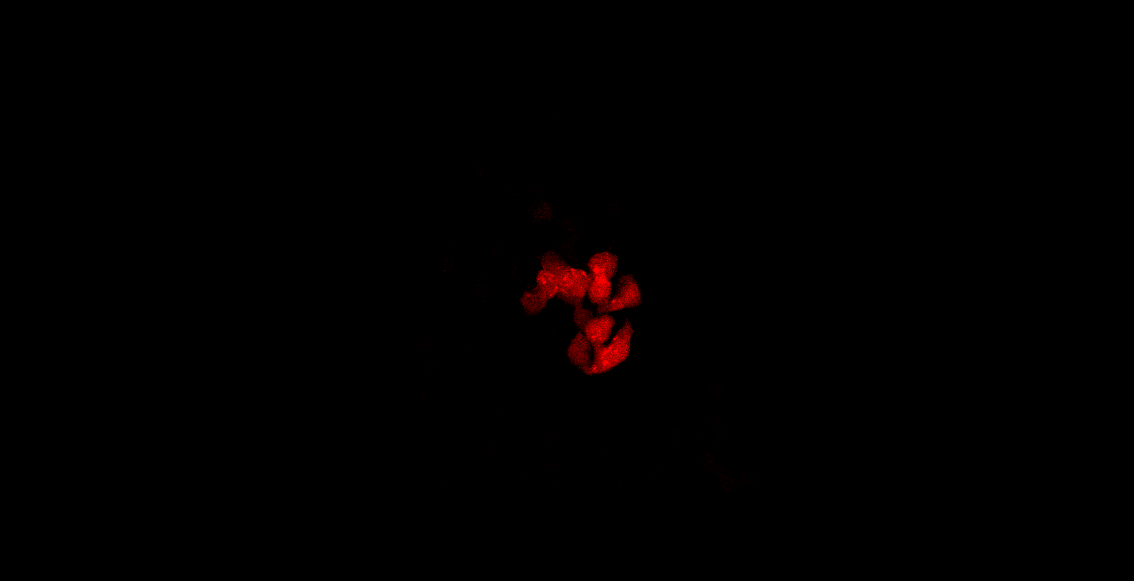

Supplement: Supplementary file 11 — Source data Fig. 4 [file 44318_2024_332_MOESM11_ESM.zip › Figure 4/4H/TRPV larvae Kaede Red.tif]

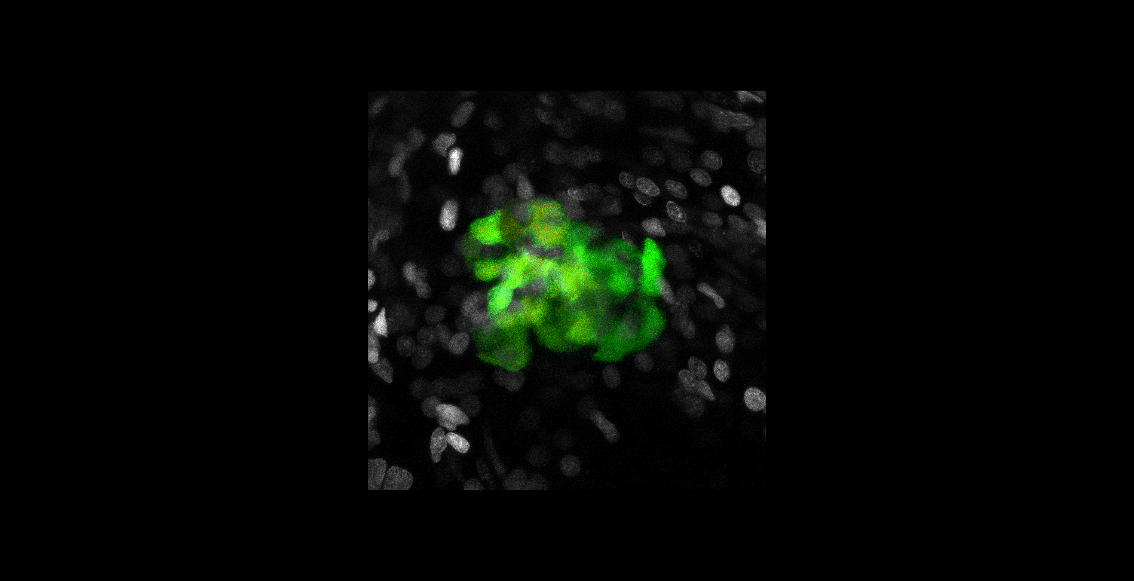

Supplement: Supplementary file 11 — Source data Fig. 4 [file 44318_2024_332_MOESM11_ESM.zip › Figure 4/4H/WT larvae composite.tif]

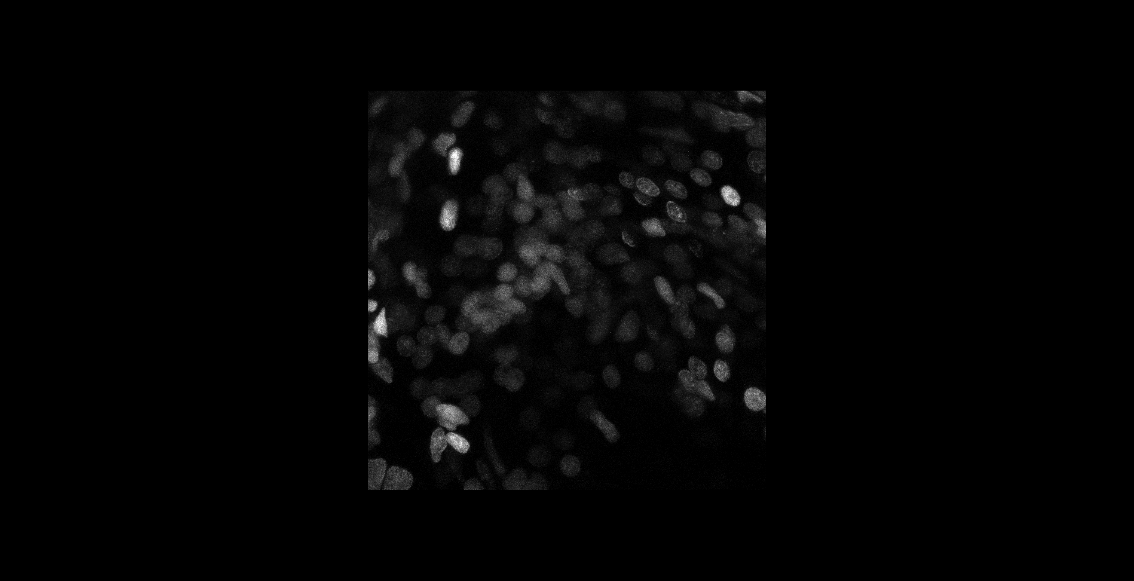

Supplement: Supplementary file 11 — Source data Fig. 4 [file 44318_2024_332_MOESM11_ESM.zip › Figure 4/4H/WT larvae EdU.tif]

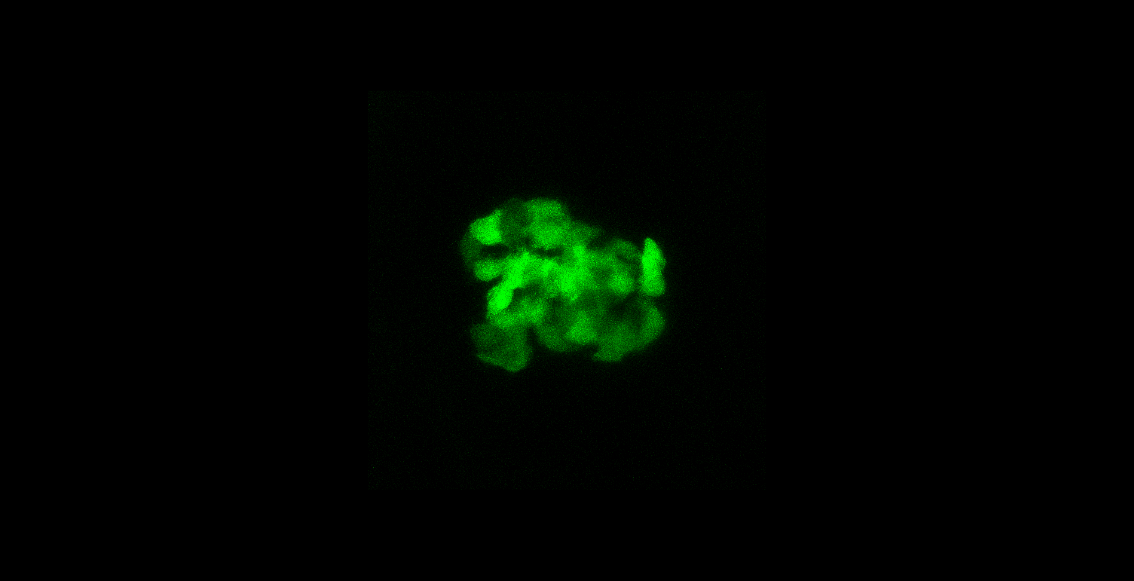

Supplement: Supplementary file 11 — Source data Fig. 4 [file 44318_2024_332_MOESM11_ESM.zip › Figure 4/4H/WT larvae Kaede Green.tif]

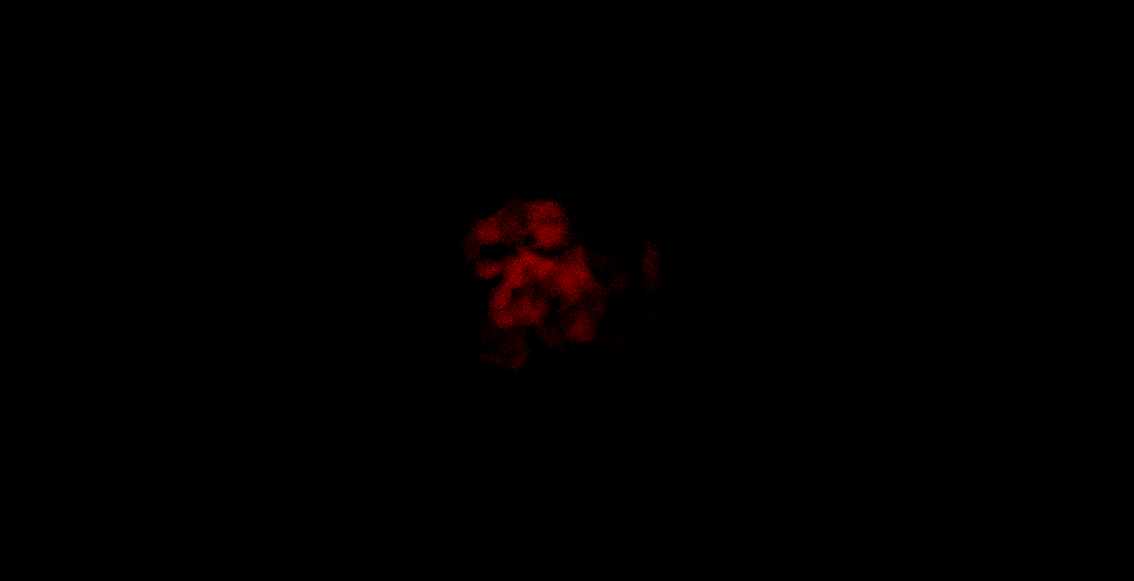

Supplement: Supplementary file 11 — Source data Fig. 4 [file 44318_2024_332_MOESM11_ESM.zip › Figure 4/4H/WT larvae Kaede Red.tif]

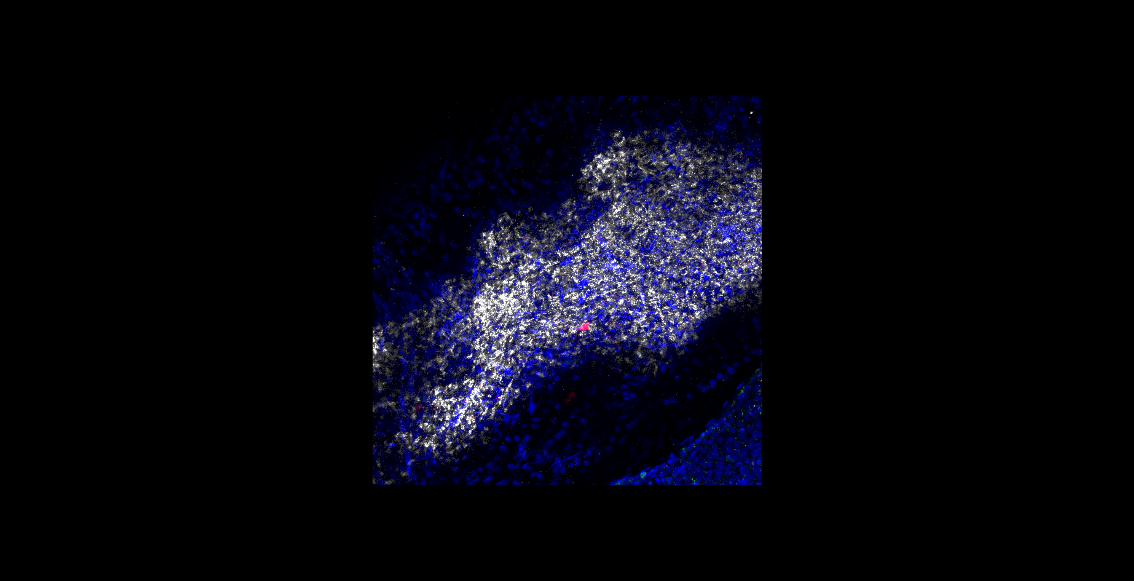

Supplement: Supplementary file 12 — Source data Fig. 6 [file 44318_2024_332_MOESM12_ESM.zip › Figure 6/A/p35 animal composite.tif]

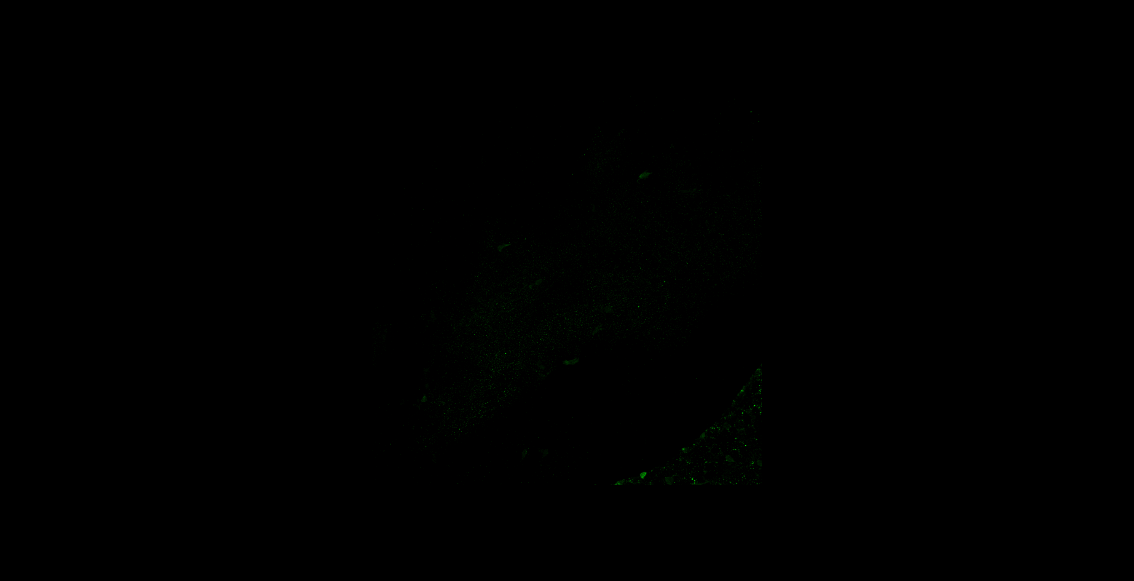

Supplement: Supplementary file 12 — Source data Fig. 6 [file 44318_2024_332_MOESM12_ESM.zip › Figure 6/A/p35 animal GFP.tif]

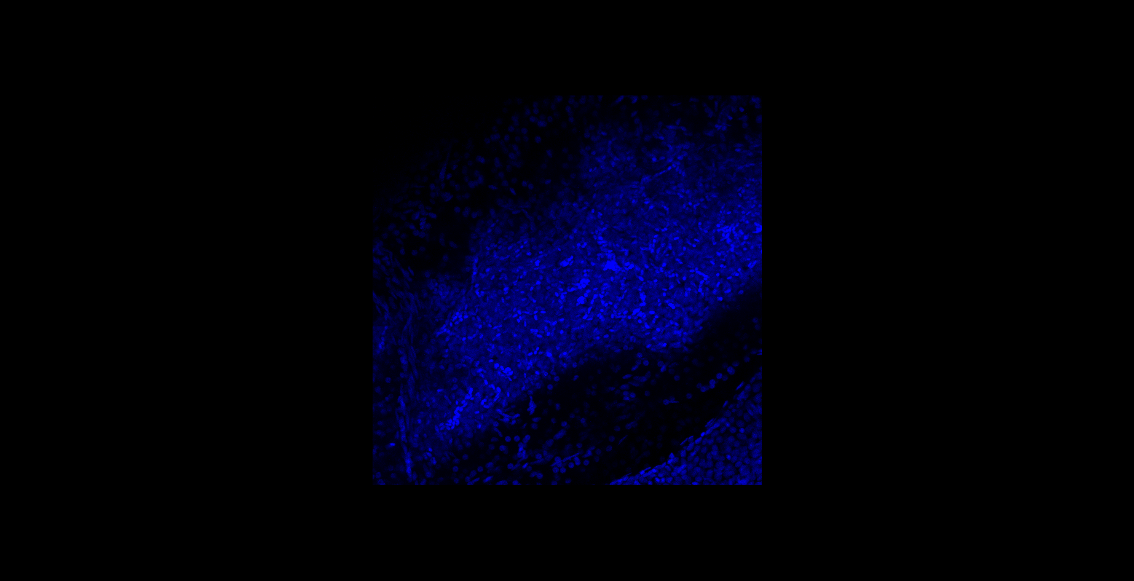

Supplement: Supplementary file 12 — Source data Fig. 6 [file 44318_2024_332_MOESM12_ESM.zip › Figure 6/A/p35 animal Hoechst.tif]

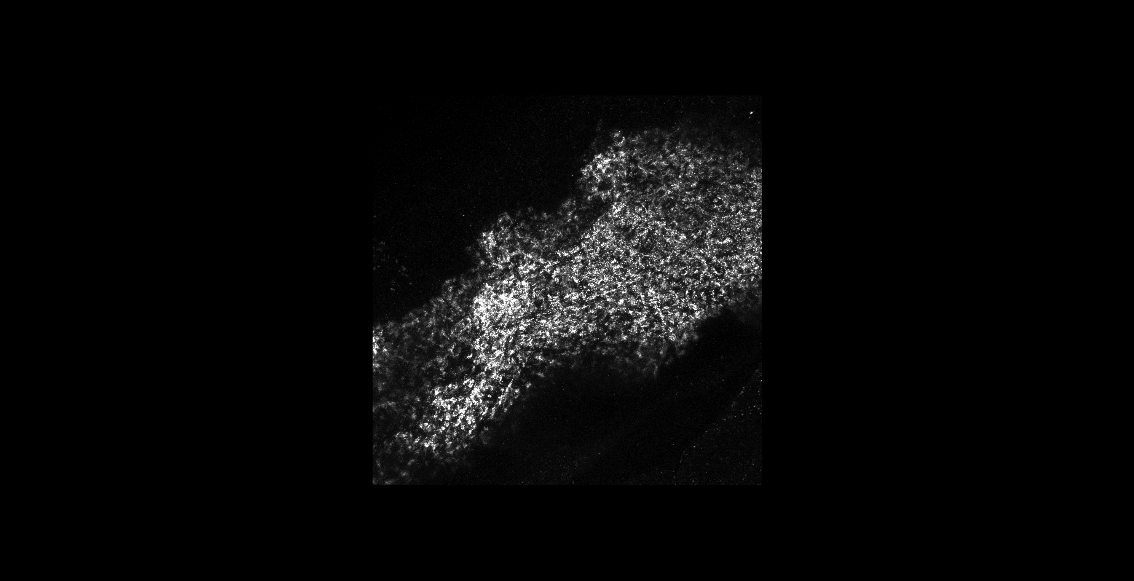

Supplement: Supplementary file 12 — Source data Fig. 6 [file 44318_2024_332_MOESM12_ESM.zip › Figure 6/A/p35 animal Ins.tif]

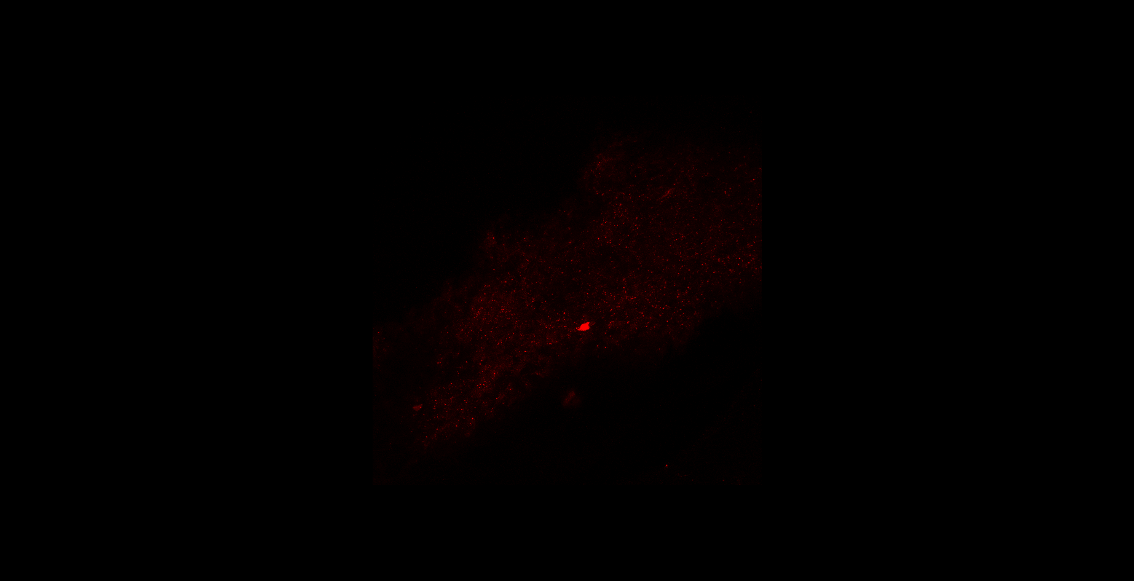

Supplement: Supplementary file 12 — Source data Fig. 6 [file 44318_2024_332_MOESM12_ESM.zip › Figure 6/A/p35 animal mCherry.tif]

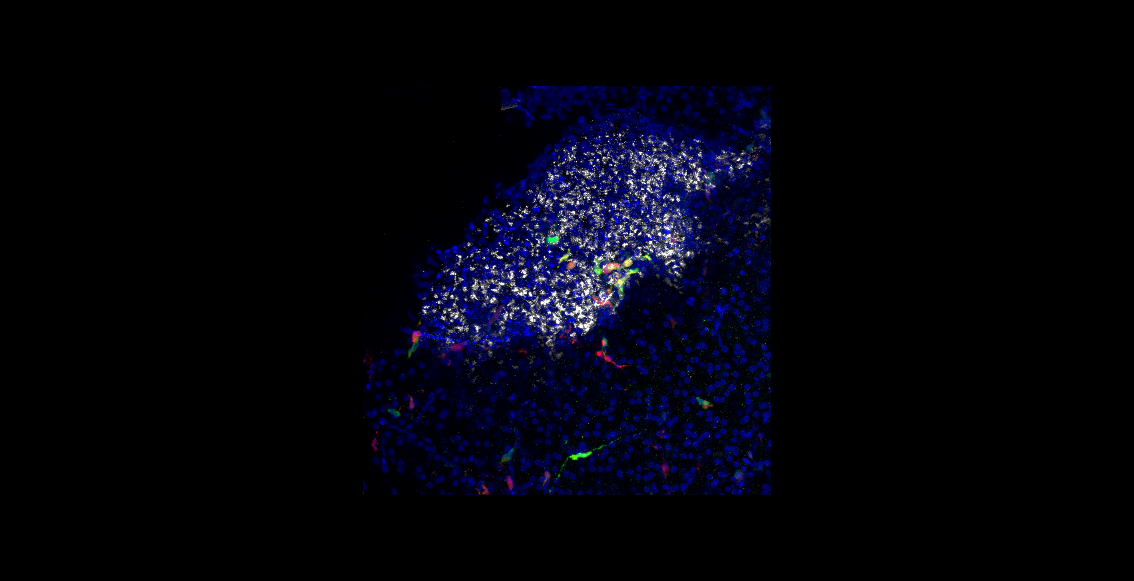

Supplement: Supplementary file 12 — Source data Fig. 6 [file 44318_2024_332_MOESM12_ESM.zip › Figure 6/A/WT animal composite.tif]

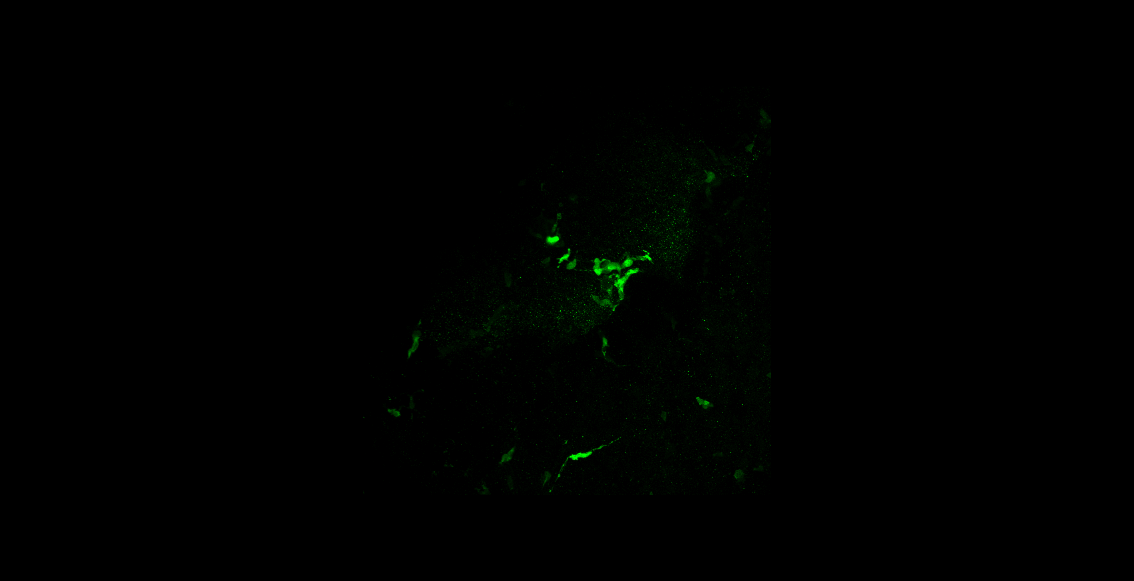

Supplement: Supplementary file 12 — Source data Fig. 6 [file 44318_2024_332_MOESM12_ESM.zip › Figure 6/A/WT animal GFP.tif]

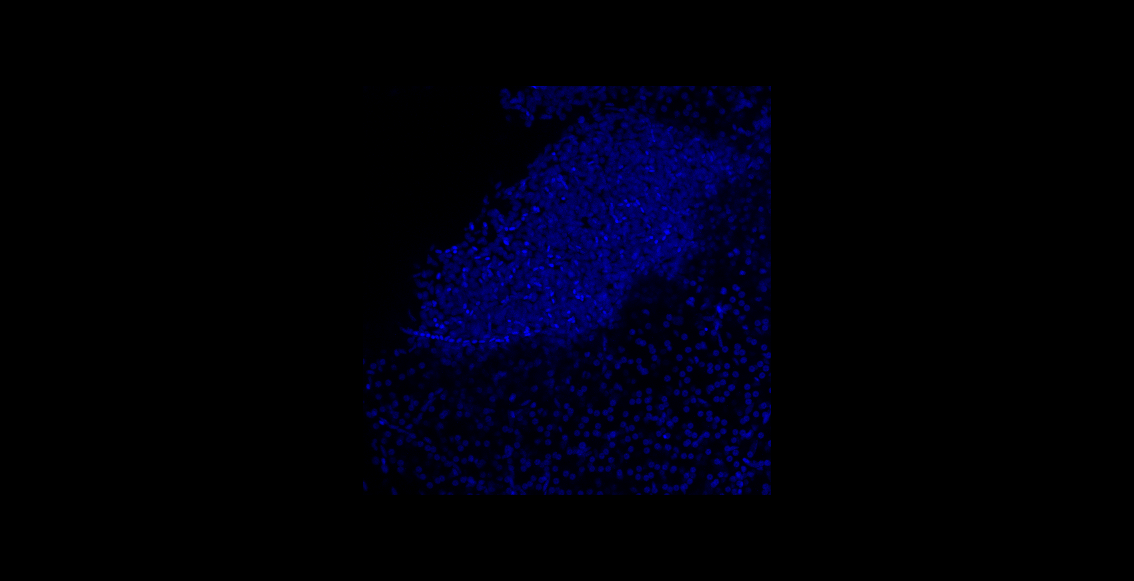

Supplement: Supplementary file 12 — Source data Fig. 6 [file 44318_2024_332_MOESM12_ESM.zip › Figure 6/A/WT animal Hoechst.tif]

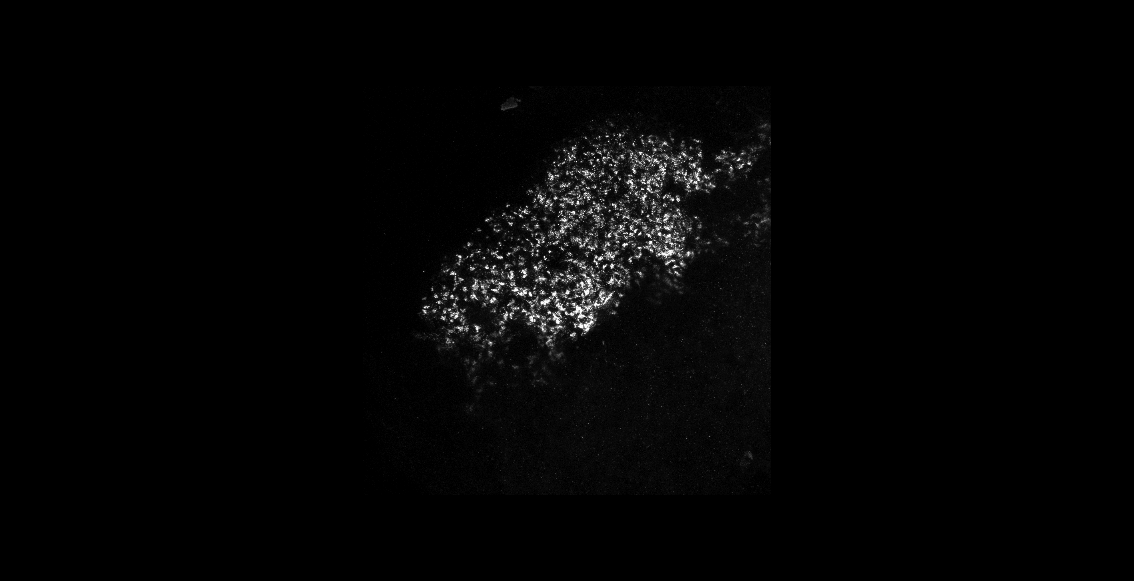

Supplement: Supplementary file 12 — Source data Fig. 6 [file 44318_2024_332_MOESM12_ESM.zip › Figure 6/A/WT animal Ins.tif]

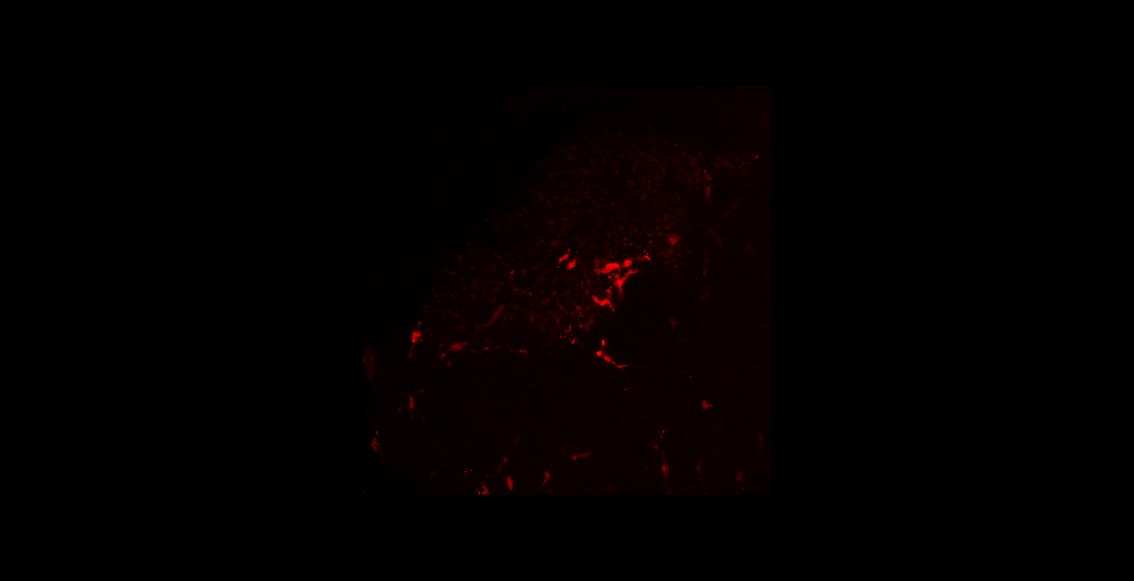

Supplement: Supplementary file 12 — Source data Fig. 6 [file 44318_2024_332_MOESM12_ESM.zip › Figure 6/A/WT animal mCherry.tif]

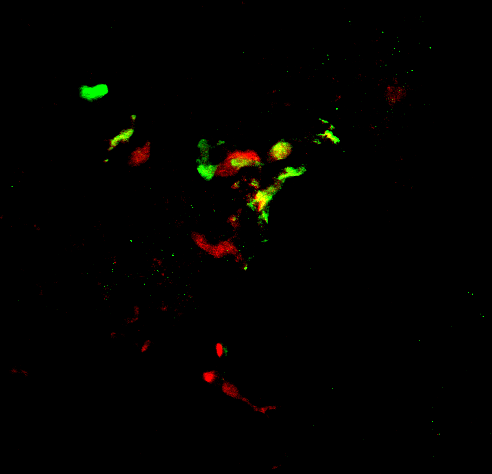

Supplement: Supplementary file 12 — Source data Fig. 6 [file 44318_2024_332_MOESM12_ESM.zip › Figure 6/A/zoom composite.tif]

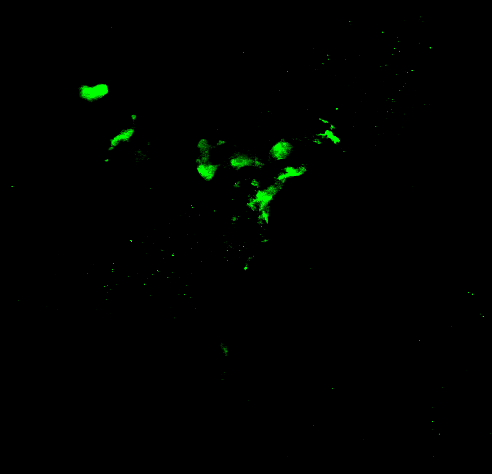

Supplement: Supplementary file 12 — Source data Fig. 6 [file 44318_2024_332_MOESM12_ESM.zip › Figure 6/A/zoom GFP.tif]

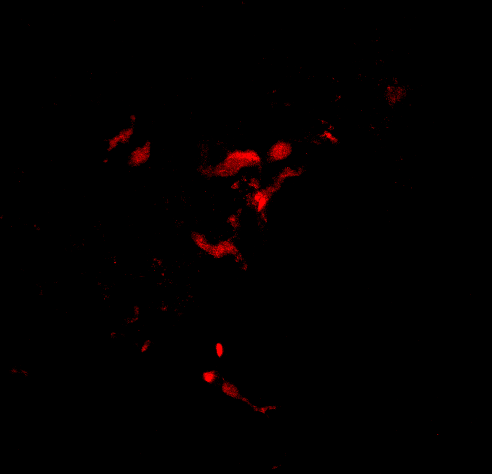

Supplement: Supplementary file 12 — Source data Fig. 6 [file 44318_2024_332_MOESM12_ESM.zip › Figure 6/A/zoom mCherry.tif]
