# Supplementary figures and images for: Developmental beta-cell death orchestrates the islet’s inflammatory milieu by regulating immune system crosstalk (part 2 of 2)
Source: EMBO J. 2025 Jan 6;44(4):1131–53. doi: 10.1038/s44318-024-00332-w (PMC11833124; doi:10.1038/s44318-024-00332-w)

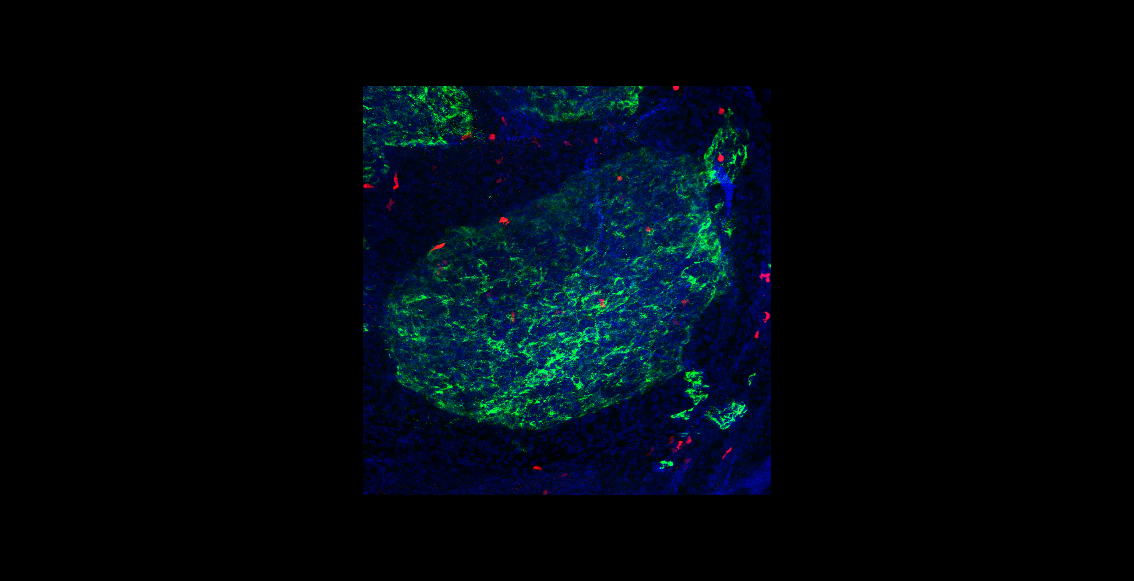

Supplement: Supplementary file 13 — Source data Fig. 7 [file 44318_2024_332_MOESM13_ESM.zip › Figure 7/7A/p35 animal composite.tif]

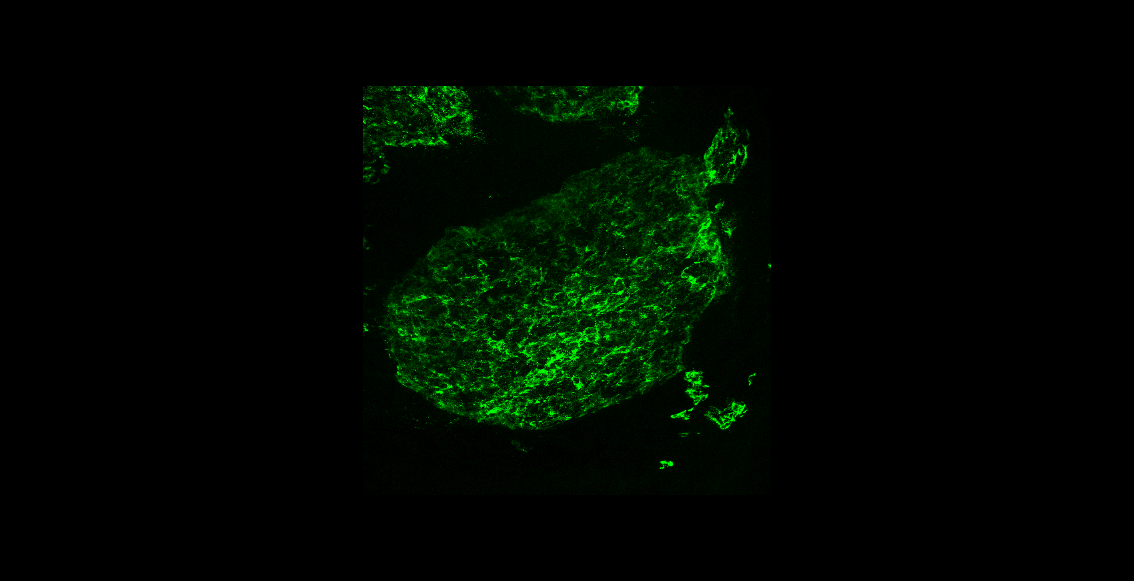

Supplement: Supplementary file 13 — Source data Fig. 7 [file 44318_2024_332_MOESM13_ESM.zip › Figure 7/7A/p35 animal Glucagon.tif]

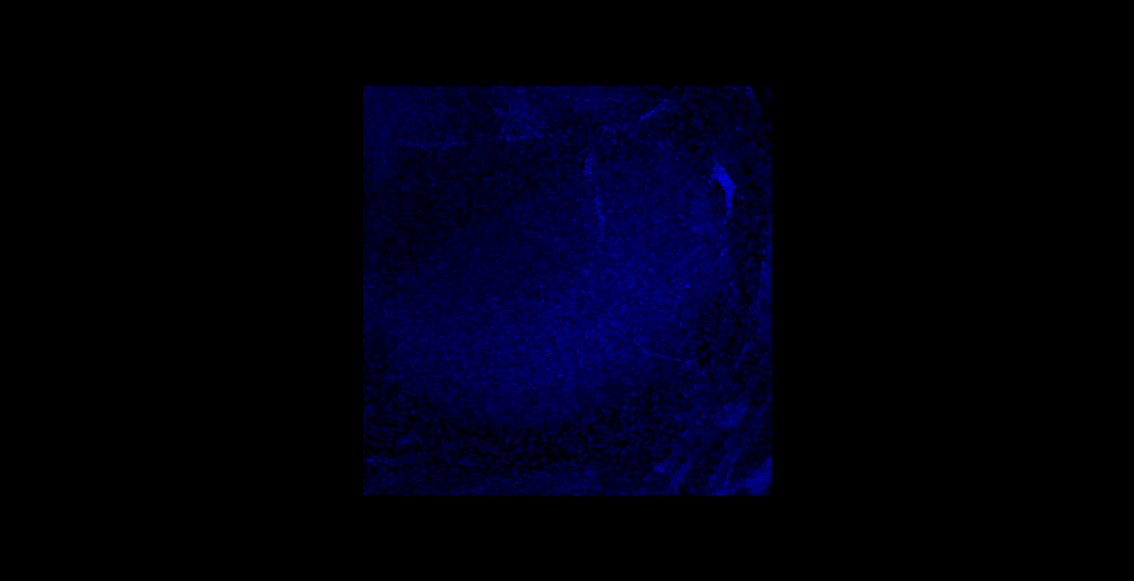

Supplement: Supplementary file 13 — Source data Fig. 7 [file 44318_2024_332_MOESM13_ESM.zip › Figure 7/7A/p35 animal Hoechst.tif]

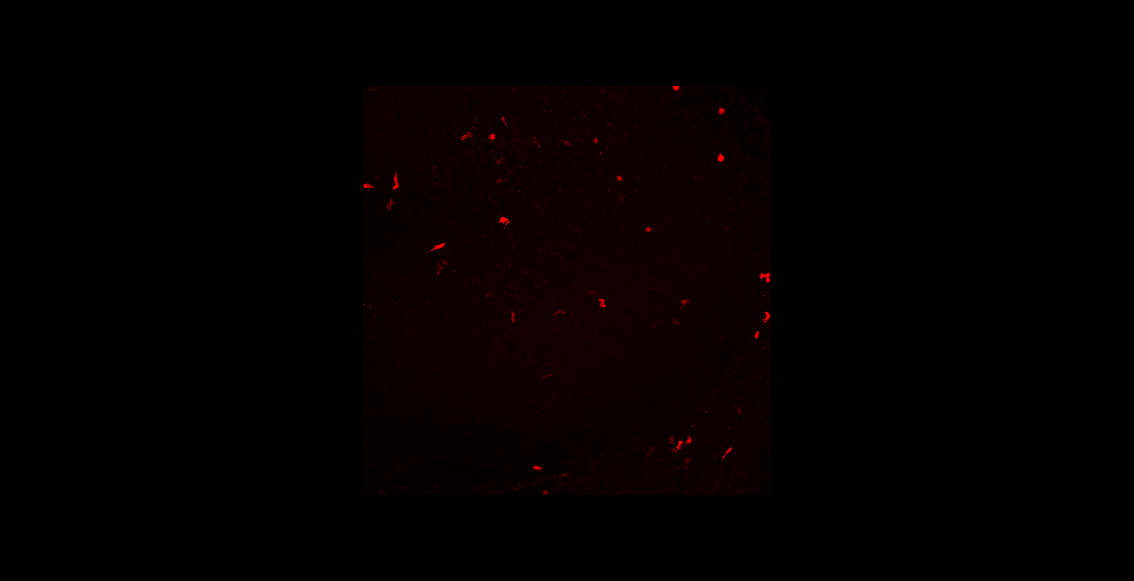

Supplement: Supplementary file 13 — Source data Fig. 7 [file 44318_2024_332_MOESM13_ESM.zip › Figure 7/7A/p35 animal RFP.tif]

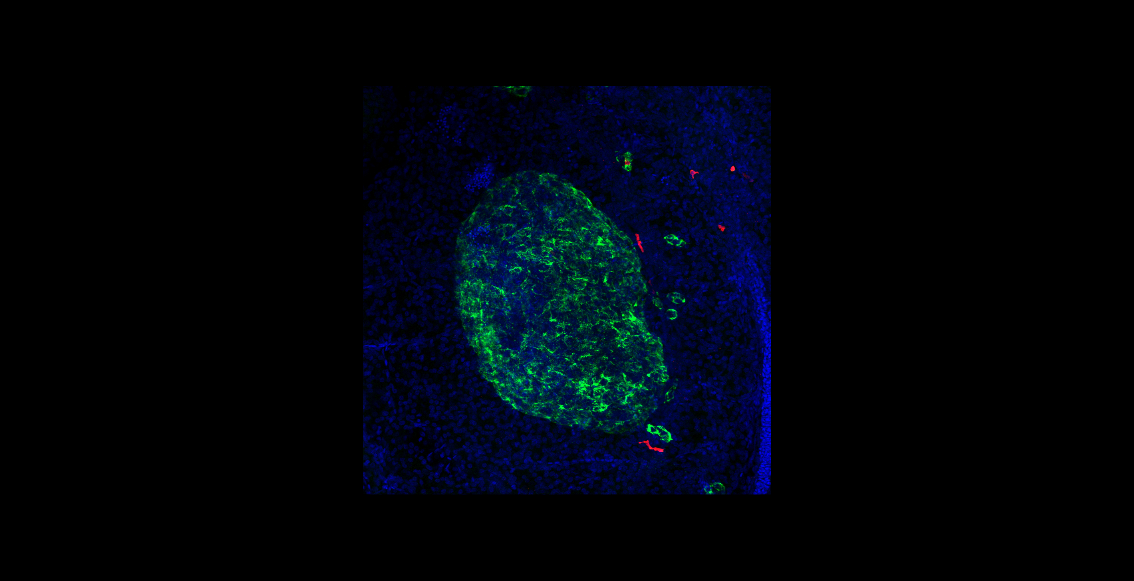

Supplement: Supplementary file 13 — Source data Fig. 7 [file 44318_2024_332_MOESM13_ESM.zip › Figure 7/7A/WT animal composite.tif]

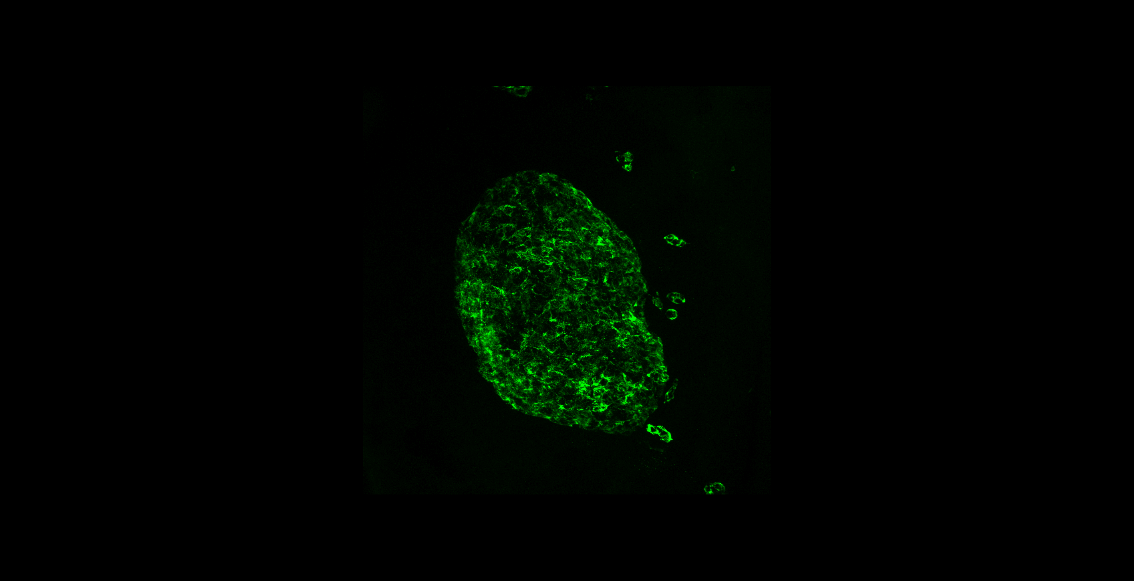

Supplement: Supplementary file 13 — Source data Fig. 7 [file 44318_2024_332_MOESM13_ESM.zip › Figure 7/7A/WT animal Glucagon.tif]

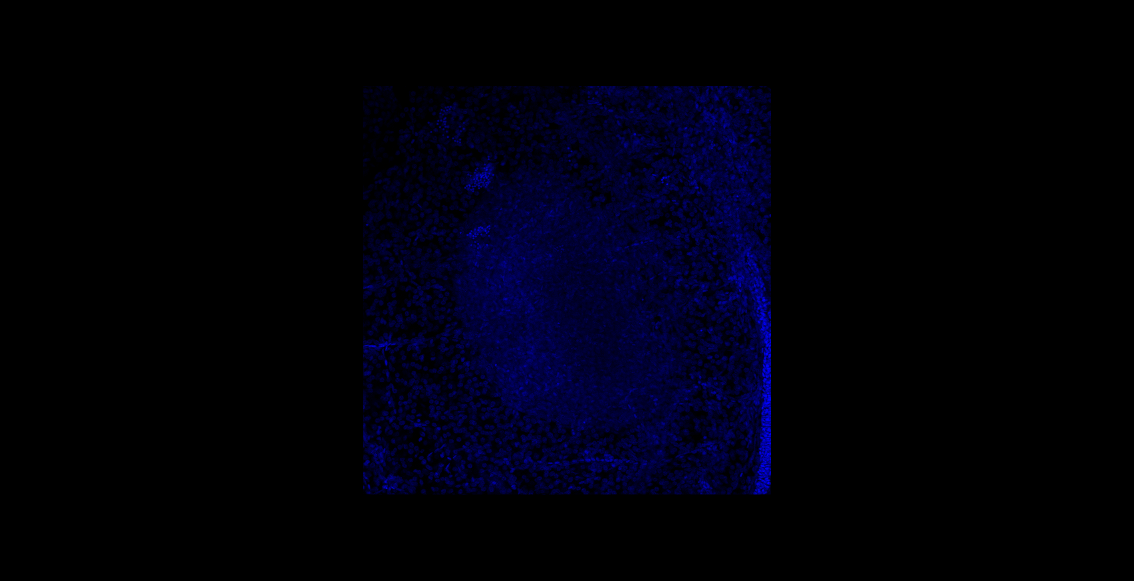

Supplement: Supplementary file 13 — Source data Fig. 7 [file 44318_2024_332_MOESM13_ESM.zip › Figure 7/7A/WT animal Hoechst.tif]

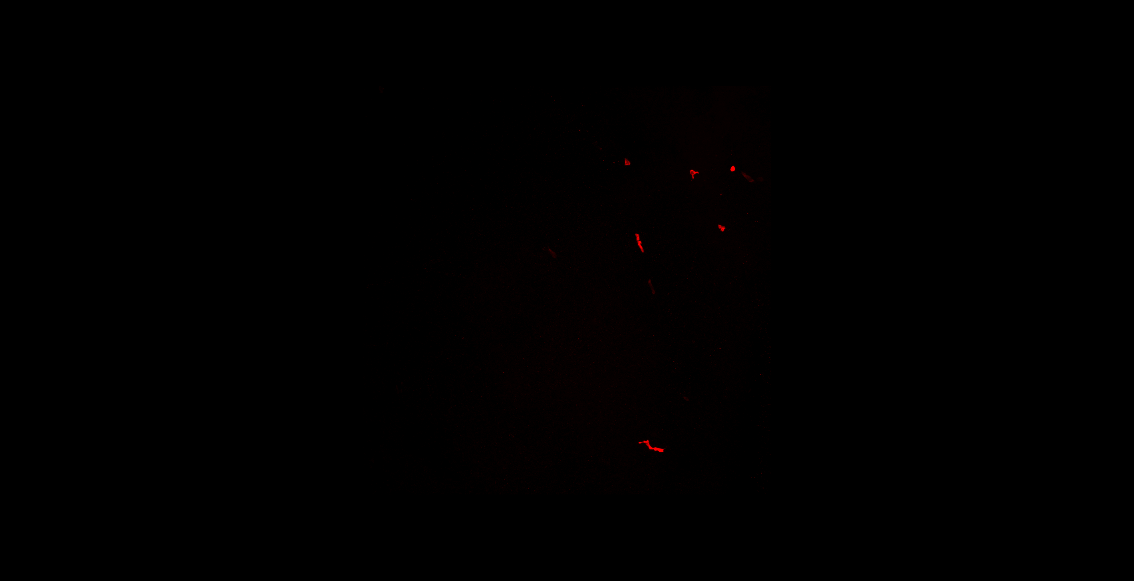

Supplement: Supplementary file 13 — Source data Fig. 7 [file 44318_2024_332_MOESM13_ESM.zip › Figure 7/7A/WT animal RFP.tif]
